# Supplementary material for: Unselfish traits and social decision-making patterns characterize six populations of real-world extraordinary altruists
Source: Nat Commun. 2023 Mar 31;14:1807. doi: 10.1038/s41467-023-37283-5 (PMC10066349; doi:10.1038/s41467-023-37283-5)
Supplement: Supplementary file 1 — Supplementary Information [file 41467_2023_37283_MOESM1_ESM.pdf]

Supplementary Information for:

Unselfish traits and social decision-making patterns characterize six populations of real-world extraordinary altruists

Shawn A. Rhoads[1*], Kruti M. Vekaria[1], Katherine O'Connell[1], Hannah S. Elizabeth[1], David G. Rand[2], Megan N. Kozak Williams[3], & Abigail A. Marsh[1*]

* Correspondence concerning this article should be addressed to Shawn A. Rhoads, Department of Psychology, Georgetown University, White-Gravenor Hall, 3700 P Street NW, Washington, DC 20057.

E-mail: sr1209@georgetown.edu

This PDF file includes:

Figures S1 to S8
Tables S1 to S18

This document contains 16 supplementary figures:

Figure S1a	Interpersonal reactivity traits across groups
Figure S1b	Risk taking traits across groups
Figure S1c	Risk taking perceptions across groups
Figure S1d	Psychopathic personality traits across groups
Figure S2	Altruist and control samples compared to 5,000 bootstrap means drawn from Lee & Ashton (2020)
Figure S3	Area under the receiver-operator characteristic curve plot
Figure S4a	Third-party ratings of altruism and risk
Figure S4b	Line plots estimated from third-party ratings of social discounting
Figure S4c	Third-party ratings of Honesty-Humility and Emotionality personality traits
Figure S4d	Third-party ratings of Extraversion and Agreeableness personality traits
Figure S4e	Third-party ratings of Conscientiousness and Openness to Experience personality traits
Figure S5a	BASE Jumper sample compared to 5,000 bootstrap means drawn from Lee & Ashton (2020)
Figure 5b	Miss America contestant sample compared to 5,000 bootstrap means drawn from Lee & Ashton (2020)
Figure S6	Representational similarity among trait perceptions and actual traits of altruists
Figure S7abc	Non-parametric statistical inference of representational similarity
Figure S8	Relationship between AUC and logk

This document contains 104 supplementary tables:

Table S1	Per capita prevalence estimates and demographics for each group among Americans in 2017
Table S2	Table of demographics of personality traits full-sample
Table S3a-i	Regression results for Honesty-Humility comparing altruistic groups to controls
Table S3a-ii	Regression results for Honesty-Humility comparing altruistic groups to controls
Table S3b-i	Regression results for Emotionality comparing altruistic groups to controls
Table S3b-ii	Regression results for Emotionality comparing altruistic groups to controls
Table S3c-i	Regression results for Extraversion comparing altruistic groups to controls
Table S3c-ii	Regression results for Extraversion comparing altruistic groups to controls
Table S3d-i	Regression results for Agreeableness comparing altruistic groups to controls
Table S3d-ii	Regression results for Agreeableness comparing altruistic groups to controls
Table S3e-i	Regression results for Conscientiousness comparing altruistic groups to controls
Table S3e-ii	Regression results for Conscientiousness comparing altruistic groups to controls
Table S3f-i	Regression results for Openness comparing altruistic groups to controls
Table S3f-ii	Regression results for Openness comparing altruistic groups to controls
Table S4a-i	Regression results for Risk Taking (Ethical) comparing altruistic groups to controls
Table S4a-ii	Regression results for Risk Taking (Ethical) comparing altruistic groups to controls
Table S4b-i	Regression results for Risk Taking (Financial) comparing altruistic groups to controls
Table S4b-ii	Regression results for Risk Taking (Financial) comparing altruistic groups to controls
Table S4c-i	Regression results for Risk Taking (Health Safety) comparing altruistic groups to controls
Table S4c-ii	Regression results for Risk Taking (Health Safety) comparing altruistic groups to controls
Table S4d-i	Regression results for Risk Taking (Recreational) comparing altruistic groups to controls
Table S4d-ii	Regression results for Risk Taking (Recreational) comparing altruistic groups to controls
Table S4e-i	Regression results for Risk Taking (Social) comparing altruistic groups to controls
Table S4e-ii	Regression results for Risk Taking (Social) comparing altruistic groups to controls
Table S5a-i	Regression results for Risk Taking Perception (Ethical) comparing altruistic groups to controls
Table S5a-ii	Regression results for Risk Taking Perception (Ethical) comparing altruistic groups to controls
Table S5b-i	Regression results for Risk Taking Perception (Financial) comparing altruistic groups to controls
Table S5b-ii	Regression results for Risk Taking Perception (Financial) comparing altruistic groups to controls
Table S5c-i	Regression results for Risk Taking Perception (Health Safety) comparing altruistic groups to controls
Table S5c-ii	Regression results for Risk Taking Perception (Health Safety) comparing altruistic groups to controls
Table S5d-i	Regression results for Risk Taking Perception (Recreational) comparing altruistic groups to controls
Table S5d-ii	Regression results for Risk Taking Perception (Recreational) comparing altruistic groups to controls
Table S5e-i	Regression results for Risk Taking Perception (Social) comparing altruistic groups to controls
Table S5e-ii	Regression results for Risk Taking Perception (Social) comparing altruistic groups to controls
Table S6a-i	Regression results for Perspective Taking comparing altruistic groups to controls
Table S6a-ii	Regression results for Perspective Taking comparing altruistic groups to controls
Table S6b-i	Regression results for Fantasy comparing altruistic groups to controls
Table S6b-ii	Regression results for Fantasy comparing altruistic groups to controls
Table S6c-i	Regression results for Empathic Concern comparing altruistic groups to controls
Table S6c-ii	Regression results for Empathic Concern comparing altruistic groups to controls
Table S6d-i	Regression results for Personal Distress comparing altruistic groups to controls
Table S6d-ii	Regression results for Personal Distress comparing altruistic groups to controls
Table S7a-i	Regression results for Fearless Dominance comparing altruistic groups to controls

Table S7a-ii	Regression results for Fearless Dominance comparing altruistic groups to controls
Table S7b-i	Regression results for Coldheartedness comparing altruistic groups to controls
Table S7b-ii	Regression results for Coldheartedness comparing altruistic groups to controls
Table S7c-i	Regression results for Self-centered Impulsivity comparing altruistic groups to controls
Table S7c-ii	Regression results for Self-centered Impulsivity comparing altruistic groups to controls
Table S8-i	Regression results for CRT comparing altruistic groups to controls
Table S8-ii	Regression results for CRT comparing altruistic groups to controls
Table S10a-i	Regression results for amount willing to forgo for N=1 comparing altruistic groups to controls
Table S10a-ii	Regression results for amount willing to forgo for N=1 comparing altruistic groups to controls
Table S10b-i	Regression results for amount willing to forgo for N=2 comparing altruistic groups to controls
Table S10b-ii	Regression results for amount willing to forgo for N=2 comparing altruistic groups to controls
Table S10c-i	Regression results for amount willing to forgo for N=5 comparing altruistic groups to controls
Table S10c-ii	Regression results for amount willing to forgo for N=5 comparing altruistic groups to controls
Table S10d-i	Regression results for amount willing to forgo for N=10 comparing altruistic groups to controls
Table S10d-ii	Regression results for amount willing to forgo for N=10 comparing altruistic groups to controls
Table S10e-i	Regression results for amount willing to forgo for N=20 comparing altruistic groups to controls
Table S10e-ii	Regression results for amount willing to forgo for N=20 comparing altruistic groups to controls
Table S10f-i	Regression results for amount willing to forgo for N=50 comparing altruistic groups to controls
Table S10f-ii	Regression results for amount willing to forgo for N=50 comparing altruistic groups to controls
Table S10g-i	Regression results for amount willing to forgo for N=100 comparing altruistic groups to controls
Table S10g-ii	Regression results for amount willing to forgo for N=100 comparing altruistic groups to controls
Table S11	Hyperbolic mixed-effects model results for group differences in social discounting
Table S12	Table of demographics of social discounting sub-sample (for classification analysis)
Table S13	Means and standard deviations of trait and behavioral measures among altruists and controls
Table S14	Table of bivariate Spearman rho correlations among variables of interest
Table S15	Table of demographics of exploratory perceptions samples
Table S16a	Definitions of groups included in the independent study of altruistic perceptions
Table S16b	Definitions of characteristics included in the independent study of altruistic perceptions
Table S17a-i	Linear mixed-effects results for beliefs about altruistic groups' Altruism (Study 2a)
Table S17a-ii	Linear mixed-effects results for beliefs about altruistic groups' Altruism (Study 2b)
Table S17b-i	Linear mixed-effects results for beliefs about altruistic groups' Risk (Study 2a)
Table S17b-ii	Linear mixed-effects results for beliefs about altruistic groups' Risk (Study 2b)
Table S17c-i	Linear mixed-effects results for beliefs about altruistic groups' Social Discounting (Study 2a)
Table S17c-ii	Linear mixed-effects results for beliefs about altruistic groups' Social Discounting (Study 2b)
Table S17d-i	Linear mixed-effects results for beliefs about altruistic groups' Honesty-Humility (Study 2a)
Table S17d-ii	Linear mixed-effects results for beliefs about altruistic groups' Honesty-Humility (Study 2b)
Table S17e-i	Linear mixed-effects results for beliefs about altruistic groups' Emotionality (Study 2a)
Table S17e-ii	Linear mixed-effects results for beliefs about altruistic groups' Emotionality (Study 2b)
Table S17f-i	Linear mixed-effects results for beliefs about altruistic groups' Extraversion (Study 2a)
Table S17f-ii	Linear mixed-effects results for beliefs about altruistic groups' Extraversion (Study 2b)
Table S17g-i	Linear mixed-effects results for beliefs about altruistic groups' Agreeableness (Study 2a)
Table S17g-ii	Linear mixed-effects results for beliefs about altruistic groups' Agreeableness (Study 2b)
Table S17h-i	Linear mixed-effects results for beliefs about altruistic groups' Conscientiousness (Study 2a)
Table S17h-ii	Linear mixed-effects results for beliefs about altruistic groups' Conscientiousness (Study 2b)
Table S17i-i	Linear mixed-effects results for beliefs about altruistic groups' Openness to Experience (Study 2a)
Table S18a-i	Linear mixed-effects results for beliefs about Miss America Contestants' Altruism
Table S18a-ii	Linear mixed-effects results for beliefs about BASE Jumpers' Altruism
Table S18b-i	Linear mixed-effects results for beliefs about Miss America Contestants' Risk
Table S18b-ii	Linear mixed-effects results for beliefs about BASE Jumpers' Risk
Table S18c-i	Linear mixed-effects results for beliefs about Miss America Contestants' Honesty-Humility
Table S18c-ii	Linear mixed-effects results for beliefs about BASE Jumpers' Honesty-Humility
Table S18d-i	Linear mixed-effects results for beliefs about Miss America Contestants' Emotionality
Table S18d-ii	Linear mixed-effects results for beliefs about BASE Jumpers' Emotionality
Table S18e-i	Linear mixed-effects results for beliefs about Miss America Contestants' Extraversion
Table S18e-ii	Linear mixed-effects results for beliefs about BASE Jumpers' Extraversion
Table S18f-i	Linear mixed-effects results for beliefs about Miss America Contestants' Agreeableness
Table S18f-ii	Linear mixed-effects results for beliefs about BASE Jumpers' Agreeableness
Table S18g-i	Linear mixed-effects results for beliefs about Miss America Contestants' Conscientiousness
Table S18g-ii	Linear mixed-effects results for beliefs about BASE Jumpers' Conscientiousness
Table S18h-i	Linear mixed-effects results for beliefs about Miss America Contestants' Openness to Experience
Table S18h-ii	Linear mixed-effects results for beliefs about BASE Jumpers' Openness to Experience

Figure S1a. Interpersonal reactivity traits across groups.

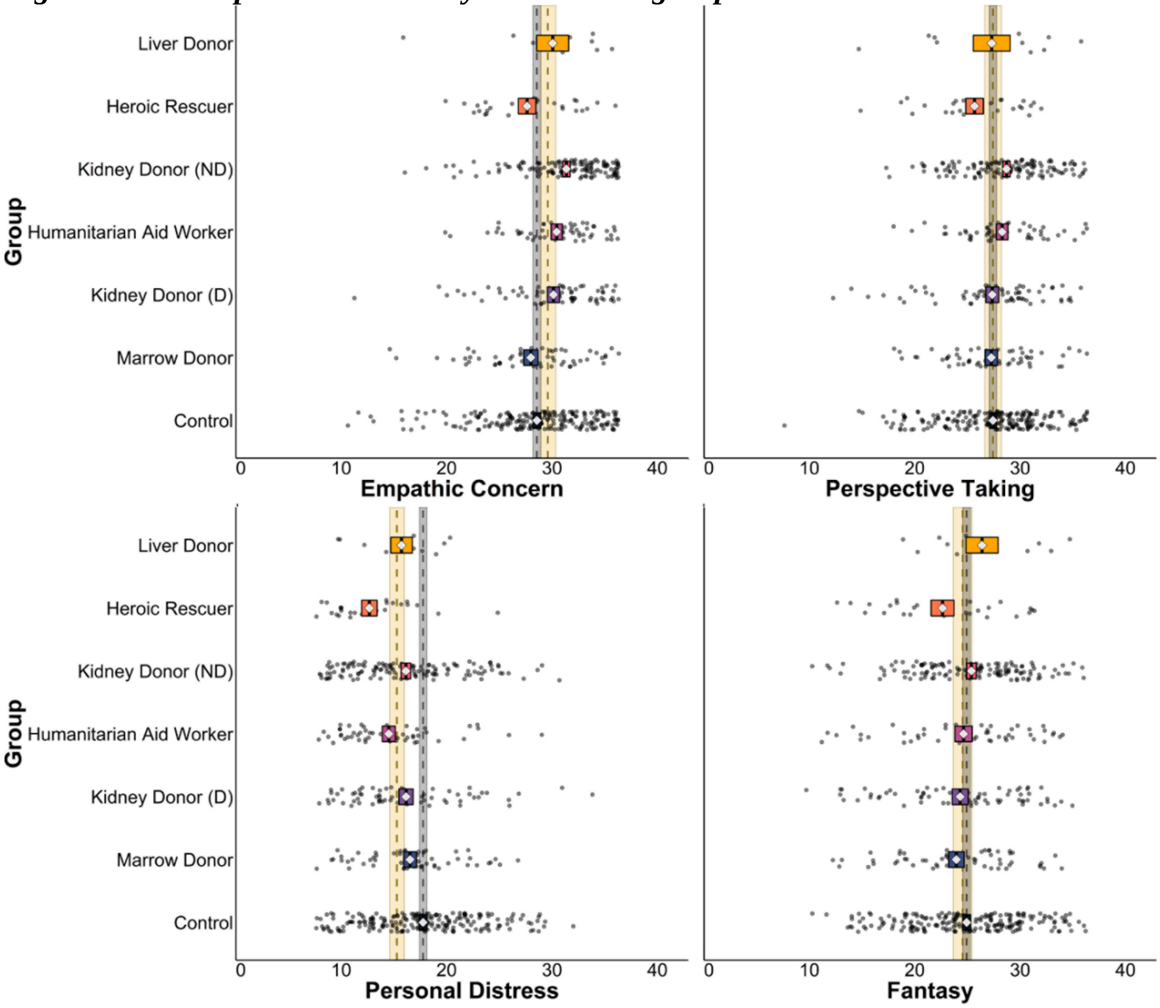

Note. Gray dashed line and ribbon represent control group mean and 95% CIs (N=207), yellow dashed line and ribbon represent combined altruist group mean and 95% CIs (N=347), diamonds represent means of individual groups, box widths represent 95% CIs. Multivariate regressions compared all altruistic groups against controls simultaneously using two-sided tests (in lieu of separate tests for which corrections for multiple comparisons would be appropriate).

Figure S1b. Risk taking traits across groups.

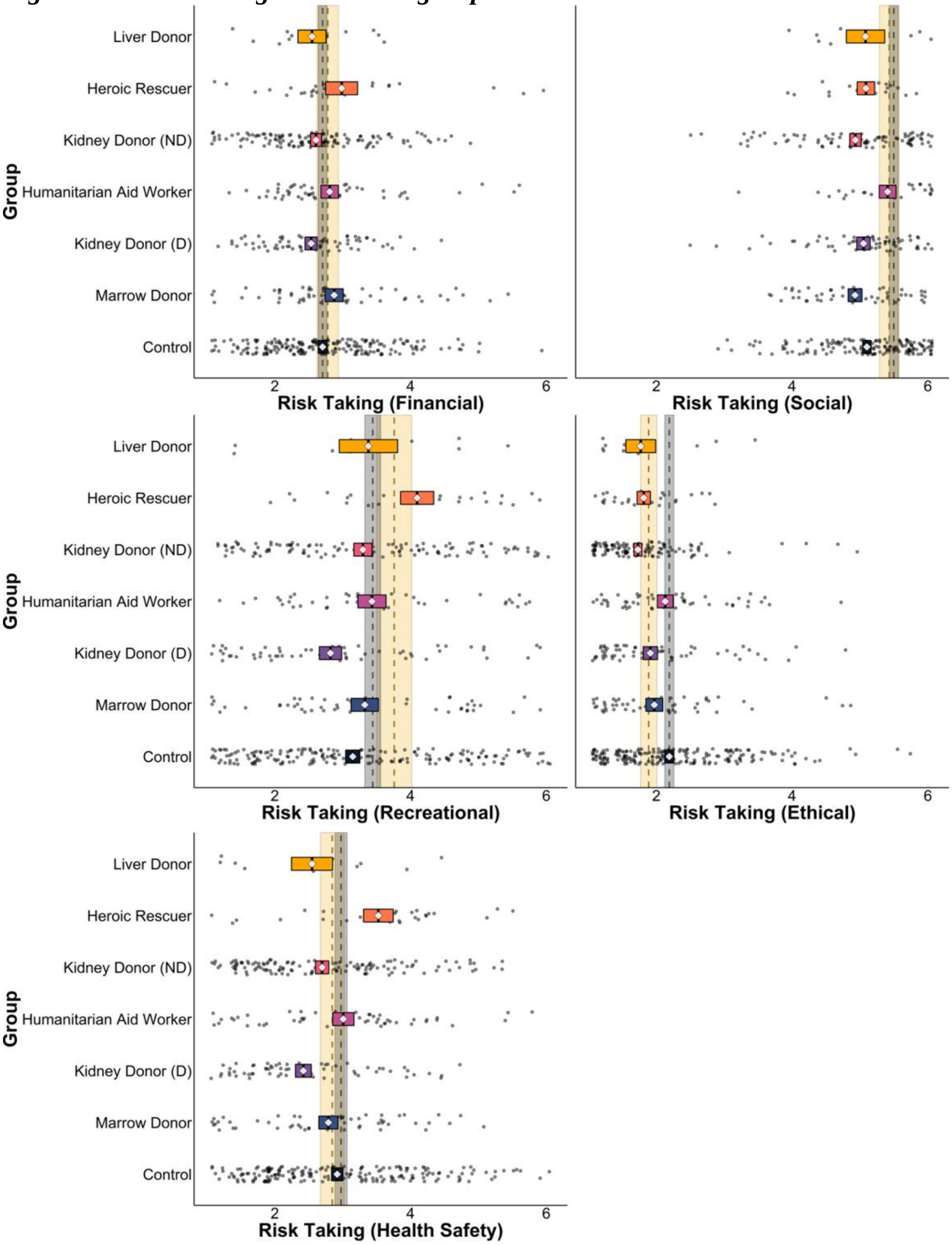

Note. Gray dashed line and ribbon represent control group mean and 95% CIs (N=207), yellow dashed line and ribbon represent combined altruist group mean and 95% CIs (N=347), diamonds represent means of individual groups, box widths represent 95% CIs. Multivariate regressions compared all altruistic groups against controls simultaneously using two-sided tests (in lieu of separate tests for which corrections for multiple comparisons would be appropriate).

Figure S1c. Risk taking perceptions across groups.

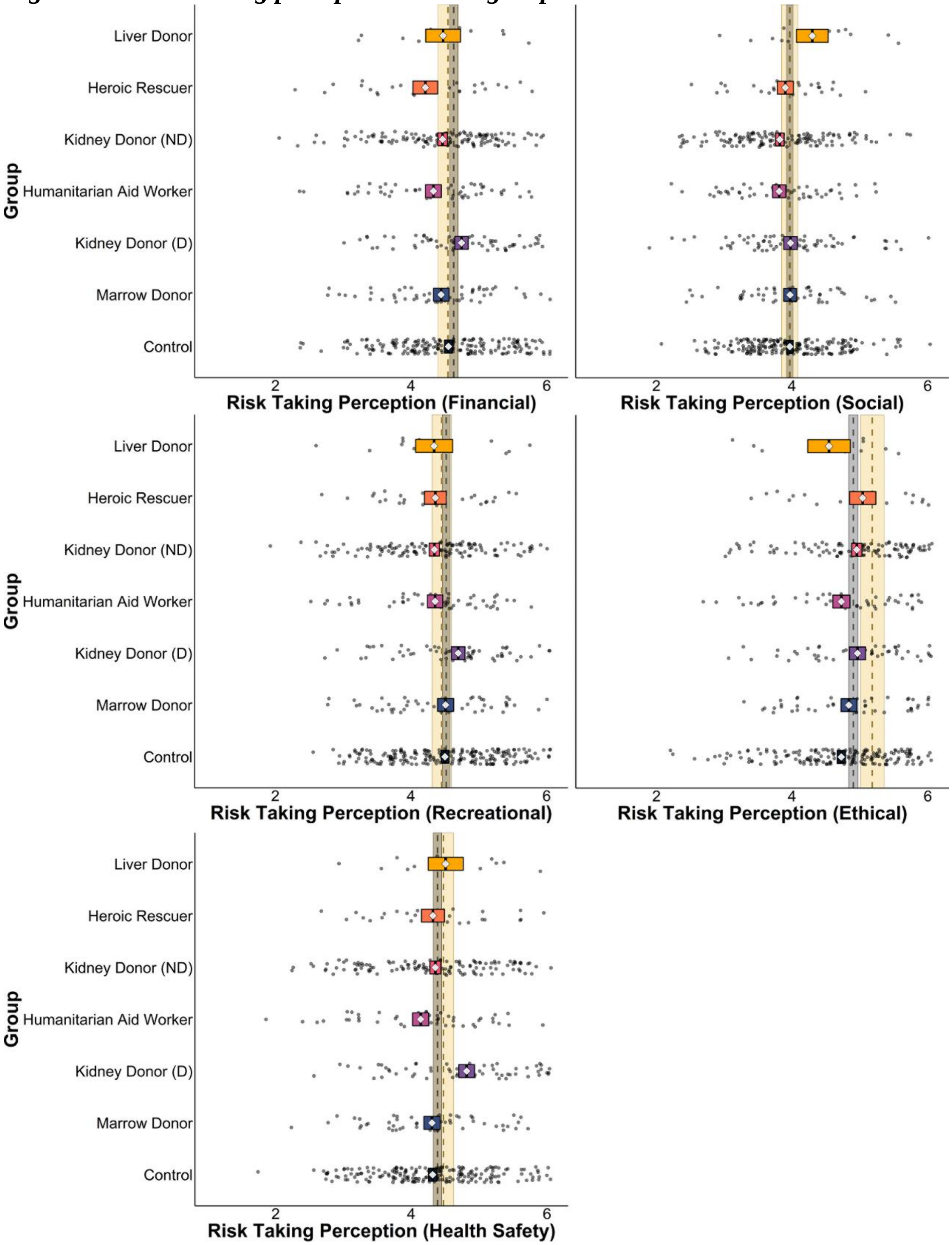

Note. Gray dashed line and ribbon represent control group mean and 95% CIs (N=207), yellow dashed line and ribbon represent combined altruist group mean and 95% CIs (N=347), diamonds represent means of individual groups, box widths represent 95% CIs. Multivariate regressions compared all altruistic groups against controls simultaneously using two-sided tests (in lieu of separate tests for which corrections for multiple comparisons would be appropriate).

Figure S1d. Psychopathic personality traits across groups.

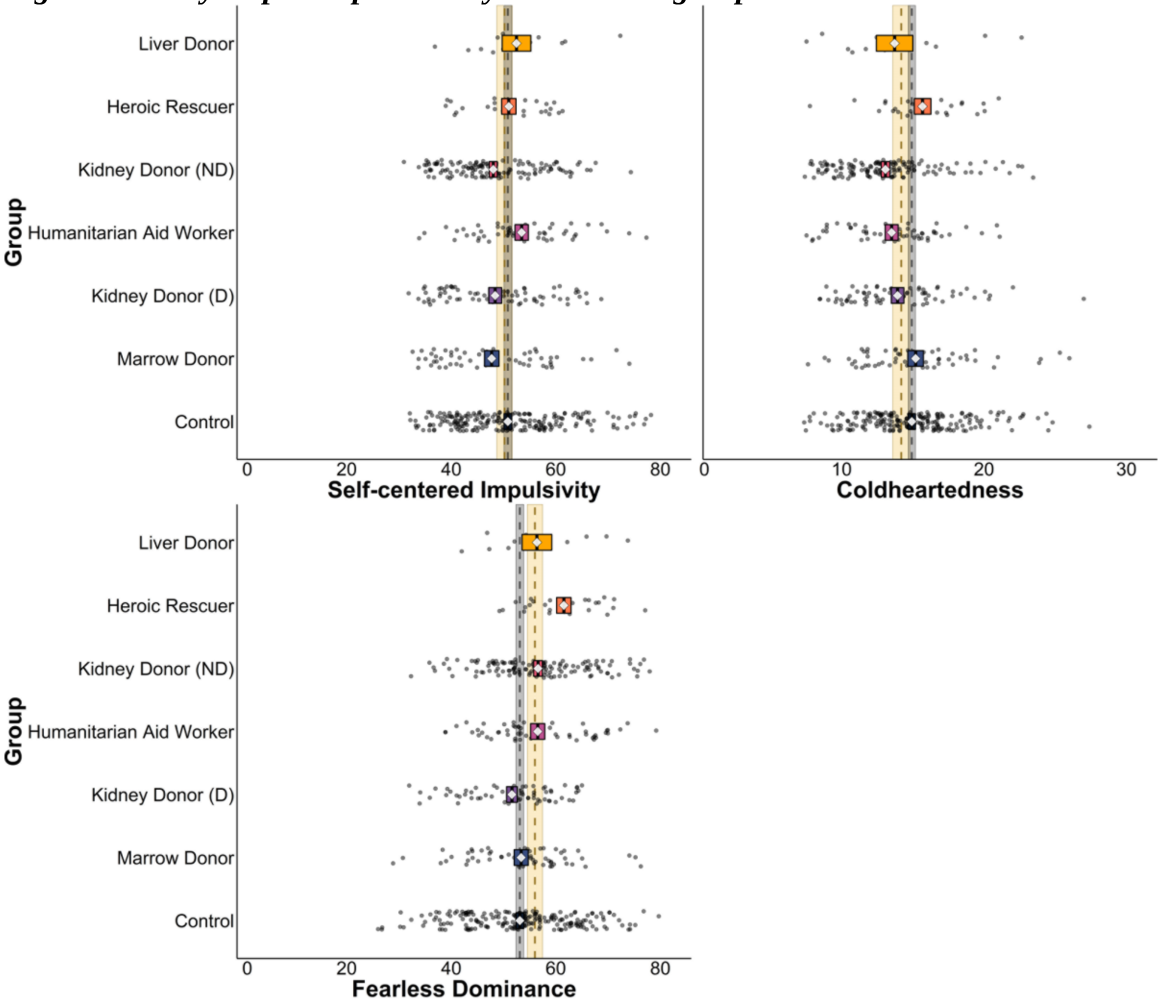

Note. Gray dashed line and ribbon represent control group mean and 95% CIs (N=207), yellow dashed line and ribbon represent combined altruist group mean and 95% CIs (N=347), diamonds represent means of individual groups, box widths represent 95% CIs. Multivariate regressions compared all altruistic groups against controls simultaneously using two-sided tests (in lieu of separate tests for which corrections for multiple comparisons would be appropriate).

Figure S2. Altruist and control samples compared to 5,000 bootstrap means drawn from Lee & Ashton (2020).

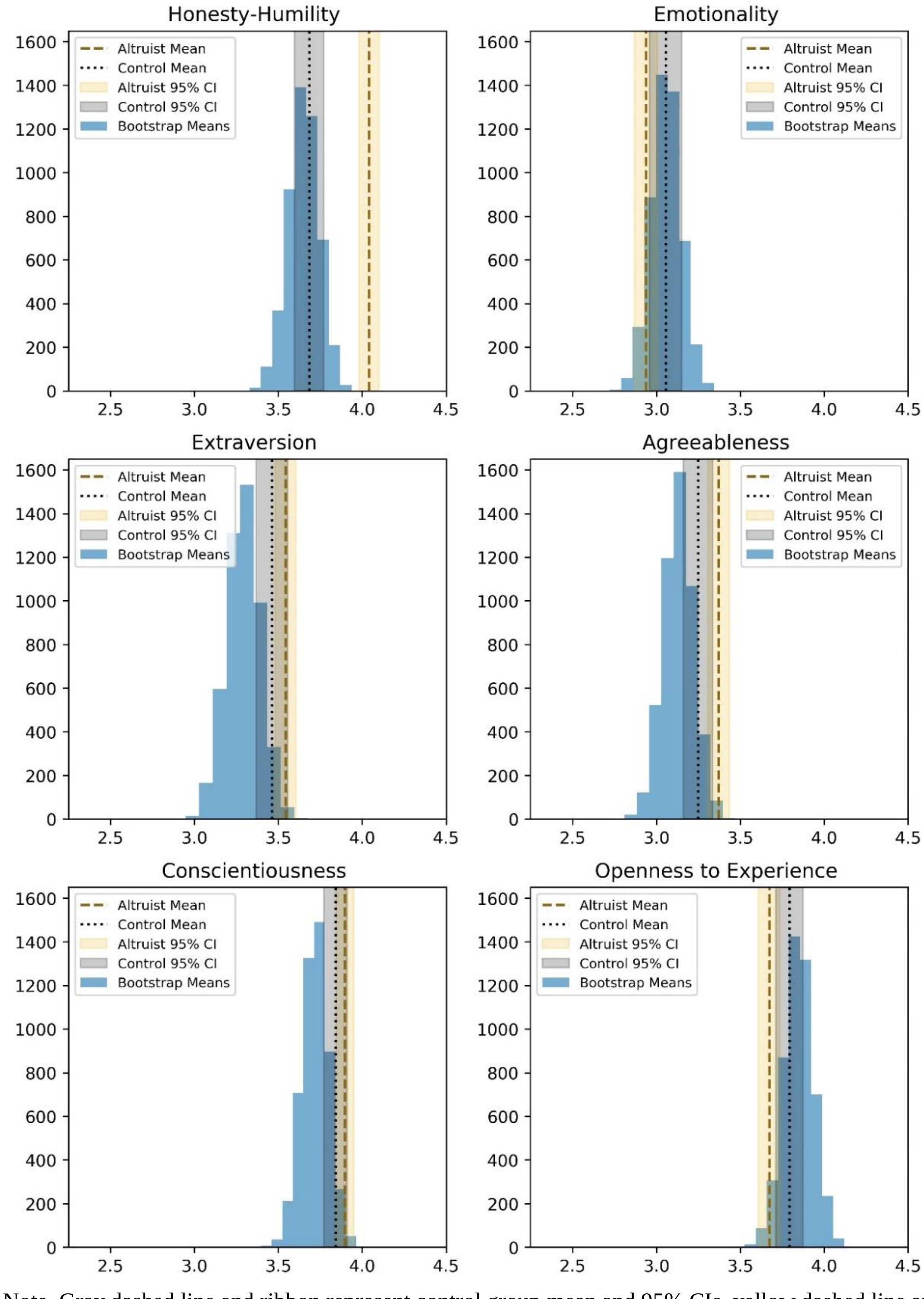

Note. Gray dashed line and ribbon represent control group mean and 95% CIs, yellow dashed line and ribbon represent combined altruist group mean and 95% CIs, histogram corresponds to the distribution of 5,000 bootstrap means (matched to the altruist group on age and sex). Means and SDs for altruists and controls are listed in Table S7. Honesty-Humility, $p<0.0002$; Emotionality, $p=0.1742$; Extraversion, $p=0.0106$; Agreeableness, $p=0.0096$; Conscientiousness, $p=0.0282$; Openness to Experience, $p=0.052$.

Figure S3. Area under the receiver-operator characteristic curve plot.

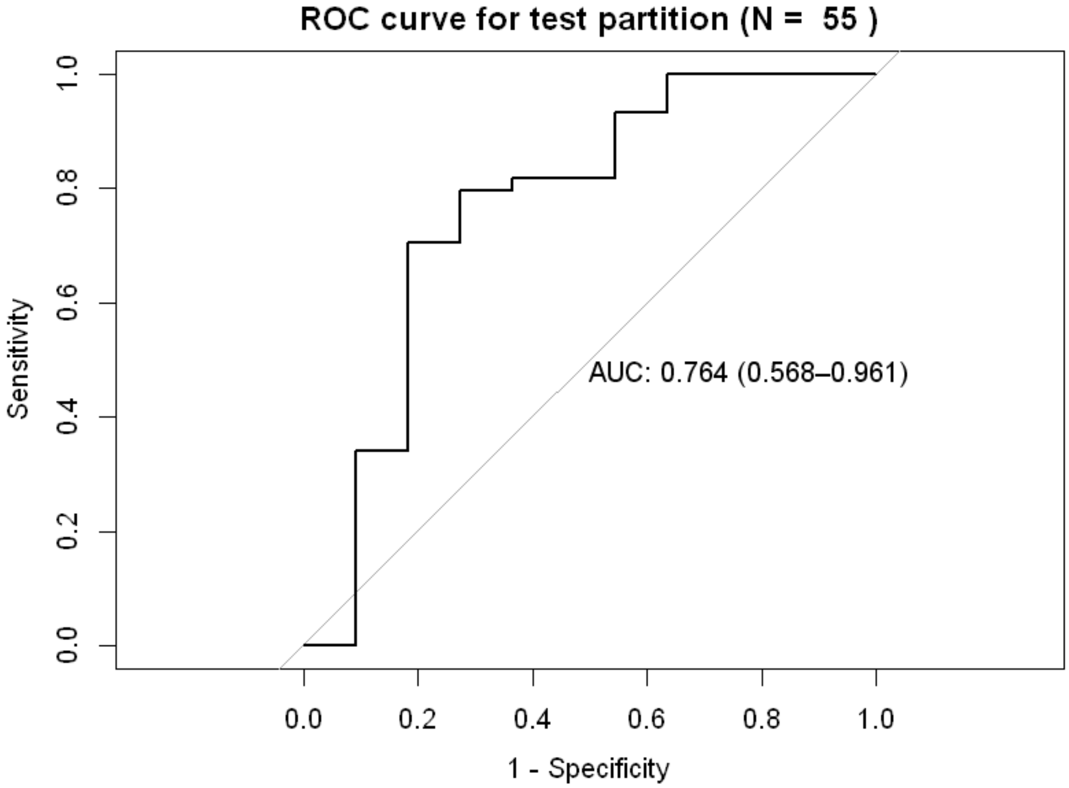

Note. This curve represents the classification accuracy of the logistic classifier with L1-regularization on the testing partition.

Figure S4a. Third-party ratings of altruism and risk.

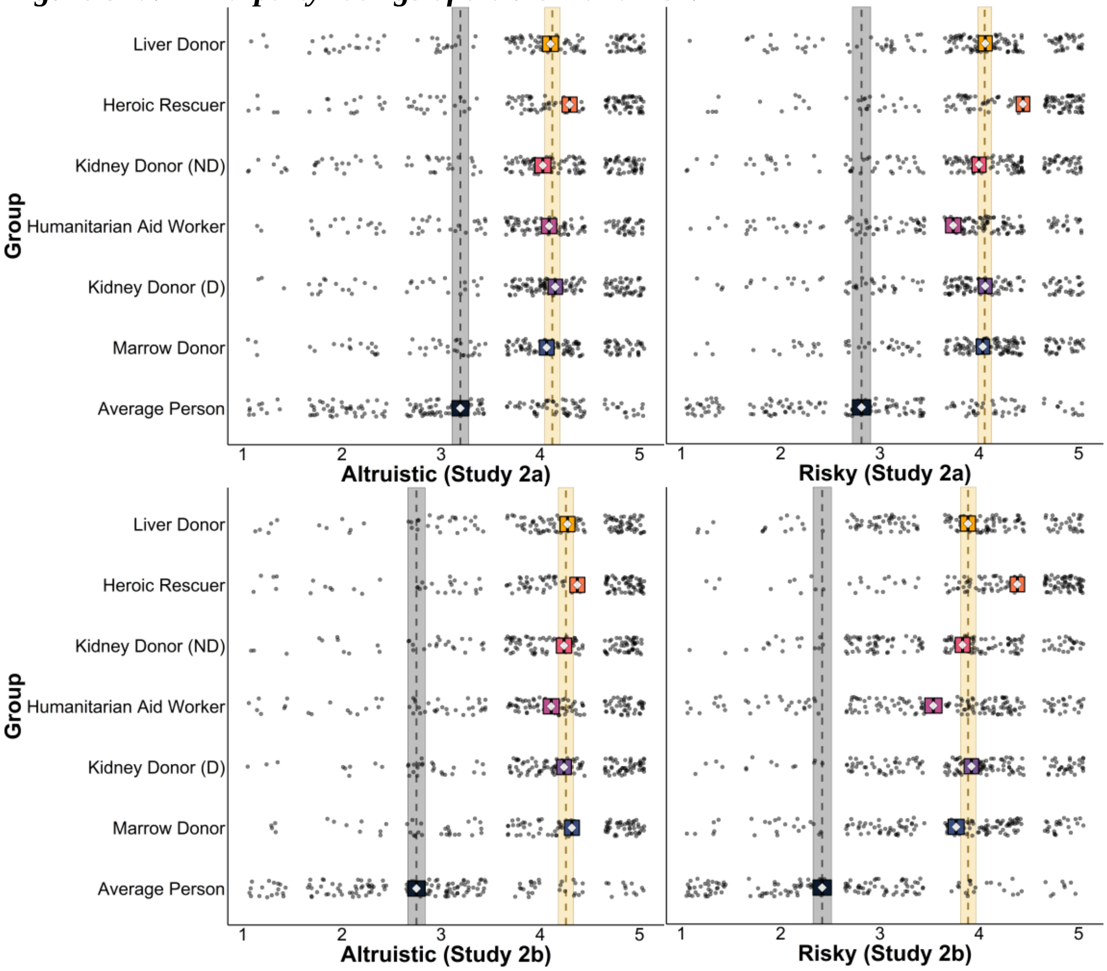

Note. Top plots correspond to the exploratory sample in Study 2a (N=208). Bottom plots correspond to the confirmatory sample in Study 2b (N=201). Gray dashed line and ribbon represent control group mean and 95% CIs, yellow dashed line and ribbon represent combined altruist group mean and 95% CIs, diamonds represent means of individual groups, box widths represent 95% CIs. Multivariate mixed-effects regressions compared all altruistic groups against controls simultaneously using two-sided tests (in lieu of separate tests for which corrections for multiple comparisons would be appropriate).

Figure S4b. Line plots estimated from third-party ratings of social discounting.

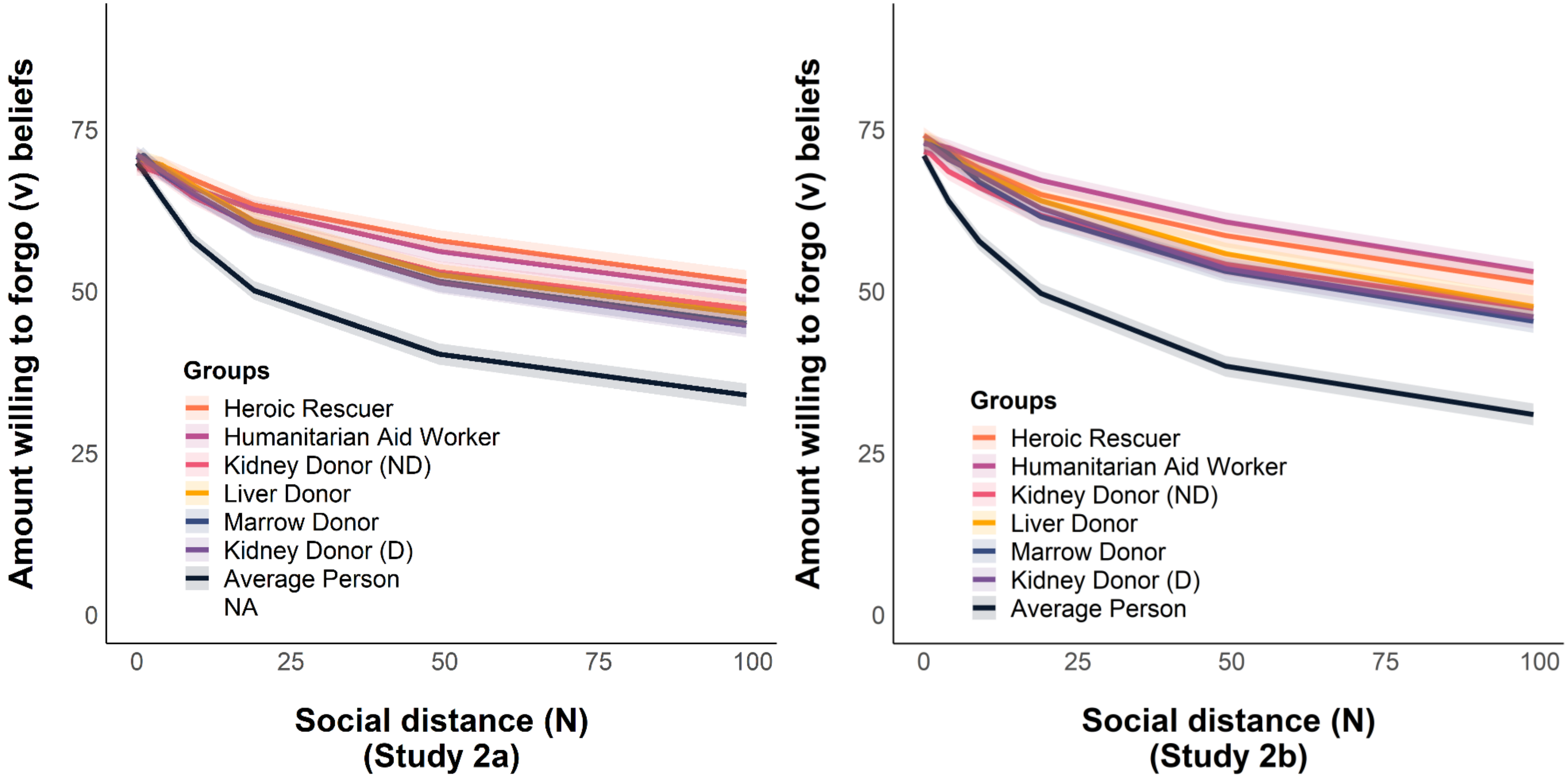

Note. Left plot corresponds to the exploratory sample in Study 2a (N=208). Right plot corresponds to the confirmatory sample in Study 2b (N=201). Labels in line plot are ordered in descending order of mean discounting slope across groups ($logk_{beliefs}$) in the Study 2a sample. Ribbons represent the 95% CIs. Multivariate mixed-effects regressions compared all altruistic groups against controls simultaneously using two-sided tests (in lieu of separate tests for which corrections for multiple comparisons would be appropriate).

Figure S4c. Third-party ratings of Honesty-Humility and Emotionality personality traits.

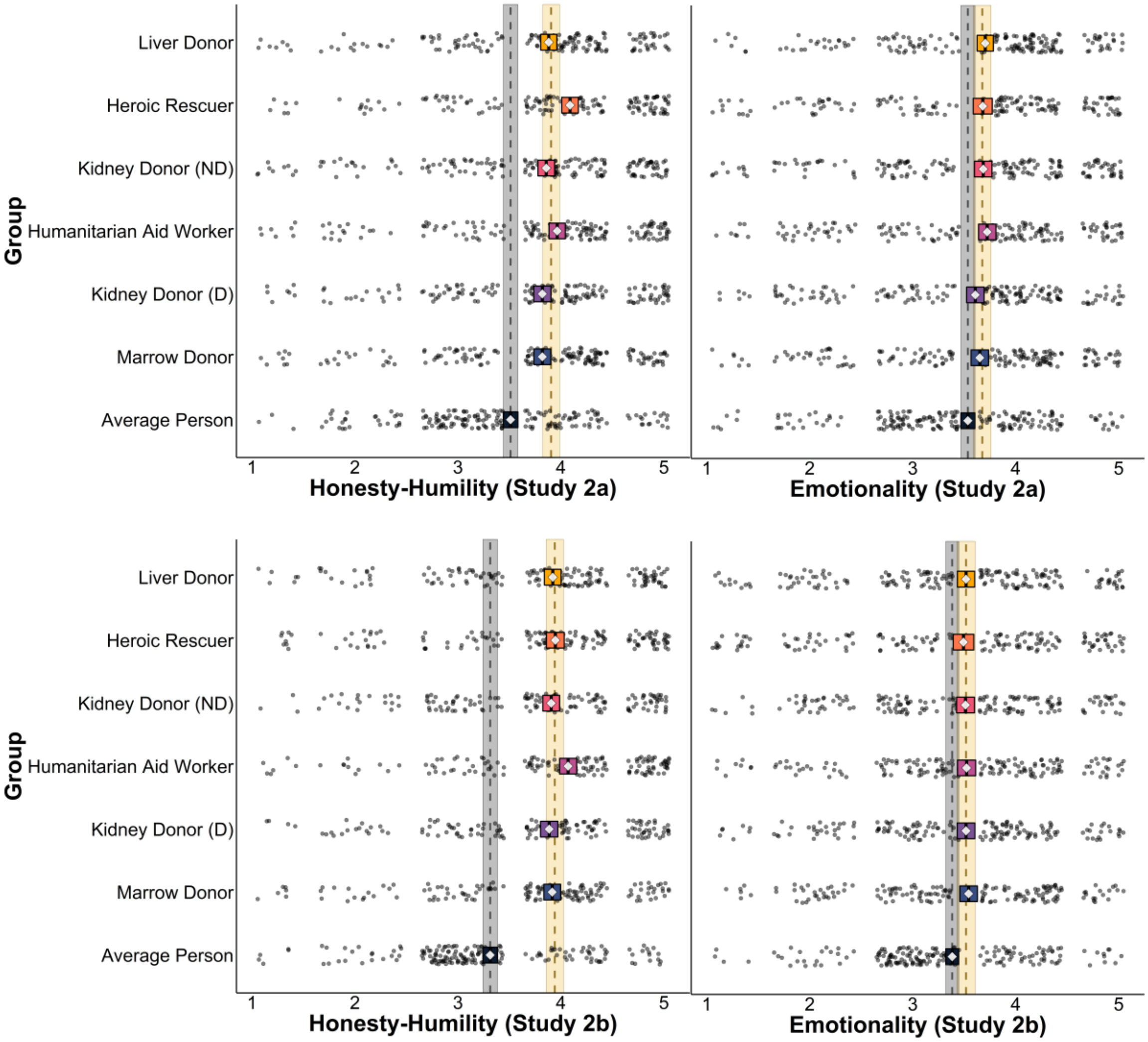

Note. Top plots correspond to the exploratory sample in Study 2a (N=208). Bottom plots correspond to the confirmatory sample in Study 2b (N=201). Gray dashed line and ribbon represent control group mean and 95% CIs, yellow dashed line and ribbon represent combined altruist group mean and 95% CIs, diamonds represent means of individual groups, box widths represent 95% CIs. Multivariate mixed-effects regressions compared all altruistic groups against controls simultaneously using two-sided tests (in lieu of separate tests for which corrections for multiple comparisons would be appropriate).

Figure S4d. Third-party ratings of Extraversion and Agreeableness personality traits.

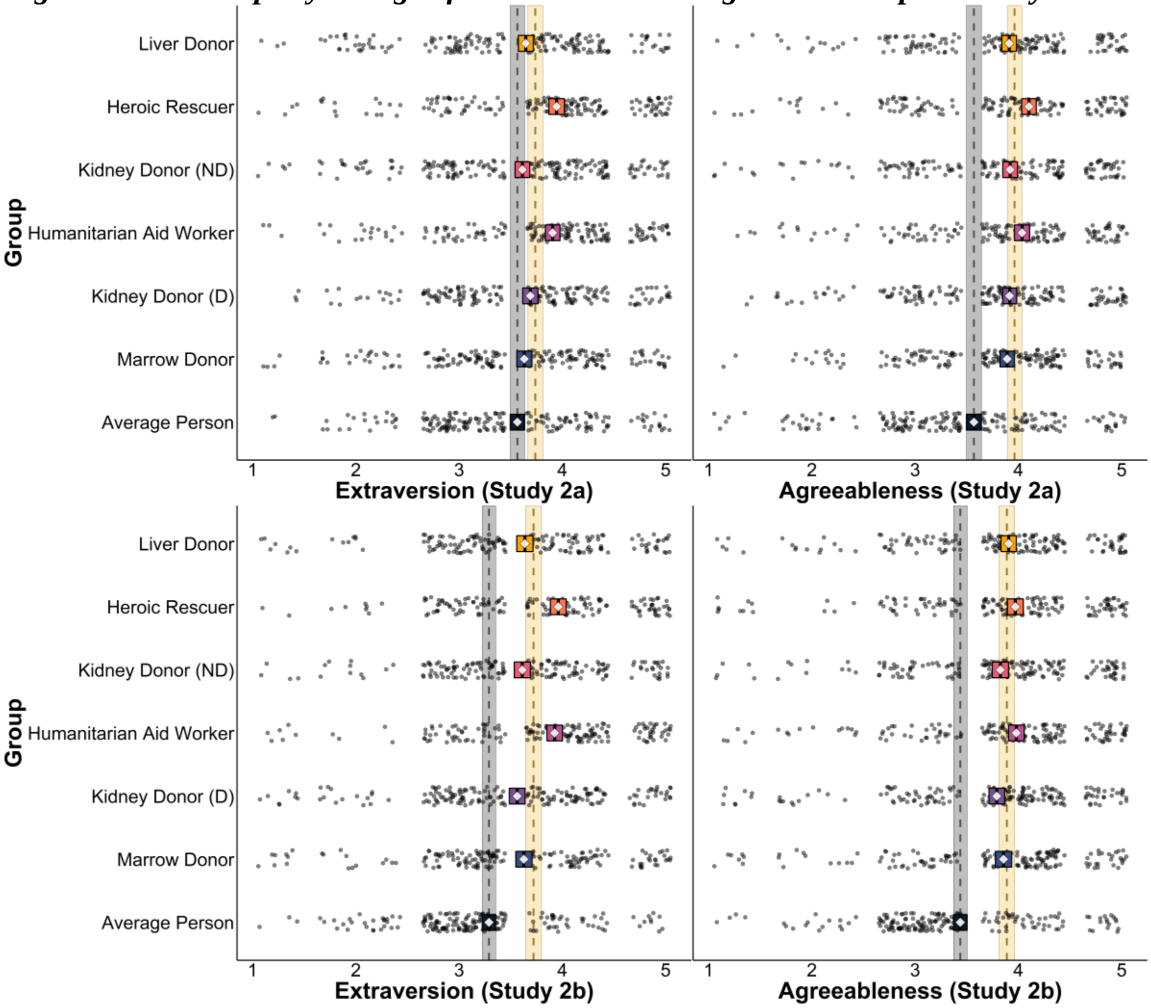

Note. Top plots correspond to the exploratory sample in Study 2a (N=208). Bottom plots correspond to the confirmatory sample in Study 2b (N=201). Gray dashed line and ribbon represent control group mean and 95% CIs, yellow dashed line and ribbon represent combined altruist group mean and 95% CIs, diamonds represent means of individual groups, box widths represent 95% CIs. Multivariate mixed-effects regressions compared all altruistic groups against controls simultaneously using two-sided tests (in lieu of separate tests for which corrections for multiple comparisons would be appropriate).

Figure S4e. Third-party ratings of Conscientiousness and Openness to Experience personality traits.

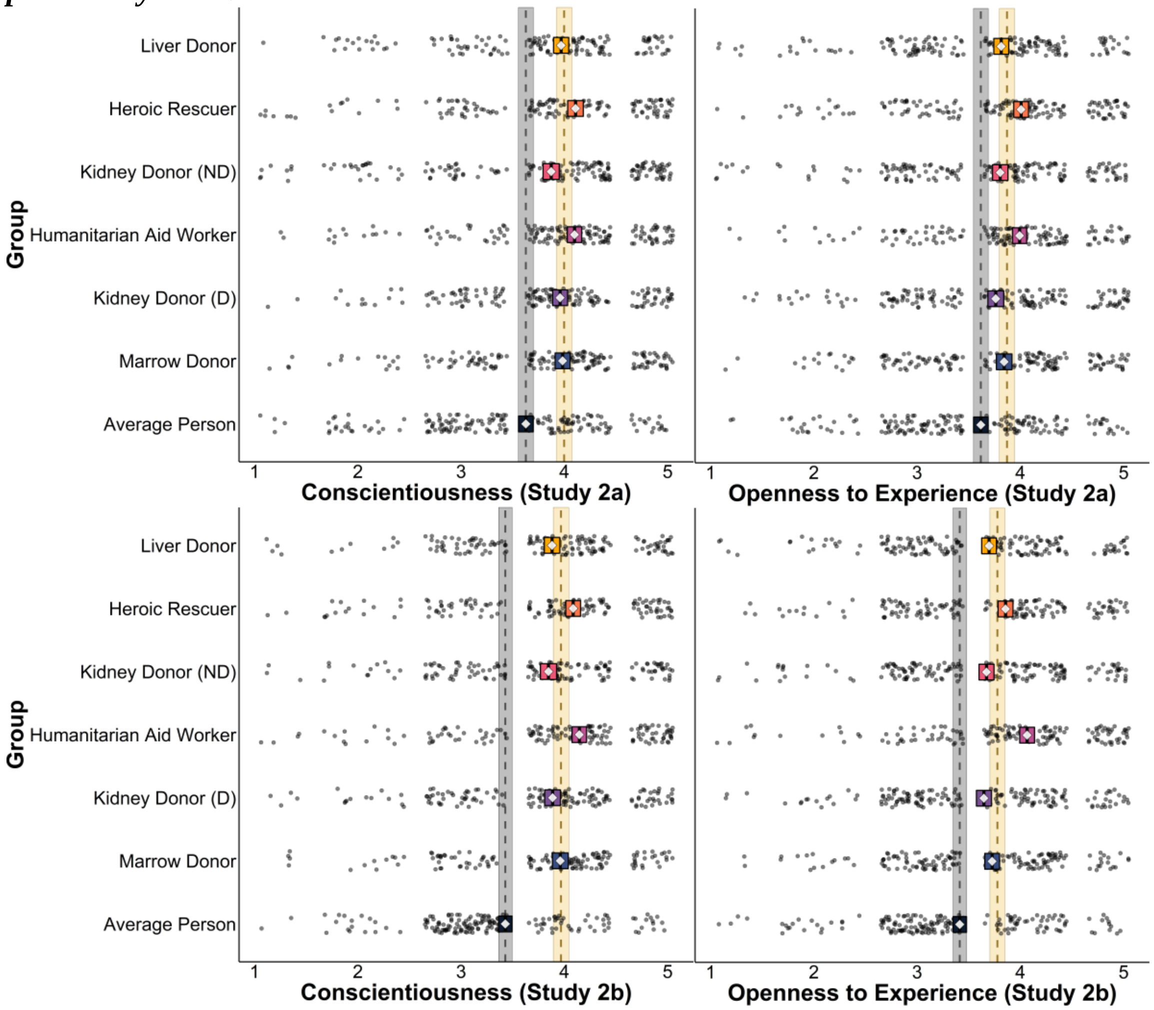

Note. Top plots correspond to the exploratory sample in Study 2a (N=208). Bottom plots correspond to the confirmatory sample in Study 2b (N=201). Gray dashed line and ribbon represent control group mean and 95% CIs, yellow dashed line and ribbon represent combined altruist group mean and 95% CIs, diamonds represent means of individual groups, box widths represent 95% CIs. Multivariate mixed-effects regressions compared all altruistic groups against controls simultaneously using two-sided tests (in lieu of separate tests for which corrections for multiple comparisons would be appropriate).

Figure S5a. BASE Jumper sample compared to 5,000 bootstrap means drawn from Lee & Ashton (2020).

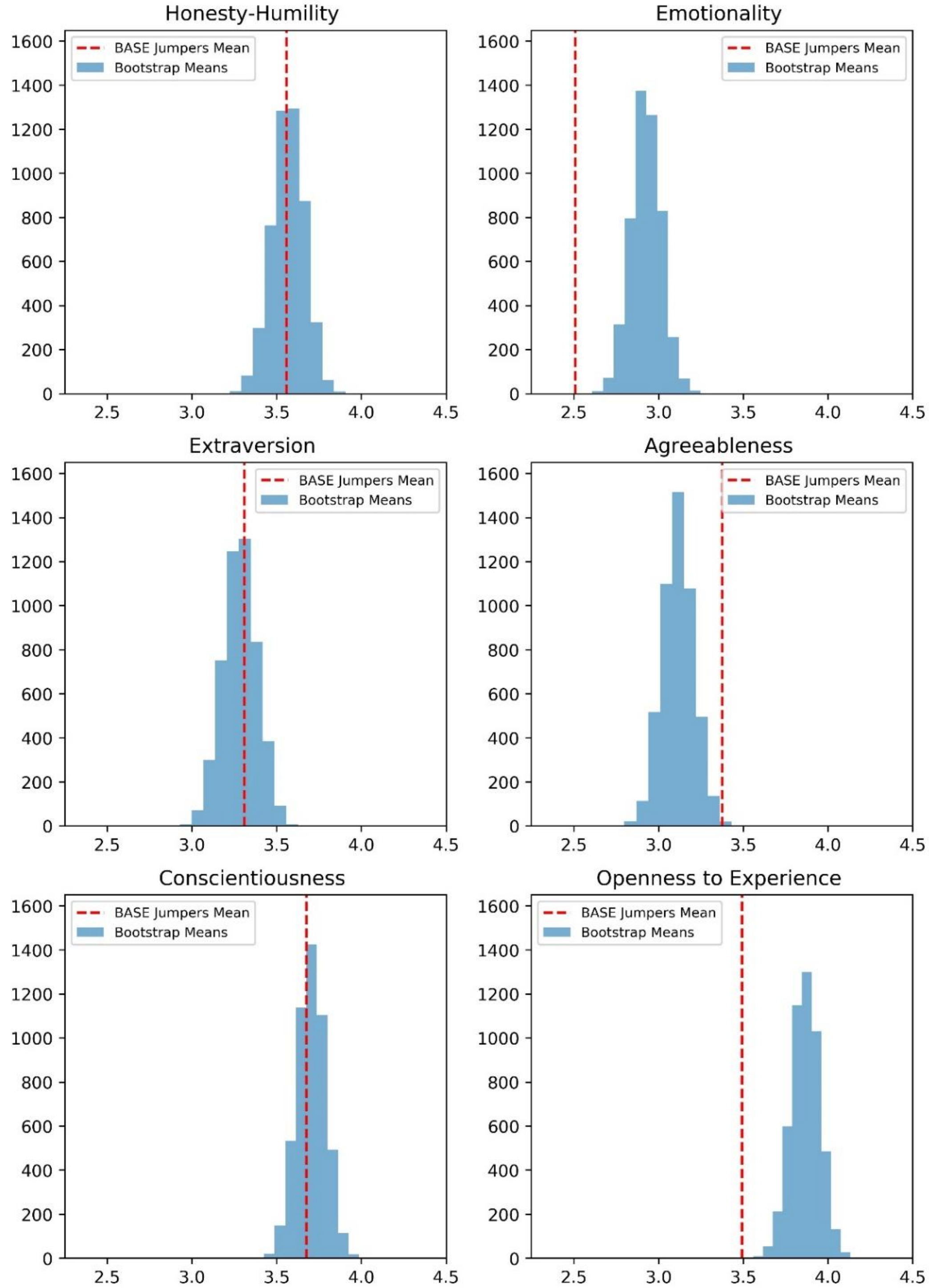

Note. Red dashed line represents BASE jumper group mean, histogram corresponds to the distribution of 5,000 bootstrap means (matched to the BASE jumper group on age and sex). Honesty-Humility, M=3.56, SE=0.22, p=0.9318; Emotionality, M= 2.51, SE=0.19, p<0.0002; Extraversion, M=3.31, SE=0.23, p=0.8068; Agreeableness, M=3.38, SE=0.16, p=0.006; Conscientiousness, M=3.68, SE=0.18, p=0.744; Openness to Experience, M=3.49, SE=0.15, p<0.0002.

Figure 5b. Miss America contestant sample compared to 5,000 bootstrap means drawn from Lee & Ashton (2020).

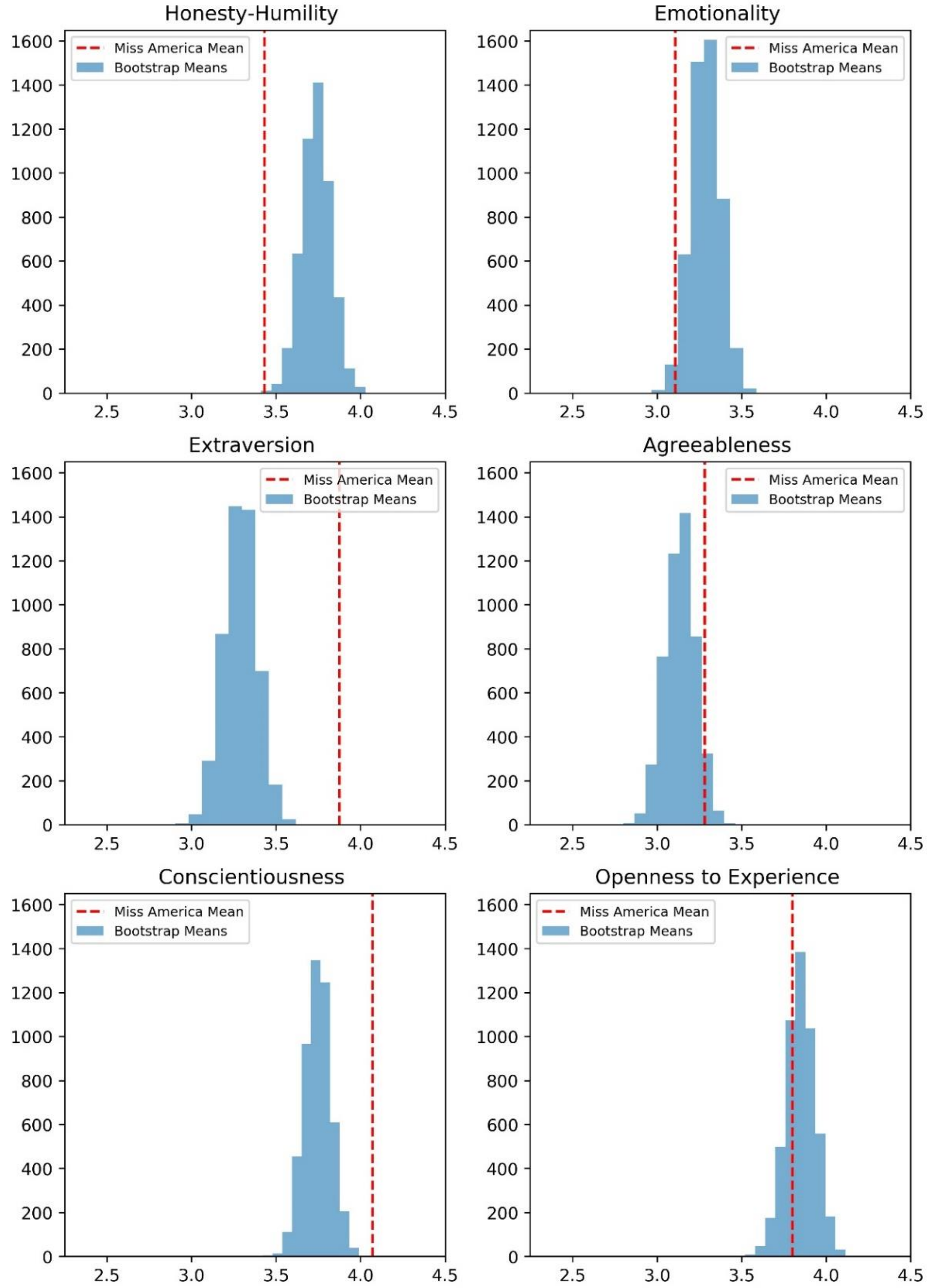

Note. Red dashed line represents Miss America contestant group mean, histogram corresponds to the distribution of 5,000 bootstrap means (matched to the Miss America contestant group on age and sex). Honesty-Humility, M=3.43, SE=0.18, p=0.0008; Emotionality, M=3.11, SE=0.13, p=0.0352; Extraversion, M=3.88, SE=0.18, p<0.0002; Agreeableness, M=3.28, SE=0.18, p=0.116; Conscientiousness, M=4.07, SE=0.12, p<0.0002; Openness to Experience, M=3.8, SE=0.15, p=0.59. P-values are two-tailed.

Figure S6. Representational similarity among trait perceptions and actual traits of altruists.

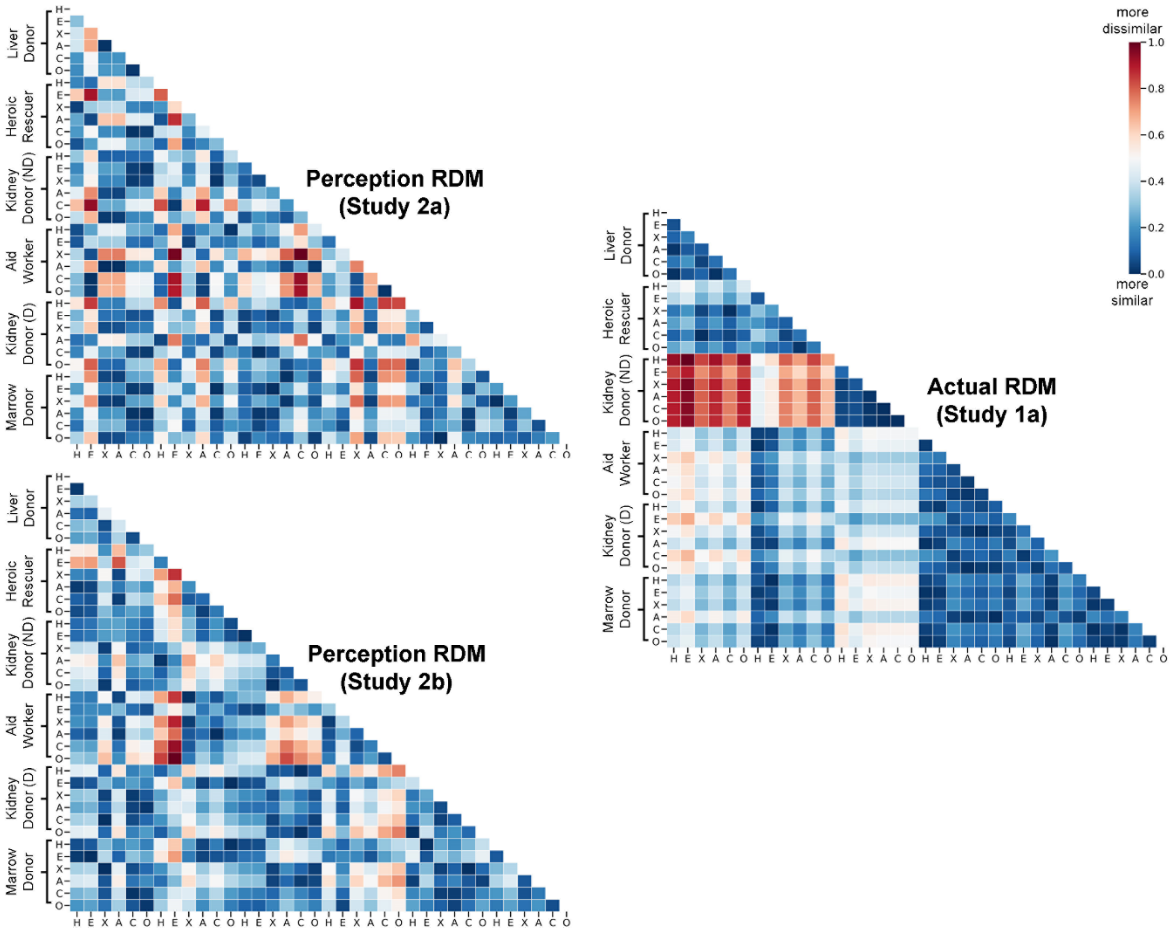

Note. Cells in each representational dissimilarity matrix represent the degree of similarity between group perceptions (left) or actual group characteristics (right), measured using absolute differences. These differences were scaled between 0 (most similar) to 1 (least similar) for visualization purposes. The Spearman rho was pairwise computed to assess correspondence across matrices and statistical inference was conducted using the Mantel Test. (H=Honesty-Humility, E=Emotionality, X=Extraversion, A=Agreeableness, C=Conscientiousness, O=Openness to Experience).

Figure S7abc. Non-parametric statistical inference of representational similarity.

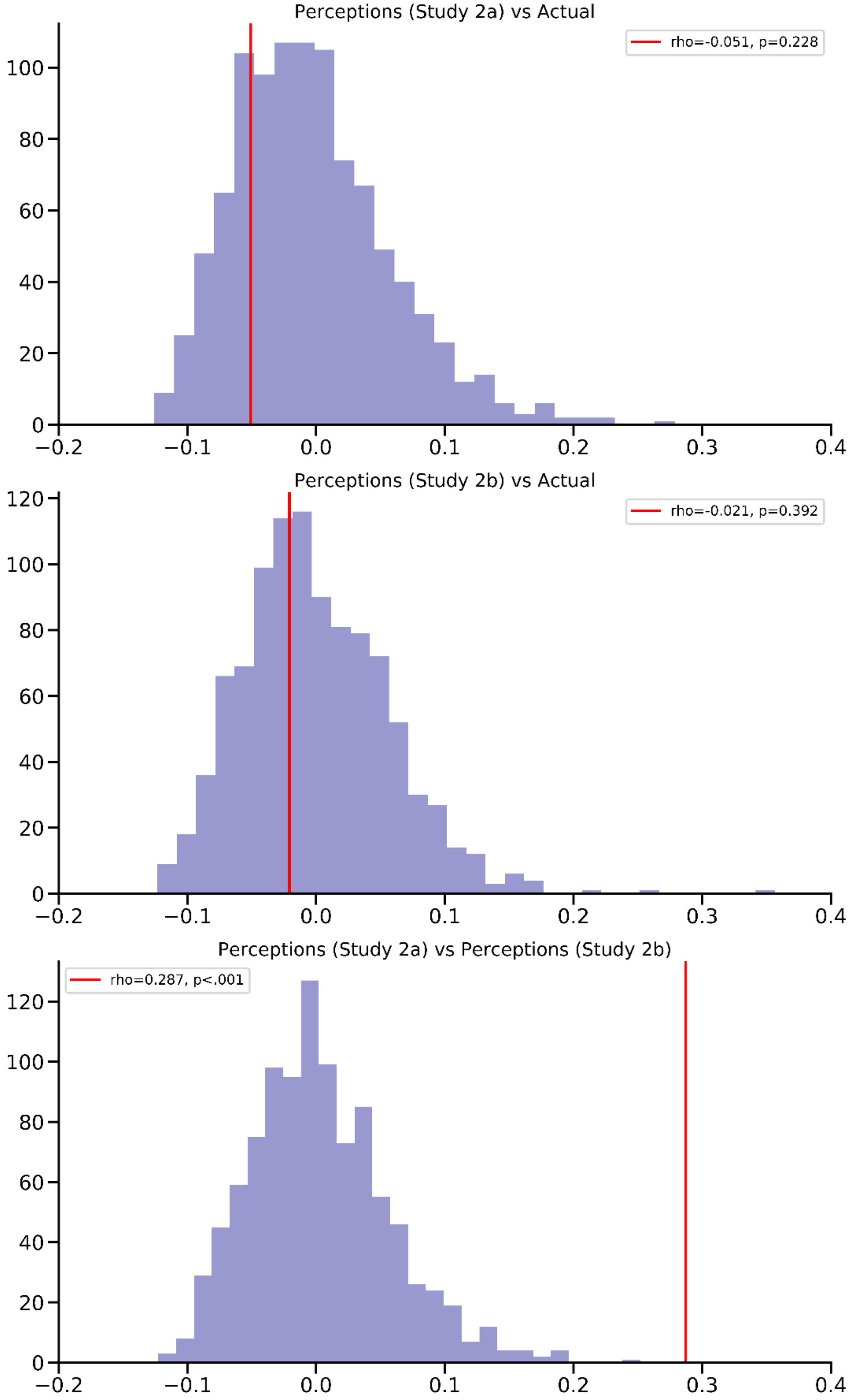

Note. Mantel permutation test fails to reject the null hypothesis that the correspondence between the similarity structure of perceptions and actual traits is due to chance, but finds robust similarity between perceptions of altruists across the two representative samples. P-values are two-tailed.

Figure S8. Relationship between AUC and logk.

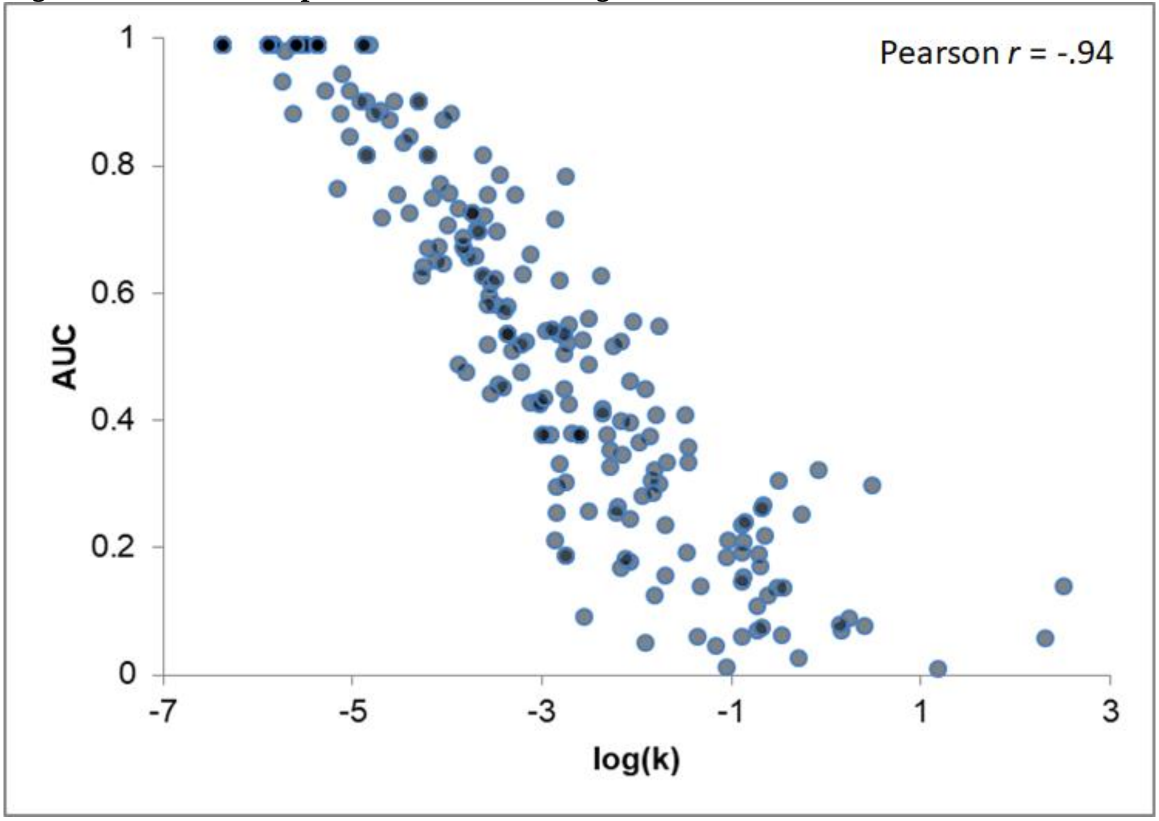

Note. Scatterplot shows the relationship between the model agnostic measures of area under the curve and the logk estimates from the hyperbolic model, demonstrating a strong inverse relationship.

Table S1. Per capita prevalence estimates and demographics for each group among Americans in 2017

**U.S. total population**[a] **= 324,985,539**	**Total number**	**Per capita prevalence**	**Sex F : M**	**Age range**
Marrow Donations[b]	4,972	0.00001530%	2,086 : 2,886	0 - 70+
Kidney Donations (Directed)[c]	5,554	0.00001709%	3,507: 2,047	18-65+
Humanitarian Aid Work[d]	3,664	0.00001127%	N/A	N/A
Kidney Donations (Non-directed)[c]	258	0.00000079%	152: 106	18-64
Heroic Rescue Awards[e]	77	0.00000024%	8 : 69	10-76
Liver Donations (Directed & Non-directed)[c]	367	0.00000113%	201 : 166	11-65+

[a] U.S. Census Bureau. (2017). U.S. and World Population. Retrieved from http://www.census.gov/popclock

[b] Center for International Blood and Marrow Transplant, a contractor for the C.W. Bill Young Cell Transplantation Program operated through the U. S. Department of Health and Human Services, Health Resources and Services Administration, H. S. B. (2017). U.S. Transplant Data, Number of Transplants by Year by Donor Type Report. Retrieved from https://bloodcell.transplant.hrsa.gov/research/transplant_data/transplant_activity_report/index.html

[c] Organ Procurement and Transplantation Network. (2017). Living Donor Transplants By Donor Relation. Retrieved from https://optn.transplant.hrsa.gov/data/view-data-reports/national-data

[d] Médicins Sans Frontiere. (2017). International Activity Report. Retrieved from https://www.msf.org/international-activity-report-2017

[e] Carnegie Hero Fund Commission. (2017). Carnegie Hero Awardees. Retrieved from https://www.carnegiehero.org

Table S2. Table of demographics of personality traits full-sample (N=554)

		Control	Combined Altruists	Bone Marrow Donor	Kidney Donor (D)	Humanitarian Aid Worker	Kidney Donor (ND)	Heroic Rescuer	Liver Donor
N = 554		207	347	55	68	53	132	27	12
Sex	F : M : Other	134 : 73	211 : 135 : 1	26 : 29	54 : 13 : 1	36 : 17	86 : 46	1 : 26	8 : 4
Age	M (SE)	37.71 (.63)	44.1 (.67)	36.18 (1.51)	46.13 (1.56)	41.49 (1.62)	46.53 (1.02)	50.22 (2.41)	38.83 (2.34)
Household Income									
Under $9,999		11	8	4	2	-	2	-	-
$10,000-14,999		5	5	-	-	1	4	-	-
$15,000-24,999		5	10	5	-	3	1	-	1
$25,000-39,999		16	26	3	5	3	13	2	-
$40,000-59,999		25	43	7	7	11	15	2	1
$60,000-89,999		43	57	8	13	10	18	5	3
$90,000-179,999		62	125	22	32	14	44	7	6
Over $180,000		27	65	4	8	10	33	10	-
Don't know / No response		13	8	2	1	1	2	1	1
Race									
White		149	291	29	64	40	123	25	10
African American / Black		29	12	7	1	1	1	1	1
Asian / Pacific Islander		13	14	5	1	3	5	-	-
Latino		3	8	5	1	1	-	-	1
Native American/Alaskan		1	2	2	-	-	-	-	-
Multiracial		7	7	4	-	1	1	1	-
Other		5	12	3	1	6	2	-	-
Prefer not to Respond		-	1	-	-	1	-	-	-
Hispanic									
Yes		3	8	5	1	1	-	-	1
No		204	339	50	67	52	132	27	11
Education									
High School		10	17	2	4	-	8	2	1
Some College		15	75	11	14	6	28	11	5
Bachelor's Degree		87	126	27	29	13	49	7	1
Master's Degree		72	92	6	18	26	32	5	5
Professional/Doctoral Degree		23	37	9	3	8	15	2	-
Prefer not to Respond		-	-	-	-	-	-	-	-
Religious									
Yes		65	150	36	39	11	45	16	3
No		141	197	19	29	42	87	11	9
No response		1	-	-	-	-	-	-	-

Table S3a-i. Regression results for Honesty-Humility comparing altruistic groups to controls (N=553)

Honesty-Humility	Estimate	SE	95% CI	T	p
Intercept	3.639 ***	0.054	[3.533, 3.745]	67.516	< .0001
Marrow Donor	0.293 ***	0.089	[0.119, 0.467]	3.313	0.001
Kidney Donor (D)	0.261 **	0.084	[0.096, 0.425]	3.115	0.002
Humanitarian Aid Worker	0.202 *	0.090	[0.026, 0.378]	2.252	0.025
Kidney Donor (ND)	0.334 ***	0.068	[0.201, 0.467]	4.929	< .0001
Heroic Rescuer	0.309 *	0.126	[0.061, 0.557]	2.445	0.015
Liver Donor	0.224	0.172	[-0.115, 0.562]	1.299	0.194
Age	0.012 ***	0.002	[0.007, 0.016]	5.062	< .0001
Sex (Female)	0.142 **	0.054	[0.037, 0.248]	2.659	0.008

Note. F(8, 544) = 10.985; R^2 = 0.139; Groups are coded as indicator variables relative to the control group. Coefficients are unstandardized. SE indicates the standard error. 95% CI indicates lower/upper limits of the confidence interval. Intercept corresponds to the mean for controls who are not female at average age. * indicates p<.05, ** indicates p<.01, *** indicates p<.001. P-values are two-tailed. Multivariate regressions compared all altruistic groups against controls simultaneously (in lieu of separate tests for which corrections for multiple comparisons would be appropriate).

Table S3a-ii. Regression results for Honesty-Humility comparing altruistic groups to controls (N=532)

Honesty-Humility	Estimate	SE	95% CI	T	p
Intercept	3.667 ***	0.083	[3.505, 3.829]	44.403	< .0001
Marrow Donor	0.277 **	0.092	[0.096, 0.457]	3.010	0.003
Kidney Donor (D)	0.263 **	0.086	[0.093, 0.432]	3.044	0.002
Humanitarian Aid Worker	0.194 *	0.092	[0.013, 0.375]	2.110	0.035
Kidney Donor (ND)	0.329 ***	0.070	[0.191, 0.467]	4.695	< .0001
Heroic Rescuer	0.293 *	0.132	[0.033, 0.553]	2.213	0.027
Liver Donor	0.264	0.183	[-0.095, 0.624]	1.444	0.149
Age	0.012 ***	0.002	[0.007, 0.017]	4.792	< .0001
Sex (Female)	0.142 *	0.055	[0.033, 0.250]	2.565	0.011
College Education	-0.006	0.067	[-0.137, 0.124]	-0.096	0.924
Income (≥$90k)	-0.04	0.054	[-0.146, 0.066]	-0.734	0.463

Note. F(10, 521) = 8.063; R^2 = 0.134; Groups are coded as indicator variables relative to the control group. Coefficients are unstandardized. SE indicates the standard error. 95% CI indicates lower/upper limits of the confidence interval. Intercept corresponds to the mean for controls who are not female at average age who never completed a four-year degree and household earns <$90,000. * indicates p<.05, ** indicates p<.01, *** indicates p<.001. P-values are two-tailed. Multivariate regressions compared all altruistic groups against controls simultaneously (in lieu of separate tests for which corrections for multiple comparisons would be appropriate).

Table S3b-i. Regression results for Emotionality comparing altruistic groups to controls (N=553)

Emotionality	Estimate	SE	95% CI	T	p
Intercept	2.692 ***	0.056	[2.581, 2.803]	47.702	< .0001
Marrow Donor	-0.05	0.093	[-0.232, 0.133]	-0.534	0.593
Kidney Donor (D)	0.052	0.088	[-0.120, 0.224]	0.596	0.552
Humanitarian Aid Worker	-0.137	0.094	[-0.322, 0.047]	-1.462	0.144
Kidney Donor (ND)	-0.108	0.071	[-0.247, 0.031]	-1.523	0.128
Heroic Rescuer	-0.252	0.132	[-0.512, 0.007]	-1.910	0.057
Liver Donor	-0.098	0.180	[-0.452, 0.256]	-0.544	0.587
Age	-0.002	0.002	[-0.007, 0.002]	-0.939	0.348
Sex (Female)	0.549 ***	0.056	[0.439, 0.660]	9.797	< .0001

Note. F(8, 544) = 16.277; R^2 = 0.193; Groups are coded as indicator variables relative to the control group. Coefficients are unstandardized. SE indicates the standard error. 95% CI indicates lower/upper limits of the confidence interval. Intercept corresponds to the mean for controls who are not female at average age. * indicates p<.05, ** indicates p<.01, *** indicates p<.001. P-values are two-tailed. Multivariate regressions compared all altruistic groups against controls simultaneously (in lieu of separate tests for which corrections for multiple comparisons would be appropriate).

Table S3b-ii. Regression results for Emotionality comparing altruistic groups to controls (N=532)

Emotionality	Estimate	SE	95% CI	T	p
Intercept	2.637 ***	0.086	[2.467, 2.806]	30.589	< .0001
Marrow Donor	-0.044	0.096	[-0.233, 0.144]	-0.463	0.643
Kidney Donor (D)	0.062	0.090	[-0.115, 0.239]	0.689	0.491
Humanitarian Aid Worker	-0.14	0.096	[-0.329, 0.049]	-1.458	0.146
Kidney Donor (ND)	-0.109	0.073	[-0.253, 0.035]	-1.492	0.136
Heroic Rescuer	-0.22	0.138	[-0.491, 0.051]	-1.595	0.111
Liver Donor	-0.097	0.191	[-0.472, 0.278]	-0.508	0.612
Age	-0.002	0.003	[-0.007, 0.004]	-0.592	0.554
Sex (Female)	0.558 ***	0.058	[0.445, 0.671]	9.685	< .0001
College Education	0.086	0.070	[-0.051, 0.222]	1.233	0.218
Income (≥$90k)	-0.046	0.056	[-0.157, 0.065]	-0.815	0.415

Note. F(10, 521) = 12.842; R^2 = 0.198; Groups are coded as indicator variables relative to the control group. Coefficients are unstandardized. SE indicates the standard error. 95% CI indicates lower/upper limits of the confidence interval. Intercept corresponds to the mean for controls who are not female at average age who never completed a four-year degree and household earns <$90,000. * indicates p<.05, ** indicates p<.01, *** indicates p<.001. P-values are two-tailed. Multivariate regressions compared all altruistic groups against controls simultaneously (in lieu of separate tests for which corrections for multiple comparisons would be appropriate).

Table S3c-i. Regression results for Extraversion comparing altruistic groups to controls (N=553)

Extraversion	Estimate	SE	95% CI	T	p
Intercept	3.491 ***	0.057	[3.378, 3.604]	60.761	< .0001
Marrow Donor	-0.049	0.094	[-0.235, 0.136]	-0.524	0.601
Kidney Donor (D)	-0.049	0.089	[-0.224, 0.127]	-0.547	0.585
Humanitarian Aid Worker	0.146	0.096	[-0.041, 0.334]	1.531	0.126
Kidney Donor (ND)	0.121	0.072	[-0.020, 0.263]	1.681	0.093
Heroic Rescuer	-0.084	0.135	[-0.349, 0.180]	-0.627	0.531
Liver Donor	-0.187	0.184	[-0.547, 0.174]	-1.017	0.31
Age	0.007 **	0.002	[0.002, 0.011]	2.722	0.007
Sex (Female)	-0.003	0.057	[-0.115, 0.109]	-0.055	0.956

Note. F(8, 544) = 2.567; R^2 = 0.036; Groups are coded as indicator variables relative to the control group. Coefficients are unstandardized. SE indicates the standard error. 95% CI indicates lower/upper limits of the confidence interval. Intercept corresponds to the mean for controls who are not female at average age. * indicates p<.05, ** indicates p<.01, *** indicates p<.001. P-values are two-tailed. Multivariate regressions compared all altruistic groups against controls simultaneously (in lieu of separate tests for which corrections for multiple comparisons would be appropriate).

Table S3c-ii. Regression results for Extraversion comparing altruistic groups to controls (N=532)

Extraversion	Estimate	SE	95% CI	T	p
Intercept	3.474 ***	0.087	[3.304, 3.644]	40.116	< .0001
Marrow Donor	-0.101	0.096	[-0.290, 0.089]	-1.045	0.297
Kidney Donor (D)	-0.068	0.090	[-0.246, 0.110]	-0.751	0.453
Humanitarian Aid Worker	0.134	0.097	[-0.055, 0.324]	1.392	0.164
Kidney Donor (ND)	0.094	0.074	[-0.051, 0.238]	1.277	0.202
Heroic Rescuer	-0.114	0.139	[-0.386, 0.159]	-0.821	0.412
Liver Donor	-0.315	0.192	[-0.692, 0.062]	-1.641	0.101
Age	0.006 *	0.003	[0.001, 0.011]	2.479	0.013
Sex (Female)	0.012	0.058	[-0.102, 0.125]	0.201	0.84
College Education	-0.024	0.070	[-0.161, 0.114]	-0.338	0.735
Income (≥$90k)	0.1	0.057	[-0.011, 0.211]	1.766	0.078

Note. F(10, 521) = 2.697; R^2 = 0.049; Groups are coded as indicator variables relative to the control group. Coefficients are unstandardized. SE indicates the standard error. 95% CI indicates lower/upper limits of the confidence interval. Intercept corresponds to the mean for controls who are not female at average age who never completed a four-year degree and household earns <$90,000. * indicates p<.05, ** indicates p<.01, *** indicates p<.001. P-values are two-tailed. Multivariate regressions compared all altruistic groups against controls simultaneously (in lieu of separate tests for which corrections for multiple comparisons would be appropriate).

Table S3d-i. Regression results for Agreeableness comparing altruistic groups to controls (N=553)

Agreeableness	Estimate	SE	95% CI	T	p
Intercept	3.351 ***	0.056	[3.241, 3.462]	59.527	< .0001
Marrow Donor	0.15	0.092	[-0.032, 0.332]	1.621	0.106
Kidney Donor (D)	-0.017	0.087	[-0.189, 0.155]	-0.192	0.848
Humanitarian Aid Worker	-0.04	0.094	[-0.224, 0.145]	-0.422	0.673
Kidney Donor (ND)	0.249 ***	0.071	[0.110, 0.388]	3.519	< .0001
Heroic Rescuer	-0.235	0.132	[-0.494, 0.024]	-1.783	0.075
Liver Donor	-0.042	0.180	[-0.395, 0.311]	-0.234	0.815
Age	0.004	0.002	[-0.000, 0.009]	1.821	0.069
Sex (Female)	-0.132 *	0.056	[-0.242, -0.022]	-2.366	0.018

Note. F(8, 544) = 4.352; R^2 = 0.060; Groups are coded as indicator variables relative to the control group. Coefficients are unstandardized. SE indicates the standard error. 95% CI indicates lower/upper limits of the confidence interval. Intercept corresponds to the mean for controls who are not female at average age. * indicates p<.05, ** indicates p<.01, *** indicates p<.001. P-values are two-tailed. Multivariate regressions compared all altruistic groups against controls simultaneously (in lieu of separate tests for which corrections for multiple comparisons would be appropriate).

Table S3d-ii. Regression results for Agreeableness comparing altruistic groups to controls (N=532)

Agreeableness	Estimate	SE	95% CI	T	p
Intercept	3.455 ***	0.086	[3.286, 3.623]	40.261	< .0001
Marrow Donor	0.153	0.095	[-0.035, 0.340]	1.599	0.11
Kidney Donor (D)	-0.035	0.090	[-0.211, 0.141]	-0.394	0.694
Humanitarian Aid Worker	-0.037	0.096	[-0.225, 0.151]	-0.389	0.697
Kidney Donor (ND)	0.233 **	0.073	[0.090, 0.376]	3.193	0.001
Heroic Rescuer	-0.263	0.137	[-0.533, 0.007]	-1.914	0.056
Liver Donor	-0.183	0.190	[-0.556, 0.191]	-0.960	0.337
Age	0.005 *	0.003	[0.000, 0.010]	2.003	0.046
Sex (Female)	-0.136 *	0.057	[-0.249, -0.023]	-2.374	0.018
College Education	-0.092	0.069	[-0.228, 0.044]	-1.328	0.185
Income (≥$90k)	-0.037	0.056	[-0.147, 0.073]	-0.653	0.514

Note. F(10, 521) = 3.806; R^2 = 0.068; Groups are coded as indicator variables relative to the control group. Coefficients are unstandardized. SE indicates the standard error. 95% CI indicates lower/upper limits of the confidence interval. Intercept corresponds to the mean for controls who are not female at average age who never completed a four-year degree and household earns <$90,000. * indicates p<.05, ** indicates p<.01, *** indicates p<.001. P-values are two-tailed. Multivariate regressions compared all altruistic groups against controls simultaneously (in lieu of separate tests for which corrections for multiple comparisons would be appropriate).

Table S3e-i. Regression results for Conscientiousness comparing altruistic groups to controls (N=553)

Conscientiousness	Estimate	SE	95% CI	T	p
Intercept	3.768 ***	0.046	[3.677, 3.858]	81.607	< .0001
Marrow Donor	0.138	0.076	[-0.011, 0.286]	1.814	0.07
Kidney Donor (D)	0.066	0.072	[-0.075, 0.207]	0.920	0.358
Humanitarian Aid Worker	0.037	0.077	[-0.114, 0.188]	0.476	0.635
Kidney Donor (ND)	0.02	0.058	[-0.094, 0.134]	0.344	0.731
Heroic Rescuer	0.016	0.108	[-0.196, 0.229]	0.151	0.88
Liver Donor	0.156	0.147	[-0.134, 0.445]	1.055	0.292
Age	0.001	0.002	[-0.003, 0.005]	0.474	0.636
Sex (Female)	0.119 **	0.046	[0.029, 0.209]	2.596	0.01

Note. $F(8, 544) = 1.471$; $R^2 = 0.021$; Groups are coded as indicator variables relative to the control group. Coefficients are unstandardized. SE indicates the standard error. 95% CI indicates lower/upper limits of the confidence interval. Intercept corresponds to the mean for controls who are not female at average age. * indicates p<.05, ** indicates p<.01, *** indicates p<.001. P-values are two-tailed. Multivariate regressions compared all altruistic groups against controls simultaneously (in lieu of separate tests for which corrections for multiple comparisons would be appropriate).

Table S3e-ii. Regression results for Conscientiousness comparing altruistic groups to controls (N=532)

Conscientiousness	Estimate	SE	95% CI	T	p
Intercept	3.695 ***	0.070	[3.558, 3.832]	52.935	< .0001
Marrow Donor	0.121	0.078	[-0.032, 0.273]	1.553	0.121
Kidney Donor (D)	0.065	0.073	[-0.078, 0.209]	0.895	0.371
Humanitarian Aid Worker	0.041	0.078	[-0.112, 0.194]	0.528	0.598
Kidney Donor (ND)	0.014	0.059	[-0.103, 0.130]	0.232	0.816
Heroic Rescuer	0.014	0.112	[-0.205, 0.234]	0.128	0.899
Liver Donor	0.154	0.155	[-0.150, 0.458]	0.997	0.319
Age	-0.001	0.002	[-0.005, 0.003]	-0.560	0.576
Sex (Female)	0.114 *	0.047	[0.022, 0.206]	2.443	0.015
College Education	0.002	0.056	[-0.108, 0.113]	0.039	0.969
Income (≥$90k)	0.139 **	0.046	[0.050, 0.229]	3.058	0.002

Note. $F(10, 521) = 1.973$; $R^2 = 0.036$; Groups are coded as indicator variables relative to the control group. Coefficients are unstandardized. SE indicates the standard error. 95% CI indicates lower/upper limits of the confidence interval. Intercept corresponds to the mean for controls who are not female at average age who never completed a four-year degree and household earns <$90,000. * indicates p<.05, ** indicates p<.01, *** indicates p<.001. P-values are two-tailed. Multivariate regressions compared all altruistic groups against controls simultaneously (in lieu of separate tests for which corrections for multiple comparisons would be appropriate).

Table S3f-i. Regression results for Openness comparing altruistic groups to controls (N=553)

**Openness**	**Estimate**	**SE**	**95% CI**	**T**	**p**
Intercept	3.961 ***	0.054	[3.854, 4.068]	72.714	< .0001
Marrow Donor	-0.203 *	0.089	[-0.379, -0.027]	-2.267	0.024
Kidney Donor (D)	-0.294 ***	0.085	[-0.460, -0.127]	-3.470	0.001
Humanitarian Aid Worker	0.024	0.091	[-0.154, 0.202]	0.268	0.789
Kidney Donor (ND)	-0.095	0.068	[-0.230, 0.039]	-1.392	0.165
Heroic Rescuer	-0.489 ***	0.128	[-0.740, -0.239]	-3.834	< .0001
Liver Donor	-0.27	0.174	[-0.612, 0.071]	-1.554	0.121
Age	0.006 **	0.002	[0.002, 0.011]	2.797	0.005
Sex (Female)	-0.221 ***	0.054	[-0.327, -0.114]	-4.075	< .0001

Note. F(8, 544) = 5.540; R^2 = 0.075; Groups are coded as indicator variables relative to the control group. Coefficients are unstandardized. SE indicates the standard error. 95% CI indicates lower/upper limits of the confidence interval. Intercept corresponds to the mean for controls who are not female at average age. * indicates p<.05, ** indicates p<.01, *** indicates p<.001. P-values are two-tailed. Multivariate regressions compared all altruistic groups against controls simultaneously (in lieu of separate tests for which corrections for multiple comparisons would be appropriate).

Table S3f-ii. Regression results for Openness comparing altruistic groups to controls (N=532)

**Openness**	**Estimate**	**SE**	**95% CI**	**T**	**p**
Intercept	3.883 ***	0.082	[3.721, 4.045]	47.097	< .0001
Marrow Donor	-0.159	0.092	[-0.339, 0.021]	-1.733	0.084
Kidney Donor (D)	-0.275 **	0.086	[-0.444, -0.105]	-3.188	0.002
Humanitarian Aid Worker	0.022	0.092	[-0.159, 0.202]	0.234	0.815
Kidney Donor (ND)	-0.057	0.070	[-0.195, 0.080]	-0.818	0.414
Heroic Rescuer	-0.452 ***	0.132	[-0.712, -0.193]	-3.425	0.001
Liver Donor	-0.284	0.183	[-0.643, 0.075]	-1.552	0.121
Age	0.007 **	0.002	[0.002, 0.012]	2.937	0.003
Sex (Female)	-0.213 ***	0.055	[-0.322, -0.105]	-3.873	< .0001
College Education	0.109	0.067	[-0.021, 0.240]	1.642	0.101
Income (≥$90k)	-0.06	0.054	[-0.165, 0.046]	-1.105	0.27

Note. F(10, 521) = 4.658; R^2 = 0.082; Groups are coded as indicator variables relative to the control group. Coefficients are unstandardized. SE indicates the standard error. 95% CI indicates lower/upper limits of the confidence interval. Intercept corresponds to the mean for controls who are not female at average age who never completed a four-year degree and household earns <$90,000. * indicates p<.05, ** indicates p<.01, *** indicates p<.001. P-values are two-tailed. Multivariate regressions compared all altruistic groups against controls simultaneously (in lieu of separate tests for which corrections for multiple comparisons would be appropriate).

Table S4a-i. Regression results for Risk Taking (Ethical) comparing altruistic groups to controls (N=554)

Risk Taking (Ethical)	Estimate	SE	95% CI	T	p
Intercept	2.206 ***	0.078	[2.053, 2.359]	28.320	< .0001
Marrow Donor	-0.275 *	0.128	[-0.527, -0.024]	-2.150	0.032
Kidney Donor (D)	-0.137	0.121	[-0.375, 0.100]	-1.136	0.256
Humanitarian Aid Worker	-0.005	0.130	[-0.259, 0.250]	-0.036	0.971
Kidney Donor (ND)	-0.347 ***	0.098	[-0.539, -0.155]	-3.546	< .0001
Heroic Rescuer	-0.333	0.183	[-0.692, 0.025]	-1.827	0.068
Liver Donor	-0.403	0.249	[-0.892, 0.086]	-1.618	0.106
Age	-0.013 ***	0.003	[-0.020, -0.007]	-3.976	< .0001
Sex (Female)	-0.198 *	0.077	[-0.350, -0.046]	-2.553	0.011

Note. F(8, 545) = 6.375; R^2 = 0.086; Groups are coded as indicator variables relative to the control group. Coefficients are unstandardized. SE indicates the standard error. 95% CI indicates lower/upper limits of the confidence interval. Intercept corresponds to the mean for controls who are not female at average age. * indicates p<.05, ** indicates p<.01, *** indicates p<.001. P-values are two-tailed. Multivariate regressions compared all altruistic groups against controls simultaneously (in lieu of separate tests for which corrections for multiple comparisons would be appropriate).

Table S4a-ii. Regression results for Risk Taking (Ethical) comparing altruistic groups to controls (N=533)

Risk Taking (Ethical)	Estimate	SE	95% CI	T	p
Intercept	2.307 ***	0.118	[2.076, 2.539]	19.570	< .0001
Marrow Donor	-0.291 *	0.131	[-0.548, -0.033]	-2.215	0.027
Kidney Donor (D)	-0.147	0.123	[-0.389, 0.095]	-1.197	0.232
Humanitarian Aid Worker	0.016	0.131	[-0.242, 0.274]	0.123	0.902
Kidney Donor (ND)	-0.361 ***	0.100	[-0.557, -0.165]	-3.610	< .0001
Heroic Rescuer	-0.355	0.189	[-0.726, 0.016]	-1.880	0.061
Liver Donor	-0.409	0.261	[-0.922, 0.105]	-1.564	0.119
Age	-0.013 ***	0.003	[-0.020, -0.006]	-3.747	< .0001
Sex (Female)	-0.184 *	0.079	[-0.339, -0.029]	-2.336	0.02
College Education	-0.131	0.095	[-0.318, 0.056]	-1.379	0.168
Income (≥$90k)	0.002	0.077	[-0.149, 0.153]	0.027	0.978

Note. F(10, 522) = 4.769; R^2 = 0.084; Groups are coded as indicator variables relative to the control group. Coefficients are unstandardized. SE indicates the standard error. 95% CI indicates lower/upper limits of the confidence interval. Intercept corresponds to the mean for controls who are not female at average age who never completed a four-year degree and household earns <$90,000. * indicates p<.05, ** indicates p<.01, *** indicates p<.001. P-values are two-tailed. Multivariate regressions compared all altruistic groups against controls simultaneously (in lieu of separate tests for which corrections for multiple comparisons would be appropriate).

Table S4b-i. Regression results for Risk Taking (Financial) comparing altruistic groups to controls (N=554)

Risk Taking (Financial)	Estimate	SE	95% CI	T	p
Intercept	2.859 ***	0.091	[2.681, 3.037]	31.536	< .0001
Marrow Donor	0.099	0.149	[-0.194, 0.391]	0.663	0.508
Kidney Donor (D)	-0.075	0.141	[-0.352, 0.201]	-0.533	0.594
Humanitarian Aid Worker	0.216	0.151	[-0.081, 0.512]	1.431	0.153
Kidney Donor (ND)	0.04	0.114	[-0.183, 0.264]	0.355	0.723
Heroic Rescuer	0.271	0.212	[-0.146, 0.689]	1.278	0.202
Liver Donor	-0.143	0.290	[-0.712, 0.426]	-0.493	0.622
Age	-0.005	0.004	[-0.013, 0.003]	-1.309	0.191
Sex (Female)	-0.366 ***	0.090	[-0.543, -0.189]	-4.065	< .0001

Note. F(8, 545) = 3.612; R^2 = 0.050; Groups are coded as indicator variables relative to the control group. Coefficients are unstandardized. SE indicates the standard error. 95% CI indicates lower/upper limits of the confidence interval. Intercept corresponds to the mean for controls who are not female at average age. * indicates p<.05, ** indicates p<.01, *** indicates p<.001. P-values are two-tailed. Multivariate regressions compared all altruistic groups against controls simultaneously (in lieu of separate tests for which corrections for multiple comparisons would be appropriate).

Table S4b-ii. Regression results for Risk Taking (Financial) comparing altruistic groups to controls (N=533)

Risk Taking (Financial)	Estimate	SE	95% CI	T	p
Intercept	2.767 ***	0.139	[2.494, 3.039]	19.946	< .0001
Marrow Donor	0.141	0.154	[-0.162, 0.444]	0.915	0.361
Kidney Donor (D)	-0.064	0.145	[-0.348, 0.220]	-0.442	0.659
Humanitarian Aid Worker	0.225	0.155	[-0.078, 0.529]	1.457	0.146
Kidney Donor (ND)	0.044	0.118	[-0.187, 0.275]	0.378	0.706
Heroic Rescuer	0.317	0.222	[-0.119, 0.754]	1.427	0.154
Liver Donor	-0.225	0.307	[-0.829, 0.379]	-0.732	0.465
Age	-0.005	0.004	[-0.013, 0.003]	-1.269	0.205
Sex (Female)	-0.35 ***	0.093	[-0.532, -0.168]	-3.775	< .0001
College Education	0.051	0.112	[-0.169, 0.270]	0.453	0.651
Income (≥$90k)	0.075	0.090	[-0.103, 0.252]	0.825	0.41

Note. F(10, 522) = 2.885; R^2 = 0.052; Groups are coded as indicator variables relative to the control group. Coefficients are unstandardized. SE indicates the standard error. 95% CI indicates lower/upper limits of the confidence interval. Intercept corresponds to the mean for controls who are not female at average age who never completed a four-year degree and household earns <$90,000. * indicates p<.05, ** indicates p<.01, *** indicates p<.001. P-values are two-tailed. Multivariate regressions compared all altruistic groups against controls simultaneously (in lieu of separate tests for which corrections for multiple comparisons would be appropriate).

Table S4c-i. Regression results for Risk Taking (Health Safety) comparing altruistic groups to controls (N=554)

Risk Taking (Health Safety)	Estimate	SE	95% CI	T	p
Intercept	3.119 ***	0.102	[2.918, 3.320]	30.477	< .0001
Marrow Donor	-0.328	0.168	[-0.658, 0.003]	-1.948	0.052
Kidney Donor (D)	-0.152	0.159	[-0.464, 0.160]	-0.956	0.34
Humanitarian Aid Worker	0.172	0.170	[-0.163, 0.507]	1.008	0.314
Kidney Donor (ND)	0.042	0.128	[-0.210, 0.295]	0.330	0.742
Heroic Rescuer	0.642 **	0.240	[0.171, 1.113]	2.677	0.008
Liver Donor	-0.382	0.327	[-1.025, 0.261]	-1.168	0.243
Age	-0.033 ***	0.004	[-0.041, -0.024]	-7.555	< .0001
Sex (Female)	-0.516 ***	0.102	[-0.716, -0.317]	-5.076	< .0001

Note. F(8, 545) = 13.328; R^2 = 0.164; Groups are coded as indicator variables relative to the control group. Coefficients are unstandardized. SE indicates the standard error. 95% CI indicates lower/upper limits of the confidence interval. Intercept corresponds to the mean for controls who are not female at average age. * indicates p<.05, ** indicates p<.01, *** indicates p<.001. P-values are two-tailed. Multivariate regressions compared all altruistic groups against controls simultaneously (in lieu of separate tests for which corrections for multiple comparisons would be appropriate).

Table S4c-ii. Regression results for Risk Taking (Health Safety) comparing altruistic groups to controls (N=533)

Risk Taking (Health Safety)	Estimate	SE	95% CI	T	p
Intercept	3.532 ***	0.153	[3.231, 3.834]	23.027	< .0001
Marrow Donor	-0.443 **	0.171	[-0.778, -0.108]	-2.597	0.01
Kidney Donor (D)	-0.242	0.160	[-0.556, 0.073]	-1.509	0.132
Humanitarian Aid Worker	0.132	0.171	[-0.204, 0.468]	0.773	0.44
Kidney Donor (ND)	-0.048	0.130	[-0.304, 0.207]	-0.371	0.711
Heroic Rescuer	0.462	0.246	[-0.020, 0.945]	1.881	0.06
Liver Donor	-0.397	0.340	[-1.065, 0.271]	-1.169	0.243
Age	-0.033 ***	0.005	[-0.041, -0.024]	-7.187	< .0001
Sex (Female)	-0.517 ***	0.102	[-0.718, -0.316]	-5.045	< .0001
College Education	-0.37 **	0.124	[-0.613, -0.127]	-2.993	0.003
Income (≥$90k)	-0.07	0.100	[-0.266, 0.127]	-0.698	0.486

Note. F(10, 522) = 11.105; R^2 = 0.175; Groups are coded as indicator variables relative to the control group. Coefficients are unstandardized. SE indicates the standard error. 95% CI indicates lower/upper limits of the confidence interval. Intercept corresponds to the mean for controls who are not female at average age who never completed a four-year degree and household earns <$90,000. * indicates p<.05, ** indicates p<.01, *** indicates p<.001. P-values are two-tailed. Multivariate regressions compared all altruistic groups against controls simultaneously (in lieu of separate tests for which corrections for multiple comparisons would be appropriate).

Table S4d-i. Regression results for Risk Taking (Recreational) comparing altruistic groups to controls (N=554)

Risk Taking (Recreational)	Estimate	SE	95% CI	T	p
Intercept	3.995 ***	0.142	[3.716, 4.275]	28.111	< .0001
Marrow Donor	-0.083	0.234	[-0.542, 0.375]	-0.357	0.721
Kidney Donor (D)	-0.031	0.221	[-0.465, 0.403]	-0.140	0.889
Humanitarian Aid Worker	0.772 **	0.237	[0.307, 1.236]	3.260	0.001
Kidney Donor (ND)	0.494 **	0.178	[0.144, 0.845]	2.769	0.006
Heroic Rescuer	0.637	0.333	[-0.017, 1.291]	1.912	0.056
Liver Donor	0.482	0.454	[-0.410, 1.375]	1.061	0.289
Age	-0.032 ***	0.006	[-0.043, -0.020]	-5.247	< .0001
Sex (Female)	-1.143 ***	0.141	[-1.421, -0.866]	-8.089	< .0001

Note. F(8, 545) = 14.704; R^2 = 0.178; Groups are coded as indicator variables relative to the control group. Coefficients are unstandardized. SE indicates the standard error. 95% CI indicates lower/upper limits of the confidence interval. Intercept corresponds to the mean for controls who are not female at average age. * indicates p<.05, ** indicates p<.01, *** indicates p<.001. P-values are two-tailed. Multivariate regressions compared all altruistic groups against controls simultaneously (in lieu of separate tests for which corrections for multiple comparisons would be appropriate).

Table S4d-ii. Regression results for Risk Taking (Recreational) comparing altruistic groups to controls (N=533)

Risk Taking (Recreational)	Estimate	SE	95% CI	T	p
Intercept	4.062 ***	0.217	[3.636, 4.487]	18.755	< .0001
Marrow Donor	-0.061	0.241	[-0.534, 0.412]	-0.253	0.8
Kidney Donor (D)	-0.019	0.226	[-0.463, 0.425]	-0.084	0.933
Humanitarian Aid Worker	0.749 **	0.241	[0.275, 1.223]	3.105	0.002
Kidney Donor (ND)	0.489 **	0.184	[0.128, 0.849]	2.660	0.008
Heroic Rescuer	0.66	0.347	[-0.022, 1.341]	1.902	0.058
Liver Donor	0.262	0.480	[-0.681, 1.205]	0.546	0.585
Age	-0.03 ***	0.006	[-0.042, -0.017]	-4.674	< .0001
Sex (Female)	-1.15 ***	0.145	[-1.434, -0.866]	-7.947	< .0001
College Education	0.001	0.175	[-0.343, 0.344]	0.003	0.998
Income (≥$90k)	-0.098	0.141	[-0.376, 0.179]	-0.695	0.487

Note. F(10, 522) = 11.088; R^2 = 0.175; Groups are coded as indicator variables relative to the control group. Coefficients are unstandardized. SE indicates the standard error. 95% CI indicates lower/upper limits of the confidence interval. Intercept corresponds to the mean for controls who are not female at average age who never completed a four-year degree and household earns <$90,000. * indicates p<.05, ** indicates p<.01, *** indicates p<.001. P-values are two-tailed. Multivariate regressions compared all altruistic groups against controls simultaneously (in lieu of separate tests for which corrections for multiple comparisons would be appropriate).

Table S4e-i. Regression results for Risk Taking (Social) comparing altruistic groups to controls (N=554)

Risk Taking (Social)	Estimate	SE	95% CI	T	p
Intercept	5.421 ***	0.087	[5.250, 5.592]	62.418	< .0001
Marrow Donor	-0.372 **	0.143	[-0.652, -0.091]	-2.605	0.009
Kidney Donor (D)	-0.332 *	0.135	[-0.597, -0.067]	-2.464	0.014
Humanitarian Aid Worker	0.472 **	0.145	[0.188, 0.756]	3.263	0.001
Kidney Donor (ND)	-0.081	0.109	[-0.295, 0.133]	-0.741	0.459
Heroic Rescuer	-0.027	0.204	[-0.426, 0.373]	-0.130	0.896
Liver Donor	-0.059	0.278	[-0.605, 0.486]	-0.213	0.831
Age	-0.001	0.004	[-0.009, 0.006]	-0.391	0.696
Sex (Female)	0.014	0.086	[-0.156, 0.183]	0.159	0.874

Note. $F(8, 545) = 3.797$; $R^2 = 0.053$; Groups are coded as indicator variables relative to the control group. Coefficients are unstandardized. SE indicates the standard error. 95% CI indicates lower/upper limits of the confidence interval. Intercept corresponds to the mean for controls who are not female at average age. * indicates $p<.05$, ** indicates $p<.01$, *** indicates $p<.001$. P-values are two-tailed. Multivariate regressions compared all altruistic groups against controls simultaneously (in lieu of separate tests for which corrections for multiple comparisons would be appropriate).

Table S4e-ii. Regression results for Risk Taking (Social) comparing altruistic groups to controls (N=533)

Risk Taking (Social)	Estimate	SE	95% CI	T	p
Intercept	5.348 ***	0.132	[5.088, 5.607]	40.482	< .0001
Marrow Donor	-0.367 *	0.147	[-0.656, -0.078]	-2.498	0.013
Kidney Donor (D)	-0.325 *	0.138	[-0.596, -0.054]	-2.354	0.019
Humanitarian Aid Worker	0.451 **	0.147	[0.162, 0.740]	3.066	0.002
Kidney Donor (ND)	-0.066	0.112	[-0.286, 0.154]	-0.593	0.554
Heroic Rescuer	-0.01	0.212	[-0.426, 0.406]	-0.048	0.962
Liver Donor	0.051	0.293	[-0.524, 0.626]	0.175	0.861
Age	-0.001	0.004	[-0.009, 0.007]	-0.297	0.767
Sex (Female)	0.009	0.088	[-0.165, 0.182]	0.100	0.921
College Education	0.091	0.107	[-0.119, 0.300]	0.852	0.395
Income (≥$90k)	0.026	0.086	[-0.143, 0.195]	0.299	0.765

Note. $F(10, 522) = 2.952$; $R^2 = 0.054$; Groups are coded as indicator variables relative to the control group. Coefficients are unstandardized. SE indicates the standard error. 95% CI indicates lower/upper limits of the confidence interval. Intercept corresponds to the mean for controls who are not female at average age who never completed a four-year degree and household earns <$90,000. * indicates $p<.05$, ** indicates $p<.01$, *** indicates $p<.001$. P-values are two-tailed. Multivariate regressions compared all altruistic groups against controls simultaneously (in lieu of separate tests for which corrections for multiple comparisons would be appropriate).

Table S5a-i. Regression results for Risk Taking Perception (Ethical) comparing altruistic groups to controls (N=554)

Risk Taking Perception (Ethical)	Estimate	SE	95% CI	T	p
Intercept	4.669 ***	0.088	[4.497, 4.842]	53.069	< .0001
Marrow Donor	0.31 *	0.145	[0.026, 0.594]	2.142	0.033
Kidney Donor (D)	0.277 *	0.137	[0.008, 0.545]	2.025	0.043
Humanitarian Aid Worker	-0.141	0.146	[-0.429, 0.147]	-0.962	0.336
Kidney Donor (ND)	0.117	0.110	[-0.100, 0.334]	1.057	0.291
Heroic Rescuer	0.389	0.206	[-0.016, 0.794]	1.887	0.06
Liver Donor	0.111	0.281	[-0.442, 0.663]	0.394	0.694
Age	0.025 ***	0.004	[0.017, 0.032]	6.622	< .0001
Sex (Female)	0.415 ***	0.087	[0.243, 0.586]	4.740	< .0001

Note. F(8, 545) = 11.457; R^2 = 0.144; Groups are coded as indicator variables relative to the control group. Coefficients are unstandardized. SE indicates the standard error. 95% CI indicates lower/upper limits of the confidence interval. Intercept corresponds to the mean for controls who are not female at average age. * indicates p<.05, ** indicates p<.01, *** indicates p<.001. P-values are two-tailed. Multivariate regressions compared all altruistic groups against controls simultaneously (in lieu of separate tests for which corrections for multiple comparisons would be appropriate).

Table S5a-ii. Regression results for Risk Taking Perception (Ethical) comparing altruistic groups to controls (N=533)

Risk Taking Perception (Ethical)	Estimate	SE	95% CI	T	p
Intercept	4.826 ***	0.133	[4.564, 5.087]	36.260	< .0001
Marrow Donor	0.297 *	0.148	[0.006, 0.587]	2.003	0.046
Kidney Donor (D)	0.259	0.139	[-0.014, 0.532]	1.862	0.063
Humanitarian Aid Worker	-0.113	0.148	[-0.404, 0.179]	-0.760	0.448
Kidney Donor (ND)	0.107	0.113	[-0.115, 0.328]	0.945	0.345
Heroic Rescuer	0.326	0.213	[-0.092, 0.745]	1.531	0.126
Liver Donor	-0.105	0.295	[-0.684, 0.475]	-0.355	0.723
Age	0.023 ***	0.004	[0.015, 0.031]	5.806	< .0001
Sex (Female)	0.378 ***	0.089	[0.204, 0.553]	4.253	< .0001
College Education	-0.215 *	0.107	[-0.426, -0.004]	-2.003	0.046
Income (≥$90k)	0.059	0.087	[-0.112, 0.229]	0.676	0.499

Note. F(10, 522) = 8.960; R^2 = 0.147; Groups are coded as indicator variables relative to the control group. Coefficients are unstandardized. SE indicates the standard error. 95% CI indicates lower/upper limits of the confidence interval. Intercept corresponds to the mean for controls who are not female at average age who never completed a four-year degree and household earns <$90,000. * indicates p<.05, ** indicates p<.01, *** indicates p<.001. P-values are two-tailed. Multivariate regressions compared all altruistic groups against controls simultaneously (in lieu of separate tests for which corrections for multiple comparisons would be appropriate).

Table S5b-i. Regression results for Risk Taking Perception (Financial) comparing altruistic groups to controls (N=554)

Risk Taking Perception (Financial)	Estimate	SE	95% CI	T	p
Intercept	4.323 ***	0.081	[4.164, 4.483]	53.321	< .0001
Marrow Donor	-0.002	0.133	[-0.263, 0.260]	-0.013	0.99
Kidney Donor (D)	0.087	0.126	[-0.161, 0.334]	0.690	0.491
Humanitarian Aid Worker	-0.325 *	0.135	[-0.590, -0.060]	-2.407	0.016
Kidney Donor (ND)	-0.242 *	0.102	[-0.442, -0.043]	-2.382	0.018
Heroic Rescuer	-0.201	0.190	[-0.574, 0.173]	-1.056	0.291
Liver Donor	-0.033	0.259	[-0.542, 0.476]	-0.129	0.898
Age	0.013 ***	0.003	[0.007, 0.020]	3.880	< .0001
Sex (Female)	0.455 ***	0.081	[0.297, 0.614]	5.646	< .0001

Note. F(8, 545) = 7.841; R^2 = 0.103; Groups are coded as indicator variables relative to the control group. Coefficients are unstandardized. SE indicates the standard error. 95% CI indicates lower/upper limits of the confidence interval. Intercept corresponds to the mean for controls who are not female at average age. * indicates p<.05, ** indicates p<.01, *** indicates p<.001. P-values are two-tailed. Multivariate regressions compared all altruistic groups against controls simultaneously (in lieu of separate tests for which corrections for multiple comparisons would be appropriate).

Table S5b-ii. Regression results for Risk Taking Perception (Financial) comparing altruistic groups to controls (N=533)

Risk Taking Perception (Financial)	Estimate	SE	95% CI	T	p
Intercept	4.485 ***	0.122	[4.245, 4.725]	36.753	< .0001
Marrow Donor	0.017	0.136	[-0.250, 0.284]	0.125	0.9
Kidney Donor (D)	0.094	0.127	[-0.157, 0.344]	0.734	0.463
Humanitarian Aid Worker	-0.302 *	0.136	[-0.569, -0.035]	-2.222	0.027
Kidney Donor (ND)	-0.233 *	0.103	[-0.436, -0.029]	-2.248	0.025
Heroic Rescuer	-0.147	0.195	[-0.531, 0.237]	-0.752	0.452
Liver Donor	-0.112	0.271	[-0.643, 0.419]	-0.414	0.679
Age	0.014 ***	0.004	[0.007, 0.021]	3.789	< .0001
Sex (Female)	0.417 ***	0.082	[0.257, 0.578]	5.120	< .0001
College Education	-0.091	0.098	[-0.284, 0.103]	-0.920	0.358
Income (≥$90k)	-0.161 *	0.080	[-0.317, -0.005]	-2.021	0.044

Note. F(10, 522) = 5.968; R^2 = 0.103; Groups are coded as indicator variables relative to the control group. Coefficients are unstandardized. SE indicates the standard error. 95% CI indicates lower/upper limits of the confidence interval. Intercept corresponds to the mean for controls who are not female at average age who never completed a four-year degree and household earns <$90,000. * indicates p<.05, ** indicates p<.01, *** indicates p<.001. P-values are two-tailed. Multivariate regressions compared all altruistic groups against controls simultaneously (in lieu of separate tests for which corrections for multiple comparisons would be appropriate).

Table S5c-i. Regression results for Risk Taking Perception (Health Safety) comparing altruistic groups to controls (N=554)

**Risk Taking Perception (Health Safety)**	**Estimate**	**SE**	**95% CI**	**T**	**p**
Intercept	4.006 ***	0.082	[3.845, 4.167]	48.768	< .0001
Marrow Donor	0.214	0.135	[-0.051, 0.479]	1.587	0.113
Kidney Donor (D)	0.234	0.128	[-0.017, 0.484]	1.832	0.068
Humanitarian Aid Worker	-0.254	0.137	[-0.523, 0.015]	-1.857	0.064
Kidney Donor (ND)	-0.172	0.103	[-0.374, 0.031]	-1.665	0.097
Heroic Rescuer	0.156	0.193	[-0.222, 0.534]	0.809	0.419
Liver Donor	0.09	0.263	[-0.425, 0.606]	0.344	0.731
Age	0.018 ***	0.003	[0.011, 0.024]	5.043	< .0001
Sex (Female)	0.597 ***	0.082	[0.437, 0.757]	7.310	< .0001

Note. F(8, 545) = 12.277; R^2 = 0.153; Groups are coded as indicator variables relative to the control group. Coefficients are unstandardized. SE indicates the standard error. 95% CI indicates lower/upper limits of the confidence interval. Intercept corresponds to the mean for controls who are not female at average age. * indicates p<.05, ** indicates p<.01, *** indicates p<.001. P-values are two-tailed. Multivariate regressions compared all altruistic groups against controls simultaneously (in lieu of separate tests for which corrections for multiple comparisons would be appropriate).

Table S5c-ii. Regression results for Risk Taking Perception (Health Safety) comparing altruistic groups to controls (N=533)

**Risk Taking Perception (Health Safety)**	**Estimate**	**SE**	**95% CI**	**T**	**p**
Intercept	4.191 ***	0.125	[3.946, 4.436]	33.605	< .0001
Marrow Donor	0.178	0.139	[-0.094, 0.451]	1.285	0.199
Kidney Donor (D)	0.211	0.130	[-0.045, 0.467]	1.621	0.106
Humanitarian Aid Worker	-0.252	0.139	[-0.525, 0.021]	-1.816	0.07
Kidney Donor (ND)	-0.198	0.106	[-0.406, 0.010]	-1.872	0.062
Heroic Rescuer	0.129	0.200	[-0.263, 0.522]	0.648	0.517
Liver Donor	-0.056	0.276	[-0.599, 0.487]	-0.201	0.84
Age	0.016 ***	0.004	[0.009, 0.023]	4.406	< .0001
Sex (Female)	0.558 ***	0.083	[0.395, 0.722]	6.702	< .0001
College Education	-0.175	0.101	[-0.372, 0.023]	-1.739	0.083
Income (≥$90k)	-0.02	0.081	[-0.180, 0.139]	-0.250	0.802

Note. F(10, 522) = 9.079; R^2 = 0.148; Groups are coded as indicator variables relative to the control group. Coefficients are unstandardized. SE indicates the standard error. 95% CI indicates lower/upper limits of the confidence interval. Intercept corresponds to the mean for controls who are not female at average age who never completed a four-year degree and household earns <$90,000. * indicates p<.05, ** indicates p<.01, *** indicates p<.001. P-values are two-tailed. Multivariate regressions compared all altruistic groups against controls simultaneously (in lieu of separate tests for which corrections for multiple comparisons would be appropriate).

Table S5d-i. Regression results for Risk Taking Perception (Recreational) comparing altruistic groups to controls (N=554)

Risk Taking Perception (Recreational)	Estimate	SE	95% CI	T	p
Intercept	4.211 ***	0.076	[4.063, 4.360]	55.631	< .0001
Marrow Donor	0.126	0.124	[-0.118, 0.370]	1.013	0.312
Kidney Donor (D)	0.058	0.118	[-0.173, 0.289]	0.493	0.622
Humanitarian Aid Worker	-0.229	0.126	[-0.476, 0.019]	-1.815	0.07
Kidney Donor (ND)	-0.294 **	0.095	[-0.480, -0.107]	-3.090	0.002
Heroic Rescuer	-0.045	0.177	[-0.394, 0.303]	-0.254	0.799
Liver Donor	-0.202	0.242	[-0.678, 0.273]	-0.837	0.403
Age	0.013 ***	0.003	[0.007, 0.020]	4.140	< .0001
Sex (Female)	0.46 ***	0.075	[0.312, 0.608]	6.115	< .0001

Note. F(8, 545) = 8.565; R^2 = 0.112; Groups are coded as indicator variables relative to the control group. Coefficients are unstandardized. SE indicates the standard error. 95% CI indicates lower/upper limits of the confidence interval. Intercept corresponds to the mean for controls who are not female at average age. * indicates p<.05, ** indicates p<.01, *** indicates p<.001. P-values are two-tailed. Multivariate regressions compared all altruistic groups against controls simultaneously (in lieu of separate tests for which corrections for multiple comparisons would be appropriate).

Table S5d-ii. Regression results for Risk Taking Perception (Recreational) comparing altruistic groups to controls (N=533)

Risk Taking Perception (Recreational)	Estimate	SE	95% CI	T	p
Intercept	4.335 ***	0.116	[4.108, 4.563]	37.427	< .0001
Marrow Donor	0.101	0.129	[-0.152, 0.354]	0.784	0.433
Kidney Donor (D)	0.037	0.121	[-0.201, 0.275]	0.306	0.76
Humanitarian Aid Worker	-0.21	0.129	[-0.463, 0.044]	-1.627	0.104
Kidney Donor (ND)	-0.304 **	0.098	[-0.497, -0.111]	-3.098	0.002
Heroic Rescuer	-0.049	0.186	[-0.414, 0.315]	-0.265	0.791
Liver Donor	-0.196	0.257	[-0.701, 0.308]	-0.765	0.445
Age	0.013 ***	0.003	[0.006, 0.020]	3.779	< .0001
Sex (Female)	0.444 ***	0.077	[0.292, 0.596]	5.737	< .0001
College Education	-0.073	0.093	[-0.257, 0.110]	-0.785	0.433
Income (≥$90k)	-0.087	0.076	[-0.236, 0.061]	-1.153	0.249

Note. F(10, 522) = 6.274; R^2 = 0.107; Groups are coded as indicator variables relative to the control group. Coefficients are unstandardized. SE indicates the standard error. 95% CI indicates lower/upper limits of the confidence interval. Intercept corresponds to the mean for controls who are not female at average age who never completed a four-year degree and household earns <$90,000. * indicates p<.05, ** indicates p<.01, *** indicates p<.001. P-values are two-tailed. Multivariate regressions compared all altruistic groups against controls simultaneously (in lieu of separate tests for which corrections for multiple comparisons would be appropriate).

Table S5e-i. Regression results for Risk Taking Perception (Social) comparing altruistic groups to controls (N=554)

Risk Taking Perception (Social)	Estimate	SE	95% CI	T	p
Intercept	3.746 ***	0.067	[3.615, 3.876]	56.234	< .0001
Marrow Donor	0.052	0.109	[-0.163, 0.267]	0.471	0.638
Kidney Donor (D)	-0.043	0.103	[-0.246, 0.160]	-0.415	0.678
Humanitarian Aid Worker	-0.171	0.111	[-0.389, 0.047]	-1.544	0.123
Kidney Donor (ND)	-0.164	0.084	[-0.328, 0.001]	-1.957	0.051
Heroic Rescuer	0.07	0.156	[-0.237, 0.376]	0.447	0.655
Liver Donor	0.324	0.213	[-0.094, 0.742]	1.523	0.128
Age	0.002	0.003	[-0.004, 0.007]	0.610	0.542
Sex (Female)	0.257 ***	0.066	[0.127, 0.387]	3.880	< .0001

Note. F(8, 545) = 3.018; R^2 = 0.042; Groups are coded as indicator variables relative to the control group. Coefficients are unstandardized. SE indicates the standard error. 95% CI indicates lower/upper limits of the confidence interval. Intercept corresponds to the mean for controls who are not female at average age. * indicates p<.05, ** indicates p<.01, *** indicates p<.001. P-values are two-tailed. Multivariate regressions compared all altruistic groups against controls simultaneously (in lieu of separate tests for which corrections for multiple comparisons would be appropriate).

Table S5e-ii. Regression results for Risk Taking Perception (Social) comparing altruistic groups to controls (N=533)

Risk Taking Perception (Social)	Estimate	SE	95% CI	T	p
Intercept	3.856 ***	0.100	[3.659, 4.053]	38.468	< .0001
Marrow Donor	0.023	0.112	[-0.196, 0.242]	0.203	0.839
Kidney Donor (D)	-0.048	0.105	[-0.253, 0.158]	-0.455	0.649
Humanitarian Aid Worker	-0.137	0.112	[-0.356, 0.083]	-1.225	0.221
Kidney Donor (ND)	-0.17 *	0.085	[-0.337, -0.003]	-1.996	0.046
Heroic Rescuer	0.059	0.161	[-0.256, 0.375]	0.370	0.712
Liver Donor	0.242	0.222	[-0.194, 0.679]	1.091	0.276
Age	0	0.003	[-0.006, 0.006]	-0.021	0.984
Sex (Female)	0.215 **	0.067	[0.084, 0.347]	3.213	0.001
College Education	-0.125	0.081	[-0.283, 0.034]	-1.543	0.123
Income (≥$90k)	0.027	0.065	[-0.102, 0.155]	0.408	0.683

Note. F(10, 522) = 2.087; R^2 = 0.038; Groups are coded as indicator variables relative to the control group. Coefficients are unstandardized. SE indicates the standard error. 95% CI indicates lower/upper limits of the confidence interval. Intercept corresponds to the mean for controls who are not female at average age who never completed a four-year degree and household earns <$90,000. * indicates p<.05, ** indicates p<.01, *** indicates p<.001. P-values are two-tailed. Multivariate regressions compared all altruistic groups against controls simultaneously (in lieu of separate tests for which corrections for multiple comparisons would be appropriate).

Table S6a-i. Regression results for Perspective Taking comparing altruistic groups to controls (N=554)

Perspective Taking	Estimate	SE	95% CI	T	p
Intercept	26.365 ***	0.430	[25.520, 27.210]	61.273	< .0001
Marrow Donor	-0.104	0.707	[-1.493, 1.285]	-0.147	0.883
Kidney Donor (D)	-0.258	0.668	[-1.570, 1.055]	-0.385	0.7
Humanitarian Aid Worker	0.819	0.716	[-0.588, 2.227]	1.144	0.253
Kidney Donor (ND)	1.178 *	0.540	[0.117, 2.239]	2.181	0.03
Heroic Rescuer	-1.772	1.008	[-3.753, 0.209]	-1.757	0.079
Liver Donor	-0.133	1.375	[-2.835, 2.568]	-0.097	0.923
Age	0.012	0.018	[-0.024, 0.048]	0.665	0.506
Sex (Female)	0.205	0.428	[-0.636, 1.045]	0.478	0.633

Note. F(8, 545) = 1.847; R^2 = 0.026; Groups are coded as indicator variables relative to the control group. Coefficients are unstandardized. SE indicates the standard error. 95% CI indicates lower/upper limits of the confidence interval. Intercept corresponds to the mean for controls who are not female at average age. * indicates p<.05, ** indicates p<.01, *** indicates p<.001. P-values are two-tailed. Multivariate regressions compared all altruistic groups against controls simultaneously (in lieu of separate tests for which corrections for multiple comparisons would be appropriate).

Table S6a-ii. Regression results for Perspective Taking comparing altruistic groups to controls (N=533)

Perspective Taking	Estimate	SE	95% CI	T	p
Intercept	27.027 ***	0.648	[25.754, 28.300]	41.717	< .0001
Marrow Donor	-0.09	0.721	[-1.507, 1.326]	-0.125	0.9
Kidney Donor (D)	-0.377	0.676	[-1.705, 0.952]	-0.557	0.578
Humanitarian Aid Worker	0.867	0.722	[-0.551, 2.285]	1.201	0.23
Kidney Donor (ND)	1.01	0.549	[-0.070, 2.089]	1.838	0.067
Heroic Rescuer	-2.019	1.038	[-4.058, 0.020]	-1.946	0.052
Liver Donor	-1.233	1.436	[-4.054, 1.588]	-0.858	0.391
Age	0.009	0.019	[-0.029, 0.046]	0.454	0.65
Sex (Female)	0.133	0.433	[-0.718, 0.983]	0.307	0.759
College Education	-0.721	0.522	[-1.748, 0.305]	-1.381	0.168
Income (≥$90k)	0.13	0.423	[-0.700, 0.960]	0.308	0.758

Note. F(10, 522) = 1.595; R^2 = 0.030; Groups are coded as indicator variables relative to the control group. Coefficients are unstandardized. SE indicates the standard error. 95% CI indicates lower/upper limits of the confidence interval. Intercept corresponds to the mean for controls who are not female at average age who never completed a four-year degree and household earns <$90,000. * indicates p<.05, ** indicates p<.01, *** indicates p<.001. P-values are two-tailed. Multivariate regressions compared all altruistic groups against controls simultaneously (in lieu of separate tests for which corrections for multiple comparisons would be appropriate).

Table S6b-i. Regression results for Fantasy comparing altruistic groups to controls (N=554)

**Fantasy**	**Estimate**	**SE**	**95% CI**	**T**	**p**
Intercept	22.584 ***	0.509	[21.583, 23.584]	44.346	< .0001
Marrow Donor	-0.729	0.837	[-2.373, 0.915]	-0.871	0.384
Kidney Donor (D)	-0.528	0.791	[-2.081, 1.026]	-0.667	0.505
Humanitarian Aid Worker	-0.174	0.848	[-1.839, 1.492]	-0.205	0.838
Kidney Donor (ND)	0.867	0.639	[-0.389, 2.123]	1.356	0.176
Heroic Rescuer	-0.576	1.193	[-2.921, 1.768]	-0.483	0.629
Liver Donor	1.478	1.628	[-1.719, 4.676]	0.908	0.364
Age	-0.048 *	0.022	[-0.090, -0.006]	-2.223	0.027
Sex (Female)	1.827 ***	0.506	[0.832, 2.821]	3.608	< .0001

Note. F(8, 545) = 3.360; R^2 = 0.047; Groups are coded as indicator variables relative to the control group. Coefficients are unstandardized. SE indicates the standard error. 95% CI indicates lower/upper limits of the confidence interval. Intercept corresponds to the mean for controls who are not female at average age. * indicates p<.05, ** indicates p<.01, *** indicates p<.001. P-values are two-tailed. Multivariate regressions compared all altruistic groups against controls simultaneously (in lieu of separate tests for which corrections for multiple comparisons would be appropriate).

Table S6b-ii. Regression results for Fantasy comparing altruistic groups to controls (N=533)

**Fantasy**	**Estimate**	**SE**	**95% CI**	**T**	**p**
Intercept	20.962 ***	0.773	[19.443, 22.480]	27.112	< .0001
Marrow Donor	-0.558	0.860	[-2.248, 1.132]	-0.649	0.517
Kidney Donor (D)	-0.487	0.807	[-2.072, 1.099]	-0.603	0.547
Humanitarian Aid Worker	-0.189	0.861	[-1.881, 1.503]	-0.220	0.826
Kidney Donor (ND)	0.954	0.656	[-0.334, 2.242]	1.455	0.146
Heroic Rescuer	0.143	1.238	[-2.290, 2.576]	0.116	0.908
Liver Donor	1.571	1.714	[-1.796, 4.938]	0.917	0.36
Age	-0.041	0.023	[-0.086, 0.004]	-1.789	0.074
Sex (Female)	2.059 ***	0.517	[1.044, 3.074]	3.986	< .0001
College Education	1.716 **	0.623	[0.491, 2.941]	2.753	0.006
Income (≥$90k)	0.146	0.504	[-0.844, 1.137]	0.290	0.772

Note. F(10, 522) = 3.493; R^2 = 0.063; Groups are coded as indicator variables relative to the control group. Coefficients are unstandardized. SE indicates the standard error. 95% CI indicates lower/upper limits of the confidence interval. Intercept corresponds to the mean for controls who are not female at average age who never completed a four-year degree and household earns <$90,000. * indicates p<.05, ** indicates p<.01, *** indicates p<.001. P-values are two-tailed. Multivariate regressions compared all altruistic groups against controls simultaneously (in lieu of separate tests for which corrections for multiple comparisons would be appropriate).

Table S6c-i. Regression results for Empathic Concern comparing altruistic groups to controls (N=554)

Empathic Concern	Estimate	SE	95% CI	T	p
Intercept	26.084 ***	0.427	[25.246, 26.923]	61.110	< .0001
Marrow Donor	-0.039	0.701	[-1.417, 1.339]	-0.056	0.955
Kidney Donor (D)	0.704	0.663	[-0.599, 2.006]	1.061	0.289
Humanitarian Aid Worker	1.606 *	0.711	[0.210, 3.002]	2.259	0.024
Kidney Donor (ND)	2.343 ***	0.536	[1.291, 3.396]	4.372	< .0001
Heroic Rescuer	0.061	1.000	[-1.904, 2.026]	0.061	0.951
Liver Donor	1.383	1.364	[-1.297, 4.063]	1.013	0.311
Age	0.05 **	0.018	[0.014, 0.085]	2.752	0.006
Sex (Female)	2.637 ***	0.424	[1.804, 3.471]	6.215	< .0001

Note. F(8, 545) = 11.229; R^2 = 0.142; Groups are coded as indicator variables relative to the control group. Coefficients are unstandardized. SE indicates the standard error. 95% CI indicates lower/upper limits of the confidence interval. Intercept corresponds to the mean for controls who are not female at average age. * indicates p<.05, ** indicates p<.01, *** indicates p<.001. P-values are two-tailed. Multivariate regressions compared all altruistic groups against controls simultaneously (in lieu of separate tests for which corrections for multiple comparisons would be appropriate).

Table S6c-ii. Regression results for Empathic Concern comparing altruistic groups to controls (N=533)

Empathic Concern	Estimate	SE	95% CI	T	p
Intercept	26.603 ***	0.649	[25.327, 27.878]	40.969	< .0001
Marrow Donor	-0.12	0.722	[-1.539, 1.299]	-0.166	0.868
Kidney Donor (D)	0.639	0.678	[-0.692, 1.971]	0.943	0.346
Humanitarian Aid Worker	1.729 *	0.723	[0.307, 3.150]	2.389	0.017
Kidney Donor (ND)	2.257 ***	0.551	[1.176, 3.339]	4.100	< .0001
Heroic Rescuer	-0.118	1.040	[-2.162, 1.925]	-0.114	0.909
Liver Donor	0.799	1.439	[-2.029, 3.627]	0.555	0.579
Age	0.044 *	0.019	[0.006, 0.081]	2.280	0.023
Sex (Female)	2.618 ***	0.434	[1.766, 3.470]	6.035	< .0001
College Education	-0.632	0.524	[-1.661, 0.396]	-1.208	0.228
Income (≥$90k)	0.108	0.423	[-0.724, 0.940]	0.254	0.799

Note. F(10, 522) = 8.698; R^2 = 0.143; Groups are coded as indicator variables relative to the control group. Coefficients are unstandardized. SE indicates the standard error. 95% CI indicates lower/upper limits of the confidence interval. Intercept corresponds to the mean for controls who are not female at average age who never completed a four-year degree and household earns <$90,000. * indicates p<.05, ** indicates p<.01, *** indicates p<.001. P-values are two-tailed. Multivariate regressions compared all altruistic groups against controls simultaneously (in lieu of separate tests for which corrections for multiple comparisons would be appropriate).

Table S6d-i. Regression results for Personal Distress comparing altruistic groups to controls (N=554)

Personal Distress	Estimate	SE	95% CI	T	p
Intercept	15.454 ***	0.466	[14.540, 16.369]	33.193	< .0001
Marrow Donor	-0.95	0.765	[-2.453, 0.553]	-1.241	0.215
Kidney Donor (D)	-1.582 *	0.723	[-3.002, -0.161]	-2.187	0.029
Humanitarian Aid Worker	-3.186 ***	0.775	[-4.709, -1.663]	-4.110	< .0001
Kidney Donor (ND)	-1.356 *	0.585	[-2.504, -0.208]	-2.320	0.021
Heroic Rescuer	-3.552 **	1.091	[-5.695, -1.408]	-3.255	0.001
Liver Donor	-2.043	1.488	[-4.967, 0.880]	-1.373	0.17
Age	-0.031	0.020	[-0.070, 0.007]	-1.598	0.111
Sex (Female)	1.874 ***	0.463	[0.965, 2.783]	4.048	< .0001

Note. F(8, 545) = 7.181; R^2 = 0.095; Groups are coded as indicator variables relative to the control group. Coefficients are unstandardized. SE indicates the standard error. 95% CI indicates lower/upper limits of the confidence interval. Intercept corresponds to the mean for controls who are not female at average age. * indicates p<.05, ** indicates p<.01, *** indicates p<.001. P-values are two-tailed. Multivariate regressions compared all altruistic groups against controls simultaneously (in lieu of separate tests for which corrections for multiple comparisons would be appropriate).

Table S6d-ii. Regression results for Personal Distress comparing altruistic groups to controls (N=533)

Personal Distress	Estimate	SE	95% CI	T	p
Intercept	15.434 ***	0.707	[14.044, 16.823]	21.822	< .0001
Marrow Donor	-0.645	0.787	[-2.191, 0.901]	-0.819	0.413
Kidney Donor (D)	-1.367	0.738	[-2.817, 0.084]	-1.851	0.065
Humanitarian Aid Worker	-3.095 ***	0.788	[-4.643, -1.547]	-3.928	< .0001
Kidney Donor (ND)	-1.15	0.600	[-2.328, 0.028]	-1.917	0.056
Heroic Rescuer	-3.254 **	1.133	[-5.479, -1.028]	-2.872	0.004
Liver Donor	-2.057	1.568	[-5.137, 1.023]	-1.312	0.19
Age	-0.023	0.021	[-0.064, 0.018]	-1.109	0.268
Sex (Female)	1.901 ***	0.473	[0.973, 2.830]	4.024	< .0001
College Education	0.215	0.570	[-0.905, 1.336]	0.378	0.706
Income (≥$90k)	-0.678	0.461	[-1.584, 0.229]	-1.469	0.142

Note. F(10, 522) = 5.519; R^2 = 0.096; Groups are coded as indicator variables relative to the control group. Coefficients are unstandardized. SE indicates the standard error. 95% CI indicates lower/upper limits of the confidence interval. Intercept corresponds to the mean for controls who are not female at average age who never completed a four-year degree and household earns <$90,000. * indicates p<.05, ** indicates p<.01, *** indicates p<.001. P-values are two-tailed. Multivariate regressions compared all altruistic groups against controls simultaneously (in lieu of separate tests for which corrections for multiple comparisons would be appropriate).

Table S7a-i. Regression results for Fearless Dominance comparing altruistic groups to controls (N=554)

Fearless Dominance	Estimate	SE	95% CI	T	p
Intercept	55.301 ***	0.875	[53.583, 57.020]	63.213	< .0001
Marrow Donor	-0.894	1.438	[-3.718, 1.930]	-0.622	0.534
Kidney Donor (D)	-0.448	1.359	[-3.118, 2.221]	-0.330	0.742
Humanitarian Aid Worker	3.648 *	1.457	[0.786, 6.509]	2.504	0.013
Kidney Donor (ND)	3.557 **	1.098	[1.399, 5.715]	3.238	0.001
Heroic Rescuer	4.562 *	2.050	[0.535, 8.589]	2.225	0.026
Liver Donor	3.396	2.796	[-2.097, 8.889]	1.214	0.225
Age	-0.008	0.037	[-0.081, 0.065]	-0.217	0.828
Sex (Female)	-6.455 ***	0.870	[-8.163, -4.746]	-7.421	< .0001

Note. F(8, 545) = 11.359; R^2 = 0.143; Groups are coded as indicator variables relative to the control group. Coefficients are unstandardized. SE indicates the standard error. 95% CI indicates lower/upper limits of the confidence interval. Intercept corresponds to the mean for controls who are not female at average age. * indicates p<.05, ** indicates p<.01, *** indicates p<.001. P-values are two-tailed. Multivariate regressions compared all altruistic groups against controls simultaneously (in lieu of separate tests for which corrections for multiple comparisons would be appropriate).

Table S7a-ii. Regression results for Fearless Dominance comparing altruistic groups to controls (N=533)

Fearless Dominance	Estimate	SE	95% CI	T	p
Intercept	56.572 ***	1.334	[53.951, 59.194]	42.396	< .0001
Marrow Donor	-1.116	1.485	[-4.033, 1.801]	-0.752	0.453
Kidney Donor (D)	-0.452	1.393	[-3.189, 2.285]	-0.324	0.746
Humanitarian Aid Worker	3.619 *	1.487	[0.698, 6.540]	2.434	0.015
Kidney Donor (ND)	3.507 **	1.131	[1.284, 5.730]	3.100	0.002
Heroic Rescuer	4.398 *	2.137	[0.199, 8.597]	2.058	0.04
Liver Donor	1.299	2.958	[-4.512, 7.110]	0.439	0.661
Age	0.002	0.039	[-0.076, 0.079]	0.042	0.966
Sex (Female)	-6.589 ***	0.892	[-8.341, -4.838]	-7.391	< .0001
College Education	-0.935	1.076	[-3.049, 1.179]	-0.869	0.385
Income (≥$90k)	-0.638	0.870	[-2.347, 1.072]	-0.733	0.464

Note. F(10, 522) = 9.016; R^2 = 0.147; Groups are coded as indicator variables relative to the control group. Coefficients are unstandardized. SE indicates the standard error. 95% CI indicates lower/upper limits of the confidence interval. Intercept corresponds to the mean for controls who are not female at average age who never completed a four-year degree and household earns <$90,000. * indicates p<.05, ** indicates p<.01, *** indicates p<.001. P-values are two-tailed. Multivariate regressions compared all altruistic groups against controls simultaneously (in lieu of separate tests for which corrections for multiple comparisons would be appropriate).

Table S7b-i. Regression results for Coldheartedness comparing altruistic groups to controls (N=554)

Coldheartedness	Estimate	SE	95% CI	T	p
Intercept	15.527 ***	0.321	[14.896, 16.158]	48.324	< .0001
Marrow Donor	-0.125	0.528	[-1.162, 0.913]	-0.236	0.814
Kidney Donor (D)	-0.479	0.499	[-1.460, 0.501]	-0.960	0.337
Humanitarian Aid Worker	-1.299 *	0.535	[-2.350, -0.248]	-2.428	0.016
Kidney Donor (ND)	-1.729 ***	0.403	[-2.521, -0.936]	-4.285	< .0001
Heroic Rescuer	-0.376	0.753	[-1.855, 1.104]	-0.499	0.618
Liver Donor	-1.152	1.027	[-3.170, 0.865]	-1.122	0.262
Age	-0.013	0.014	[-0.040, 0.014]	-0.951	0.342
Sex (Female)	-2.118 ***	0.319	[-2.745, -1.490]	-6.630	< .0001

Note. F(8, 545) = 9.944; R^2 = 0.127; Groups are coded as indicator variables relative to the control group. Coefficients are unstandardized. SE indicates the standard error. 95% CI indicates lower/upper limits of the confidence interval. Intercept corresponds to the mean for controls who are not female at average age. * indicates p<.05, ** indicates p<.01, *** indicates p<.001. P-values are two-tailed. Multivariate regressions compared all altruistic groups against controls simultaneously (in lieu of separate tests for which corrections for multiple comparisons would be appropriate).

Table S7b-ii. Regression results for Coldheartedness comparing altruistic groups to controls (N=533)

Coldheartedness	Estimate	SE	95% CI	T	p
Intercept	14.768 ***	0.489	[13.807, 15.728]	30.214	< .0001
Marrow Donor	-0.073	0.544	[-1.141, 0.995]	-0.134	0.893
Kidney Donor (D)	-0.391	0.510	[-1.393, 0.612]	-0.766	0.444
Humanitarian Aid Worker	-1.329 *	0.545	[-2.399, -0.259]	-2.441	0.015
Kidney Donor (ND)	-1.641 ***	0.414	[-2.455, -0.827]	-3.960	< .0001
Heroic Rescuer	-0.119	0.783	[-1.657, 1.420]	-0.151	0.88
Liver Donor	-0.418	1.083	[-2.547, 1.710]	-0.386	0.699
Age	-0.011	0.014	[-0.039, 0.017]	-0.761	0.447
Sex (Female)	-2.009 ***	0.327	[-2.651, -1.368]	-6.152	< .0001
College Education	0.734	0.394	[-0.040, 1.508]	1.862	0.063
Income (≥$90k)	0.112	0.319	[-0.514, 0.738]	0.351	0.726

Note. F(10, 522) = 7.731; R^2 = 0.129; Groups are coded as indicator variables relative to the control group. Coefficients are unstandardized. SE indicates the standard error. 95% CI indicates lower/upper limits of the confidence interval. Intercept corresponds to the mean for controls who are not female at average age who never completed a four-year degree and household earns <$90,000. * indicates p<.05, ** indicates p<.01, *** indicates p<.001. P-values are two-tailed. Multivariate regressions compared all altruistic groups against controls simultaneously (in lieu of separate tests for which corrections for multiple comparisons would be appropriate).

Table S7c-i. Regression results for Self-centered Impulsivity comparing altruistic groups to controls (N=554)

Self-centered Impulsivity	Estimate	SE	95% CI	T	p
Intercept	49.83 ***	0.905	[48.052, 51.608]	55.057	< .0001
Marrow Donor	-3.625 *	1.487	[-6.547, -0.703]	-2.437	0.015
Kidney Donor (D)	-0.96	1.406	[-3.721, 1.802]	-0.683	0.495
Humanitarian Aid Worker	3.096 *	1.507	[0.136, 6.057]	2.055	0.04
Kidney Donor (ND)	-1.869	1.136	[-4.101, 0.363]	-1.645	0.101
Heroic Rescuer	0.173	2.121	[-3.993, 4.340]	0.082	0.935
Liver Donor	1.803	2.893	[-3.880, 7.485]	0.623	0.533
Age	-0.104 **	0.038	[-0.179, -0.029]	-2.716	0.007
Sex (Female)	-2.144 *	0.900	[-3.912, -0.377]	-2.383	0.018

Note. F(8, 545) = 3.963; R^2 = 0.055; Groups are coded as indicator variables relative to the control group. Coefficients are unstandardized. SE indicates the standard error. 95% CI indicates lower/upper limits of the confidence interval. Intercept corresponds to the mean for controls who are not female at average age. * indicates p<.05, ** indicates p<.01, *** indicates p<.001. P-values are two-tailed. Multivariate regressions compared all altruistic groups against controls simultaneously (in lieu of separate tests for which corrections for multiple comparisons would be appropriate).

Table S7c-ii. Regression results for Self-centered Impulsivity comparing altruistic groups to controls (N=533)

Self-centered Impulsivity	Estimate	SE	95% CI	T	p
Intercept	54.126 ***	1.346	[51.482, 56.769]	40.221	< .0001
Marrow Donor	-3.397 *	1.497	[-6.339, -0.456]	-2.269	0.024
Kidney Donor (D)	-1.099	1.405	[-3.859, 1.660]	-0.783	0.434
Humanitarian Aid Worker	3.328 *	1.499	[0.383, 6.273]	2.220	0.027
Kidney Donor (ND)	-1.824	1.141	[-4.065, 0.418]	-1.598	0.111
Heroic Rescuer	-0.21	2.156	[-4.445, 4.024]	-0.098	0.922
Liver Donor	0.461	2.983	[-5.400, 6.321]	0.154	0.877
Age	-0.074	0.040	[-0.152, 0.004]	-1.855	0.064
Sex (Female)	-2.356 **	0.899	[-4.123, -0.590]	-2.621	0.009
College Education	-2.857 **	1.085	[-4.989, -0.726]	-2.633	0.009
Income (≥$90k)	-3.636 ***	0.878	[-5.360, -1.912]	-4.143	< .0001

Note. F(10, 522) = 5.967; R^2 = 0.103; Groups are coded as indicator variables relative to the control group. Coefficients are unstandardized. SE indicates the standard error. 95% CI indicates lower/upper limits of the confidence interval. Intercept corresponds to the mean for controls who are not female at average age who never completed a four-year degree and household earns <$90,000. * indicates p<.05, ** indicates p<.01, *** indicates p<.001. P-values are two-tailed. Multivariate regressions compared all altruistic groups against controls simultaneously (in lieu of separate tests for which corrections for multiple comparisons would be appropriate).

Table S8-i. Regression results for CRT comparing altruistic groups to controls (N=506)

CRT	Estimate	SE	95% CI	T	p
Intercept	0.474 ***	0.046	[0.383, 0.564]	10.266	< .0001
Marrow Donor	0.093	0.074	[-0.052, 0.239]	1.264	0.207
Kidney Donor (D)	0.01	0.070	[-0.127, 0.148]	0.149	0.882
Humanitarian Aid Worker	-0.005	0.075	[-0.152, 0.142]	-0.061	0.951
Kidney Donor (ND)	0.056	0.063	[-0.069, 0.181]	0.882	0.378
Heroic Rescuer	0.023	0.111	[-0.196, 0.241]	0.206	0.837
Liver Donor	0.042	0.144	[-0.240, 0.324]	0.293	0.769
Age	-0.004	0.002	[-0.007, 0.000]	-1.762	0.079
Sex (Female)	-0.164 ***	0.047	[-0.257, -0.071]	-3.475	0.001

Note. F(8, 497) = 2.522; R^2 = 0.039; Groups are coded as indicator variables relative to the control group. Coefficients are unstandardized. SE indicates the standard error. 95% CI indicates lower/upper limits of the confidence interval. Intercept corresponds to the mean for controls who are not female at average age. * indicates p<.05, ** indicates p<.01, *** indicates p<.001. P-values are two-tailed. Multivariate regressions compared all altruistic groups against controls simultaneously (in lieu of separate tests for which corrections for multiple comparisons would be appropriate).

Table S8-ii. Regression results for CRT comparing altruistic groups to controls (N=485)

CRT	Estimate	SE	95% CI	T	p
Intercept	0.304 ***	0.070	[0.167, 0.442]	4.361	< .0001
Marrow Donor	0.097	0.075	[-0.050, 0.244]	1.293	0.197
Kidney Donor (D)	0.004	0.071	[-0.135, 0.142]	0.050	0.96
Humanitarian Aid Worker	-0.029	0.075	[-0.177, 0.118]	-0.388	0.698
Kidney Donor (ND)	0.075	0.064	[-0.052, 0.201]	1.164	0.245
Heroic Rescuer	0.084	0.114	[-0.140, 0.308]	0.734	0.463
Liver Donor	0.115	0.149	[-0.179, 0.408]	0.768	0.443
Age	-0.004 *	0.002	[-0.008, -0.000]	-2.011	0.045
Sex (Female)	-0.163 ***	0.048	[-0.256, -0.070]	-3.431	0.001
College Education	0.157 **	0.057	[0.046, 0.269]	2.765	0.006
Income (≥$90k)	0.088	0.046	[-0.002, 0.178]	1.925	0.055

Note. F(10, 474) = 3.770; R^2 = 0.074; Groups are coded as indicator variables relative to the control group. Coefficients are unstandardized. SE indicates the standard error. 95% CI indicates lower/upper limits of the confidence interval. Intercept corresponds to the mean for controls who are not female at average age who never completed a four-year degree and household earns <$90,000. * indicates p<.05, ** indicates p<.01, *** indicates p<.001. P-values are two-tailed. Multivariate regressions compared all altruistic groups against controls simultaneously (in lieu of separate tests for which corrections for multiple comparisons would be appropriate).

Table S9. Table of demographics of social discounting sub-sample

		Control	Combined Altruists	Bone Marrow Donor	Kidney Donor (D)	Humanitarian Aid Worker	Kidney Donor (ND)	Heroic Rescuer	Liver Donor
N = 275		58	217	36	57	41	58	14	11
Sex	F : M	39 : 19	137 : 80	17 : 19	46 : 11	29 : 12	37 : 21	0 : 14	8 : 3
Age	M (SE)	39.09 (1.25)	44.13 (.88)	35.11 (1.87)	45.86 (1.65)	41.20 (1.70)	5.19 (1.69)	48.57 (3.61)	38.09 (2.43)
Household Income									
Under $9,999		1	5	3	1	-	1	-	-
$10,000-14,999		1	1	-	-	1	-	-	-
$15,000-24,999		-	6	2	-	3	-	-	1
$25,000-39,999		2	15	1	4	1	8	1	-
$40,000-59,999		6	27	5	6	7	7	1	1
$60,000-89,999		17	35	4	12	8	6	3	2
$90,000-179,999		21	84	17	26	12	20	3	6
Over $180,000		5	38	3	7	8	15	5	-
Don't know / No response		5	6	1	1	1	1	1	1
Race									
White		40	185	22	55	32	53	13	10
African American / Black		7	5	4	-	-	1	-	-
Asian / Pacific Islander		5	10	3	1	3	3	-	-
Latino		1	4	2	-	1	-	-	1
Native American/Alaskan		-	1	1	-	-	-	-	-
Multiracial		4	6	3	-	1	1	1	-
Other		1	5	1	1	3	-	-	-
Prefer not to Respond		-	1	-	-	1	-	-	-
Hispanic									
Yes		1	4	2	-	1	-	-	1
No		57	213	34	57	40	58	14	10
Education									
High School		2	10	2	2	-	3	2	1
Some College		6	40	6	9	4	12	4	5
Bachelor's Degree		23	80	15	27	10	22	5	1
Master's Degree		22	63	5	16	21	14	3	4
Professional/Doctoral Degree		5	24	8	3	6	7	-	-
Religious									
Yes		16	91	23	31	8	20	7	2
No		41	126	13	26	33	38	7	9
No response		1	-	-	-	-	-	-	-

Table S10a-i. Regression results for amount willing to forgo for N=1 comparing altruistic groups to controls (N=275)

N=1	Estimate	SE	95% CI	T	p
Intercept	75.105 ***	2.299	[70.578, 79.632]	32.664	< .0001
Marrow Donor	0.415	3.091	[-5.671, 6.502]	0.134	0.893
Kidney Donor (D)	8.458 **	2.745	[3.053, 13.863]	3.081	0.002
Humanitarian Aid Worker	6.004 *	2.938	[0.220, 11.789]	2.044	0.042
Kidney Donor (ND)	6.315 *	2.800	[0.801, 11.828]	2.255	0.025
Heroic Rescuer	3.645	4.523	[-5.261, 12.550]	0.806	0.421
Liver Donor	7.176	4.728	[-2.133, 16.486]	1.518	0.13
Age	-0.006	0.076	[-0.157, 0.144]	-0.083	0.934
Sex (Female)	0.104	1.946	[-3.728, 3.935]	0.053	0.958

Note. F(8, 266) = 1.825; R^2 = 0.052; Groups are coded as indicator variables relative to the control group. Coefficients are unstandardized. SE indicates the standard error. 95% CI indicates lower/upper limits of the confidence interval. Intercept corresponds to the mean for controls who are not female at average age. * indicates p<.05, ** indicates p<.01, *** indicates p<.001. P-values are two-tailed. Multivariate regressions compared all altruistic groups against controls simultaneously (in lieu of separate tests for which corrections for multiple comparisons would be appropriate).

Table S10a-ii. Regression results for amount willing to forgo for N=1 comparing altruistic groups to controls (N=264)

N=1	Estimate	SE	95% CI	T	p
Intercept	68.778 ***	3.243	[62.392, 75.164]	21.211	< .0001
Marrow Donor	0.907	3.186	[-5.368, 7.182]	0.285	0.776
Kidney Donor (D)	8.826 **	2.849	[3.215, 14.437]	3.098	0.002
Humanitarian Aid Worker	6.46 *	3.038	[0.477, 12.443]	2.126	0.034
Kidney Donor (ND)	7.176 *	2.909	[1.448, 12.904]	2.467	0.014
Heroic Rescuer	5.406	4.759	[-3.966, 14.778]	1.136	0.257
Liver Donor	8.575	5.047	[-1.365, 18.515]	1.699	0.091
Age	-0.02	0.081	[-0.179, 0.139]	-0.253	0.8
Sex (Female)	1.11	2.011	[-2.850, 5.071]	0.552	0.581
College Education	4.171	2.297	[-0.354, 8.695]	1.815	0.071
Income (>$90k)	3.105	1.862	[-0.561, 6.772]	1.668	0.097

Note. F(10, 253) = 2.285; R^2 = 0.083; Groups are coded as indicator variables relative to the control group. Coefficients are unstandardized. SE indicates the standard error. 95% CI indicates lower/upper limits of the confidence interval. Intercept corresponds to the mean for controls who are not female at average age who never completed a four-year degree and household earns <$90,000. * indicates p<.05, ** indicates p<.01, *** indicates p<.001. P-values are two-tailed. Multivariate regressions compared all altruistic groups against controls simultaneously (in lieu of separate tests for which corrections for multiple comparisons would be appropriate).

Table S10b-i. Regression results for amount willing to forgo for N=2 comparing altruistic groups to controls (N=275)

N=2	Estimate	SE	95% CI	T	p
Intercept	74.186 ***	2.705	[68.860, 79.513]	27.424	< .0001
Marrow Donor	2.051	3.637	[-5.109, 9.212]	0.564	0.573
Kidney Donor (D)	5.705	3.230	[-0.654, 12.064]	1.767	0.078
Humanitarian Aid Worker	6.51	3.456	[-0.295, 13.316]	1.883	0.061
Kidney Donor (ND)	6.809 *	3.295	[0.322, 13.296]	2.067	0.04
Heroic Rescuer	2.615	5.321	[-7.862, 13.093]	0.491	0.624
Liver Donor	7.382	5.563	[-3.571, 18.335]	1.327	0.186
Age	0.058	0.090	[-0.119, 0.235]	0.644	0.52
Sex (Female)	-0.026	2.289	[-4.534, 4.482]	-0.011	0.991

Note. F(8, 266) = 1.093; R^2 = 0.032; Groups are coded as indicator variables relative to the control group. Coefficients are unstandardized. SE indicates the standard error. 95% CI indicates lower/upper limits of the confidence interval. Intercept corresponds to the mean for controls who are not female at average age. * indicates p<.05, ** indicates p<.01, *** indicates p<.001. P-values are two-tailed. Multivariate regressions compared all altruistic groups against controls simultaneously (in lieu of separate tests for which corrections for multiple comparisons would be appropriate).

Table S10b-ii. Regression results for amount willing to forgo for N=2 comparing altruistic groups to controls (N=264)

N=2	Estimate	SE	95% CI	T	p
Intercept	71.425 ***	3.860	[63.822, 79.027]	18.503	< .0001
Marrow Donor	2.281	3.793	[-5.190, 9.751]	0.601	0.548
Kidney Donor (D)	6.316	3.392	[-0.363, 12.996]	1.862	0.064
Humanitarian Aid Worker	7.266 *	3.617	[0.144, 14.389]	2.009	0.046
Kidney Donor (ND)	7.586 *	3.463	[0.766, 14.405]	2.191	0.029
Heroic Rescuer	3.058	5.665	[-8.100, 14.215]	0.540	0.59
Liver Donor	7.392	6.009	[-4.441, 19.226]	1.230	0.22
Age	0.022	0.096	[-0.167, 0.212]	0.234	0.815
Sex (Female)	0.376	2.394	[-4.339, 5.090]	0.157	0.875
College Education	-0.038	2.735	[-5.424, 5.348]	-0.014	0.989
Income (>$90k)	3.261	2.216	[-1.104, 7.626]	1.471	0.142

Note. F(10, 253) = 1.153; R^2 = 0.044; Groups are coded as indicator variables relative to the control group. Coefficients are unstandardized. SE indicates the standard error. 95% CI indicates lower/upper limits of the confidence interval. Intercept corresponds to the mean for controls who are not female at average age who never completed a four-year degree and household earns <$90,000. * indicates p<.05, ** indicates p<.01, *** indicates p<.001. P-values are two-tailed. Multivariate regressions compared all altruistic groups against controls simultaneously (in lieu of separate tests for which corrections for multiple comparisons would be appropriate).

Table S10c-i. Regression results for amount willing to forgo for N=5 comparing altruistic groups to controls (N=275)

N=5	Estimate	SE	95% CI	T	p
Intercept	62.543 ***	3.820	[55.023, 70.063]	16.375	< .0001
Marrow Donor	6.015	5.135	[-4.095, 16.125]	1.171	0.243
Kidney Donor (D)	9.808 *	4.560	[0.830, 18.787]	2.151	0.032
Humanitarian Aid Worker	10.946 *	4.880	[1.337, 20.555]	2.243	0.026
Kidney Donor (ND)	10.051 *	4.652	[0.892, 19.210]	2.161	0.032
Heroic Rescuer	5.82	7.513	[-8.974, 20.613]	0.775	0.439
Liver Donor	7.316	7.854	[-8.149, 22.780]	0.931	0.352
Age	0.129	0.127	[-0.121, 0.379]	1.018	0.31
Sex (Female)	2.654	3.232	[-3.711, 9.018]	0.821	0.412

Note. F(8, 266) = 1.465; R^2 = 0.042; Groups are coded as indicator variables relative to the control group. Coefficients are unstandardized. SE indicates the standard error. 95% CI indicates lower/upper limits of the confidence interval. Intercept corresponds to the mean for controls who are not female at average age. * indicates p<.05, ** indicates p<.01, *** indicates p<.001. P-values are two-tailed. Multivariate regressions compared all altruistic groups against controls simultaneously (in lieu of separate tests for which corrections for multiple comparisons would be appropriate).

Table S10c-ii. Regression results for amount willing to forgo for N=5 comparing altruistic groups to controls (N=264)

N=5	Estimate	SE	95% CI	T	p
Intercept	59.607 ***	5.390	[48.992, 70.223]	11.058	< .0001
Marrow Donor	6.381	5.297	[-4.050, 16.813]	1.205	0.229
Kidney Donor (D)	11.216 *	4.736	[1.889, 20.543]	2.368	0.019
Humanitarian Aid Worker	13.123 **	5.050	[3.177, 23.069]	2.598	0.01
Kidney Donor (ND)	11.574 *	4.835	[2.052, 21.097]	2.394	0.017
Heroic Rescuer	5.781	7.911	[-9.799, 21.360]	0.731	0.466
Liver Donor	5.872	8.390	[-10.652, 22.395]	0.700	0.485
Age	0.05	0.134	[-0.215, 0.314]	0.370	0.711
Sex (Female)	3.123	3.343	[-3.460, 9.706]	0.934	0.351
College Education	-3.271	3.819	[-10.792, 4.250]	-0.857	0.392
Income (>$90k)	6.7 *	3.095	[0.605, 12.795]	2.165	0.031

Note. F(10, 253) = 1.866; R^2 = 0.069; Groups are coded as indicator variables relative to the control group. Coefficients are unstandardized. SE indicates the standard error. 95% CI indicates lower/upper limits of the confidence interval. Intercept corresponds to the mean for controls who are not female at average age who never completed a four-year degree and household earns <$90,000. * indicates p<.05, ** indicates p<.01, *** indicates p<.001. P-values are two-tailed. Multivariate regressions compared all altruistic groups against controls simultaneously (in lieu of separate tests for which corrections for multiple comparisons would be appropriate).

Table S10d-i. Regression results for amount willing to forgo for N=10 comparing altruistic groups to controls (N=275)

N=10	Estimate	SE	95% CI	T	p
Intercept	59.965 ***	4.211	[51.674, 68.255]	14.241	< .0001
Marrow Donor	9.454	5.661	[-1.692, 20.600]	1.670	0.096
Kidney Donor (D)	12.931 *	5.027	[3.033, 22.828]	2.572	0.011
Humanitarian Aid Worker	10.466	5.380	[-0.127, 21.058]	1.945	0.053
Kidney Donor (ND)	11.956 *	5.128	[1.859, 22.053]	2.331	0.02
Heroic Rescuer	2.67	8.283	[-13.639, 18.978]	0.322	0.747
Liver Donor	17.37 *	8.659	[0.322, 34.418]	2.006	0.046
Age	0.241	0.140	[-0.034, 0.517]	1.723	0.086
Sex (Female)	-1.925	3.563	[-8.941, 5.091]	-0.540	0.589

Note. $F(8, 266) = 2.036$; $R^2 = 0.058$; Groups are coded as indicator variables relative to the control group. Coefficients are unstandardized. SE indicates the standard error. 95% CI indicates lower/upper limits of the confidence interval. Intercept corresponds to the mean for controls who are not female at average age. * indicates $p<.05$, ** indicates $p<.01$, *** indicates $p<.001$. P-values are two-tailed. Multivariate regressions compared all altruistic groups against controls simultaneously (in lieu of separate tests for which corrections for multiple comparisons would be appropriate).

Table S10d-ii. Regression results for amount willing to forgo for N=10 comparing altruistic groups to controls (N=264)

N=10	Estimate	SE	95% CI	T	p
Intercept	56.289 ***	5.951	[44.569, 68.008]	9.459	< .0001
Marrow Donor	10.344	5.848	[-1.172, 21.860]	1.769	0.078
Kidney Donor (D)	14.722 **	5.229	[4.425, 25.020]	2.816	0.005
Humanitarian Aid Worker	12.696 *	5.576	[1.716, 23.677]	2.277	0.024
Kidney Donor (ND)	13.875 **	5.338	[3.362, 24.388]	2.599	0.01
Heroic Rescuer	6.902	8.734	[-10.298, 24.102]	0.790	0.43
Liver Donor	18.903 *	9.263	[0.660, 37.145]	2.041	0.042
Age	0.175	0.148	[-0.117, 0.467]	1.181	0.239
Sex (Female)	-1.537	3.690	[-8.805, 5.731]	-0.417	0.677
College Education	-1.991	4.216	[-10.295, 6.312]	-0.472	0.637
Income (>$90k)	5.496	3.417	[-1.233, 12.225]	1.609	0.109

Note. $F(10, 253) = 2.099$; $R^2 = 0.077$; Groups are coded as indicator variables relative to the control group. Coefficients are unstandardized. SE indicates the standard error. 95% CI indicates lower/upper limits of the confidence interval. Intercept corresponds to the mean for controls who are not female at average age who never completed a four-year degree and household earns <$90,000. * indicates $p<.05$, ** indicates $p<.01$, *** indicates $p<.001$. P-values are two-tailed. Multivariate regressions compared all altruistic groups against controls simultaneously (in lieu of separate tests for which corrections for multiple comparisons would be appropriate).

Table S10e-i. Regression results for amount willing to forgo for N=20 comparing altruistic groups to controls (N=275)

N=20	Estimate	SE	95% CI	T	p
Intercept	47.979 ***	4.956	[38.221, 57.737]	9.681	< .0001
Marrow Donor	13.327 *	6.663	[0.209, 26.445]	2.000	0.046
Kidney Donor (D)	21.752 ***	5.917	[10.102, 33.401]	3.676	< .0001
Humanitarian Aid Worker	18.438 **	6.332	[5.971, 30.906]	2.912	0.004
Kidney Donor (ND)	16.44 **	6.036	[4.556, 28.323]	2.724	0.007
Heroic Rescuer	17.264	9.749	[-1.930, 36.459]	1.771	0.078
Liver Donor	26.616 **	10.191	[6.551, 46.681]	2.612	0.01
Age	0.242	0.165	[-0.082, 0.567]	1.471	0.142
Sex (Female)	-4.505	4.194	[-12.763, 3.753]	-1.074	0.284

Note. F(8, 266) = 3.088; R^2 = 0.085; Groups are coded as indicator variables relative to the control group. Coefficients are unstandardized. SE indicates the standard error. 95% CI indicates lower/upper limits of the confidence interval. Intercept corresponds to the mean for controls who are not female at average age. * indicates p<.05, ** indicates p<.01, *** indicates p<.001. P-values are two-tailed. Multivariate regressions compared all altruistic groups against controls simultaneously (in lieu of separate tests for which corrections for multiple comparisons would be appropriate).

Table S10e-ii. Regression results for amount willing to forgo for N=20 comparing altruistic groups to controls (N=264)

N=20	Estimate	SE	95% CI	T	p
Intercept	40.437 ***	6.883	[26.883, 53.992]	5.875	< .0001
Marrow Donor	15.112 *	6.763	[1.793, 28.431]	2.234	0.026
Kidney Donor (D)	24.466 ***	6.047	[12.557, 36.375]	4.046	< .0001
Humanitarian Aid Worker	21.814 ***	6.449	[9.114, 34.514]	3.383	0.001
Kidney Donor (ND)	20.573 ***	6.174	[8.415, 32.732]	3.332	0.001
Heroic Rescuer	23.455 *	10.101	[3.563, 43.348]	2.322	0.021
Liver Donor	28.631 **	10.713	[7.532, 49.729]	2.672	0.008
Age	0.156	0.171	[-0.182, 0.493]	0.908	0.365
Sex (Female)	-3.362	4.268	[-11.767, 5.044]	-0.788	0.432
College Education	-1.751	4.876	[-11.354, 7.853]	-0.359	0.72
Income (>$90k)	9.099 *	3.952	[1.317, 16.882]	2.303	0.022

Note. F(10, 253) = 3.668; R^2 = 0.127; Groups are coded as indicator variables relative to the control group. Coefficients are unstandardized. SE indicates the standard error. 95% CI indicates lower/upper limits of the confidence interval. Intercept corresponds to the mean for controls who are not female at average age who never completed a four-year degree and household earns <$90,000. * indicates p<.05, ** indicates p<.01, *** indicates p<.001. P-values are two-tailed. Multivariate regressions compared all altruistic groups against controls simultaneously (in lieu of separate tests for which corrections for multiple comparisons would be appropriate).

Table S10f-i. Regression results for amount willing to forgo for N=50 comparing altruistic groups to controls (N=275)

N=50	Estimate	SE	95% CI	T	p
Intercept	44.914 ***	5.351	[34.378, 55.450]	8.393	< .0001
Marrow Donor	-0.4	7.194	[-14.564, 13.765]	-0.056	0.956
Kidney Donor (D)	9.345	6.389	[-3.234, 21.924]	1.463	0.145
Humanitarian Aid Worker	7.724	6.837	[-5.738, 21.186]	1.130	0.26
Kidney Donor (ND)	14.198 *	6.517	[1.366, 27.030]	2.179	0.03
Heroic Rescuer	4.499	10.526	[-16.227, 25.225]	0.427	0.669
Liver Donor	24.497 *	11.004	[2.831, 46.163]	2.226	0.027
Age	0.169	0.178	[-0.181, 0.519]	0.949	0.344
Sex (Female)	-4.899	4.529	[-13.816, 4.017]	-1.082	0.28

Note. F(8, 266) = 1.732; R^2 = 0.050; Groups are coded as indicator variables relative to the control group. Coefficients are unstandardized. SE indicates the standard error. 95% CI indicates lower/upper limits of the confidence interval. Intercept corresponds to the mean for controls who are not female at average age. * indicates p<.05, ** indicates p<.01, *** indicates p<.001. P-values are two-tailed. Multivariate regressions compared all altruistic groups against controls simultaneously (in lieu of separate tests for which corrections for multiple comparisons would be appropriate).

Table S10f-ii. Regression results for amount willing to forgo for N=50 comparing altruistic groups to controls (N=264)

N=50	Estimate	SE	95% CI	T	p
Intercept	47.086 ***	7.383	[32.546, 61.626]	6.378	< .0001
Marrow Donor	-0.962	7.255	[-15.249, 13.326]	-0.133	0.895
Kidney Donor (D)	10.977	6.487	[-1.799, 23.752]	1.692	0.092
Humanitarian Aid Worker	10.367	6.917	[-3.256, 23.990]	1.499	0.135
Kidney Donor (ND)	16.811 *	6.623	[3.768, 29.854]	2.538	0.012
Heroic Rescuer	7.884	10.835	[-13.455, 29.223]	0.728	0.468
Liver Donor	23.217 *	11.492	[0.585, 45.849]	2.020	0.044
Age	0.06	0.184	[-0.302, 0.422]	0.326	0.745
Sex (Female)	-5.682	4.578	[-14.698, 3.335]	-1.241	0.216
College Education	-10.025	5.231	[-20.326, 0.277]	-1.916	0.056
Income (>$90k)	8.008	4.239	[-0.340, 16.356]	1.889	0.06

Note. F(10, 253) = 2.469; R^2 = 0.089; Groups are coded as indicator variables relative to the control group. Coefficients are unstandardized. SE indicates the standard error. 95% CI indicates lower/upper limits of the confidence interval. Intercept corresponds to the mean for controls who are not female at average age who never completed a four-year degree and household earns <$90,000. * indicates p<.05, ** indicates p<.01, *** indicates p<.001. P-values are two-tailed. Multivariate regressions compared all altruistic groups against controls simultaneously (in lieu of separate tests for which corrections for multiple comparisons would be appropriate).

Table S10g-i. Regression results for amount willing to forgo for N=100 comparing altruistic groups to controls (N=275)

	**Estimate**	**SE**	**95% CI**	**T**	**p**
Intercept	30.859 ***	5.387	[20.252, 41.466]	5.728	< .0001
Marrow Donor	11.035	7.243	[-3.225, 25.295]	1.524	0.129
Kidney Donor (D)	10.393	6.432	[-2.271, 23.056]	1.616	0.107
Humanitarian Aid Worker	19.473 **	6.883	[5.920, 33.026]	2.829	0.005
Kidney Donor (ND)	21.092 **	6.561	[8.174, 34.011]	3.215	0.001
Heroic Rescuer	13.154	10.597	[-7.711, 34.020]	1.241	0.216
Liver Donor	30.092 **	11.078	[8.279, 51.904]	2.716	0.007
Age	0.157	0.179	[-0.196, 0.509]	0.875	0.382
Sex (Female)	-5.515	4.559	[-14.492, 3.462]	-1.210	0.227

Note. F(8, 266) = 2.622; R^2 = 0.073; Groups are coded as indicator variables relative to the control group. Coefficients are unstandardized. SE indicates the standard error. 95% CI indicates lower/upper limits of the confidence interval. Intercept corresponds to the mean for controls who are not female at average age. * indicates p<.05, ** indicates p<.01, *** indicates p<.001. P-values are two-tailed. Multivariate regressions compared all altruistic groups against controls simultaneously (in lieu of separate tests for which corrections for multiple comparisons would be appropriate).

Table S10g-ii. Regression results for amount willing to forgo for N=100 comparing altruistic groups to controls (N=264)

	**Estimate**	**SE**	**95% CI**	**T**	**p**
Intercept	30.375 ***	7.494	[15.617, 45.134]	4.053	< .0001
Marrow Donor	10.05	7.364	[-4.452, 24.553]	1.365	0.174
Kidney Donor (D)	11.457	6.585	[-1.510, 24.425]	1.740	0.083
Humanitarian Aid Worker	20.249 **	7.022	[6.421, 34.077]	2.884	0.004
Kidney Donor (ND)	21.5 **	6.722	[8.261, 34.739]	3.198	0.002
Heroic Rescuer	16.276	10.998	[-5.384, 37.936]	1.480	0.14
Liver Donor	27.995 *	11.665	[5.022, 50.968]	2.400	0.017
Age	0.145	0.187	[-0.223, 0.512]	0.775	0.439
Sex (Female)	-4.859	4.647	[-14.012, 4.293]	-1.046	0.297
College Education	-5.857	5.310	[-16.314, 4.600]	-1.103	0.271
Income (>$90k)	7.478	4.303	[-0.996, 15.952]	1.738	0.083

Note. F(10, 253) = 2.722; R^2 = 0.097; Groups are coded as indicator variables relative to the control group. Coefficients are unstandardized. SE indicates the standard error. 95% CI indicates lower/upper limits of the confidence interval. Intercept corresponds to the mean for controls who are not female at average age who never completed a four-year degree and household earns <$90,000. * indicates p<.05, ** indicates p<.01, *** indicates p<.001. P-values are two-tailed. Multivariate regressions compared all altruistic groups against controls simultaneously (in lieu of separate tests for which corrections for multiple comparisons would be appropriate).

Table S11. Hyperbolic mixed-effects model results for group differences in social discounting

	Estimate	SE	95% CI	T	p
**Level 1**					
v0	78.605 ***	0.877	[76.884, 80.326]	89.605	< .0001
Intercept	2.35 **	0.754	[0.872, 3.828]	3.119	0.002
**Level 2**					
Heroic Rescuer	-0.736	3.403	[-7.411, 5.940]	-0.216	0.829
Humanitarian Aid Worker	0.013	1.278	[-2.494, 2.520]	0.010	0.992
Kidney Donor (D)	-5.682 ***	1.025	[-7.692, -3.672]	-5.545	< .0001
Kidney Donor (ND)	-6.408 ***	1.203	[-8.768, -4.048]	-5.326	< .0001
Liver Donor	-9.954 **	3.396	[-16.614, -3.293]	-2.931	0.003
Marrow Donor	-0.65	1.182	[-2.969, 1.670]	-0.549	0.583
Honesty Humility	-1.646 ***	0.201	[-2.040, -1.252]	-8.195	< .0001
Sex (Female)	0.508 ***	0.133	[0.246, 0.770]	3.807	< .0001
Age	-0.003	0.005	[-0.014, 0.007]	-0.660	0.509
College Education	0.562 ***	0.155	[0.258, 0.866]	3.629	< .0001
Income (≥$90k)	-0.604 ***	0.114	[-0.828, -0.380]	-5.294	< .0001
Heroic Rescuer x Honesty Humility	0.075	0.874	[-1.640, 1.789]	0.085	0.932
Humanitarian Aid Worker x Honesty Humility	-0.246	0.345	[-0.923, 0.431]	-0.713	0.476
Kidney Donor (D) x Honesty Humility	1.245 ***	0.267	[0.721, 1.770]	4.661	< .0001
Kidney Donor (ND) x Honesty Humility	1.376 ***	0.307	[0.774, 1.979]	4.479	< .0001
Liver Donor x Honesty Humility	2.129 **	0.812	[0.537, 3.722]	2.623	0.009
Marrow Donor x Honesty Humility	0.113	0.311	[-0.498, 0.724]	0.362	0.717

Note. Groups are coded as indicator variables relative to the control group. Fixed-effects coefficients are unstandardized. SE indicates the standard error. 95% CI indicates lower/upper limits of the confidence interval. * indicates p<.05, ** indicates p<.01, *** indicates p<.001. P-values are two-tailed. Multivariate mixed-effects regressions compared all altruistic groups against controls simultaneously (in lieu of separate tests for which corrections for multiple comparisons would be appropriate).

Table S12. Table of demographics of social discounting sub-sample (for classification analysis)

		**Control**	**Combined Altruists**	**Bone Marrow Donor**	**Kidney Donor (D)**	**Humanitarian Aid Worker**	**Kidney Donor (ND)**	**Heroic Rescuer**	**Liver Donor**
N = 264		53	211	35	56	40	57	13	10
Sex	F : M	35 : 18	133 : 78	17 : 18	45 : 11	28 : 12	36 : 21	0 : 13	7 : 3
Age	M (SE)	38.26 (1.25)	44.18 (.89)	35.49 (1.88)	45.73 (1.68)	41.25 (1.75)	50.05 (1.71)	47.54 (3.74)	39.80 (1.91)
Household Income									
Under $9,999		1	5	3	1	-	1	-	-
$10,000-14,999		1	1	-	-	1	-	-	-
$15,000-24,999		-	6	2	-	3	-	-	1
$25,000-39,999		2	15	1	4	1	8	1	-
$40,000-59,999		6	27	5	6	7	7	1	1
$60,000-89,999		17	35	4	12	8	6	3	2
$90,000-179,999		21	84	17	26	12	20	3	6
Over $180,000		5	38	3	7	8	15	5	-
Don't know / No response		-	-	-	-	-	-	-	-
Race									
White		38	181	22	54	32	52	12	9
African American / Black		6	5	4	-	-	1	-	-
Asian / Pacific Islander		4	10	3	1	3	3	-	-
Latino		-	4	2	-	1	-	-	1
Native American/Alaskan		-	1	1	-	1	-	-	-
Multiracial		4	4	2	-	-	1	1	-
Other		1	5	1	1	3	-	-	-
Prefer not to Respond		-	-	-	-	-	-	-	-
Hispanic									
Yes		-	4	2	-	1	-	-	1
No		53	207	33	56	39	57	13	9
Education				2	2	-	3	2	1
High School		2	10	6	9	4	11	4	4
Some College		6	38	14	27	10	22	4	1
Bachelor's Degree		20	78	5	15	20	14	3	4
Master's Degree		20	61	8	3	6	7	-	-
Professional/Doctoral Degree		5	24	-	-	-	-	-	-
Prefer not to Respond		-	-						
Religious	Yes	14	86	22	30	8	19	6	1
No		39	125	13	26	32	38	7	9

Table S13. Means and standard deviations of trait and behavioral measures among altruists and controls

	**Controls**	**Combined Altruists**	**Bone Marrow Donors**	**Kidney Donors (D)**	**Humanitarian Aid Workers**	**Kidney Donors (ND)**	**Heroic Rescuers**	**Liver Donors**
**HEXACO**	**N=206**	**N=347**	**N=55**	**N=68**	**N=53**	**N=132**	**N=27**	**N=12**
Honesty-Humility	3.68 (.63)	4.04 (.57)	3.94 (.62)	4.06 (.63)	3.94 (.57)	4.12 (.54)	4.05 (.40)	3.92 (.62)
Emotionality	3.06 (.70)	2.94 (.65)	2.91 (.63)	3.17 (.62)	2.93 (.61)	2.93 (.68)	2.44 (.39)	2.97 (.60)
Extraversion	3.46 (.68)	3.54 (.59)	3.40 (.62)	3.47 (.60)	3.63 (.50)	3.64 (.57)	3.46 (.69)	3.28 (.48)
Agreeableness	3.25 (.64)	3.37 (.61)	3.41 (.54)	3.25 (.67)	3.22 (.51)	3.53 (.61)	3.15 (.52)	3.21 (.55)
Conscientiousness	3.84 (.50)	3.90 (.49)	3.96 (.53)	3.93 (.47)	3.88 (.45)	3.87 (.52)	3.80 (.51)	4.00 (.34)
Openness	3.79 (.59)	3.67 (.61)	3.62 (.65)	3.52 (.59)	3.83 (.55)	3.75 (.59)	3.52 (.80)	3.52 (.75)
**DOSPERT**	**N=207**	**N=347**	**N=55**	**N=68**	**N=53**	**N=132**	**N=27**	**N=12**
Social	5.44 (.93)	5.34 (.97)	5.06 (.85)	5.09 (.93)	5.90 (.81)	5.34 (1.04)	5.38 (.80)	5.38 (.97)
Ethical	2.13 (.97)	1.81 (.79)	1.91 (.92)	1.85 (.75)	2.07 (.85)	1.67 (.68)	1.75 (.51)	1.71 (.76)
Financial	2.64 (.92)	2.69 (1.04)	2.81 (.99)	2.47 (.72)	2.83 (1.09)	2.64 (1.09)	3.07 (1.41)	2.49 (.72)
Health/Safety	2.91 (1.27)	2.72 (1.14)	2.73 (1.04)	2.41 (1.05)	2.95 (1.14)	2.67 (1.18)	3.46 (1.13)	2.49 (1.05)
Recreational	3.38 (1.68)	3.58 (1.67)	3.55 (1.62)	2.92 (1.48)	4.00 (1.72)	3.59 (1.72)	4.32 (1.40)	3.81 (1.68)
**IRI**	**N=207**	**N=347**	**N=55**	**N=68**	**N=53**	**N=132**	**N=27**	**N=12**
Personal Distress	16.79 (5.35)	14.71 (5.01)	15.56 (4.54)	15.22 (5.46)	13.55 (4.53)	15.17 (5.23)	11.70 (3.81)	14.75 (3.55)
Empathic Concern	27.59 (5.30)	29.13 (4.60)	27.02 (4.99)	29.10 (4.78)	29.47 (3.85)	30.28 (4.14)	26.67 (4.33)	29.08 (5.26)
Perspective-Taking	26.45 (4.91)	26.88 (4.51)	26.29 (4.37)	26.32 (5.09)	27.32 (4.01)	27.73 (4.15)	24.70 (4.33)	26.33 (6.08)
Fantasy	23.96 (5.48)	23.67 (5.63)	22.98 (5.47)	23.29 (6.12)	23.66 (5.95)	24.41 (5.26)	21.67 (5.65)	25.42 (5.26)
**PPI**	**N=207**	**N=347**	**N=55**	**N=68**	**N=53**	**N=132**	**N=27**	**N=12**
Self-centered Impulsivity	48.86 (10.62)	47.35 (9.50)	45.76 (10.32)	46.71 (10.33)	51.49 (9.26)	46.06 (8.76)	49.04 (7.05)	50.50 (9.47)
Coldheartedness	14.21 (3.62)	13.17 (3.66)	14.47 (4.08)	13.31 (3.67)	12.79 (3.30)	12.36 (3.42)	14.96 (3.02)	13.00 (4.43)
Fearless Dominance	51.15 (10.68)	53.51 (9.64)	51.40 (9.99)	49.69 (8.29)	54.57 (9.86)	54.61 (9.68)	59.56 (7.17)	54.42 (9.84)
Cognitive Reflection	**N=207**<br>.38 (.49)	**N=299**<br>.40 (.49)	**N=55**<br>.51 (.50)	**N=68**<br>.34 (.48)	**N=53**<br>.36 (.48)	**N=87**<br>.40 (.49)	**N=27**<br>.46 (.51)	**N=12**<br>.42 (.51)
Social Discounting (logk)	**N=58**<br>-3.36 (2.29)	**N=217**<br>-4.44 (1.96)	**N=36**<br>-3.93 (2.30)	**N=57**<br>-4.36 (1.77)	**N=41**<br>-4.46 (1.89)	**N=58**<br>-4.75 (1.96)	**N=14**<br>-4.34 (2.28)	**N=11**<br>-5.05 (1.50)

Table S14. Table of bivariate Spearman rho correlations among variables of interest.

	1	2	3	4	5	6	7	8	9	10	11	12	13	14	15	16	17	18	19
**HEXACO (N=553)**																			
(1) Honesty-Humility																			
(2) Emotionality	-.05																		
(3) Extraversion	.1	-.17																	
(4) Agreeableness	.32	-.21	.24																
(5) Conscientiousness	.13	.11	.05	-.17															
(6) Openness	.11	-.09	.09	.15	.02														
**DOSPERT**																			
(7) Social	.12	-.2	.21	-.09	.02	.36													
(8) Ethical	-.46	-.03	-.04	-.26	-.17	-.14	.06												
(9) Financial	-.36	-.14	.07	-.04	-.08	.1	.21	.23											
(10) Health/Safety	-.2	-.22	-.01	-.1	-.26	-.06	.27	.47	.25										
(11) Recreational	-.04	-.4	.1	.09	-.2	.15	.36	.16	.3	.45									
**IRI**																			
(12) Personal Distress	-.23	.57	-.35	-.16	-.11	-.17	-.34	.06	-.07	-.13	-.3								
(13) Empathic Concern	.36	.31	.26	.3	.18	.16	.14	-.24	-.19	-.27	-.12	.01							
(14) Perspective-Taking	.28	.001	.24	.48	.16	.26	.15	-.2	-.03	-.15	.03	-.13	.54						
(15) Fantasy	.03	.34	-.03	.1	.05	.33	.1	-.06	.02	-.11	-.02	.15	.37	.26					
**PPI**																			
(16) Self-centered Impulsivity	-.35	-.08	-.07	-.24	-.41	.1	.19	.39	.23	.37	.33	.04	-.19	-.2	.08				
(17) Coldheartedness	-.28	-.39	-.08	-.2	-.11	-.13	-.08	.31	.21	.27	.15	-.21	-.64	-.43	-.37	.14			
(18) Fearless Dominance	.04	-.58	.51	.21	-.1	.22	.37	.03	.24	.33	.67	-.51	-.02	.11	-.09	.26	.15		
(19) Social Discounting Rate	-.29	.08	-.1	-.19	-.12	-.14	-.07	.2	.08	.16	-.01	.16	-.21	-.21	-.05	.18	.26	-.08	

Table S15. Table of demographics of exploratory perceptions samples

		Study 2a (Exploratory)	Study 2b (Confirmatory)
N		208	201
Sex	Female : Male : Other	104 : 100 : 4	101 : 99 : 1
Age	Mean (SE)	44.03 (1.02)	46.32 (1.21)
18-34 years		63	62
35-54 years		71	68
55-65 years		74	71
Race			
White (Non-Hispanic)		140	121
African American / Black (Non-Hispanic)		27	25
Asian / Pacific Islander		11	10
Latino		24	34
Multiracial		2	7
Native American / American Indian		1	1
Other		1	1
Prefer not to Respond		2	2
Hispanic			
Yes		24	34
No		184	167
Education			
Did not complete High School		6	3
High School		39	45
Some College		31	43
Bachelor's Degree		83	53
Master's Degree		36	52
Professional/Doctoral Degree		13	5

Table S16a. *Definitions of groups included in the independent study of altruistic perceptions*

Group	Definition
Bone Marrow Donor	A bone marrow donor is a person who donates bone marrow to a stranger who has a serious blood disease like leukemia. Donation entails either having marrow extracted with a needle from the donor's pelvis during surgery, or having stem cells extracted from the donor's blood. Donors cannot receive any payment for donating. After the procedure, donors typically recover in 1-20 days.
Heroic Rescuer	A heroic rescuer is a person who voluntarily risks his or her life to an extraordinary degree while saving or attempting to save the life of another person (who is often a stranger). A heroic rescuer physically rescues people from a life-threatening situation such as drowning, fire, or vehicular accidents. A heroic rescuer is NOT a first responder, so they have no obligation to attempt the rescue.
Humanitarian Aid Worker	A humanitarian aid worker is a person who provides help and emergency relief to people around the world. They may help in situations such as natural disasters, displacement due to conflict or helping populations that require medical outreach. A humanitarian mission may last for weeks or months, and aid workers may receive pay or work as unpaid volunteers.
Kidney Donor (Directed)	A directed kidney donor is a person who undergoes surgery to give one of their kidneys to a specific person they know whose kidneys have failed. The recipient is most often a close family member or friend. People can typically lead normal lives with a single kidney. Donors cannot receive any pay or personal benefits for donating. After the surgery, donors typically recover in 4-6 weeks.
Kidney Donor (Non-directed)	A non-directed kidney donor is a person who undergoes surgery to give one of their kidneys to an anonymous stranger whose kidneys have failed. Donors can typically live normal lives with a single kidney. Donors do not meet their recipient prior to the donation, and may or may not ever meet them afterward. Donors cannot receive any pay or personal benefits for donating. After the surgery, donors typically recover in 4-6 weeks.
Liver Donor	A liver donor is a person who undergoes surgery to give part of their liver to someone (a specific person they know or a stranger) whose own liver has failed. This procedure is possible because of the liver's unique ability to regenerate which takes 2-4 months. After the surgery, donors typically recover in 6-8 weeks.
Internet Troll	An internet troll is a person who purposely upsets people on the internet in an effort to create discord. A troll often achieves this by posting inflammatory messages in an online community with the intent of provoking readers into displaying emotional responses whether for the troll's amusement or a specific gain.
Marathon Runner	A marathon runner is a person who participates in long-distance footraces of up to 26 miles. Marathon runners have to go through extensive physical and mental training to be in healthy condition for the race. Marathon races take place on either a road or in cross country settings of similar distances.

Tax Evader	A tax evader is a person who deliberately participates in the illegal avoidance of paying proper taxes or the concealment of reportable income. This is a federal offense for which tax evaders are subject to criminal charges such as jail time and substantial monetary penalties.
Blood Donor (2a only)	A blood donor is a person who donates blood often in the context of a blood drive. A person must be 17 years old and at least 110 lbs to donate blood. Whole blood is the most flexible form of donation as it can be used to help various recipients when used on its own or separated into red cells, platelets, and plasma.
Foster Parent (2a only)	A foster parent is a person who brings a child into their family for a period of time, without legally adopting the child. Foster parents must provide a safe and comfortable family environment for the child, and care for the child's basic physical and emotional needs. A foster parent must undergo a certification process. Often times, a foster parent will receive a monetary reimbursement based on the needs of the child.
Mountaineer (2a only)	A mountaineer is a person who climbs mountains using various techniques such as hiking, rock climbing, or skiing oftentimes in difficult terrains. A mountaineer has knowledge of safety and proper technique, and emergency protocols.
Analog Astronaut (2b only)	An analog astronaut is a person who tests features of spaceflight missions and operations, such as robotics in emergency procedures or aspects of habitability, in order to identify and solve potential problems that could result in fatalities before launch. The tests they conduct are often set in isolated, confined and extreme environments, to better recreate the harsh extra-terrestrial conditions.
BASE Jumper (2b only)	A BASE jumper is someone who jumps off tall structures like buildings, cliffs, and bridges using only a single parachute for safety. BASE jumping is significantly more hazardous than parachuting from planes because of the risk of striking objects and the limited time available to correct any errors. It is considered to be one of the most dangerous extreme sports, and is legally prohibited in many areas.
Miss America Contestant (2b only)	A Miss America contestant is a person who previously competed in the annual Miss America pageant when she was between the ages of 17-25. In this pageant, 50 women representing each of the 50 U.S. states compete. Contestants must first enter and win a local competition and then compete again to represent their state in the national competition. During the pageant, contestants are judged on their answers to interview questions and their talent performances, such as singing, dancing, or playing a musical instrument. Historically, the pageant also featured a swimsuit competition in which contestants' physical appearance was evaluated by judges.
The Average (Typical) Person	An average person is someone whose characteristics represent a majority of the population.

Table S16b. *Definitions of characteristics included in the independent study of altruistic perceptions*

Group	Definition
Altruism	Altruism is behavior aimed at improving another individual's well-being and which may be risky and/or costly for the altruist. Please rate the degree to which you consider the behavior of the following individuals to be altruistic.
Risk	Risk involves activities that may be physically dangerous for the person doing them. Please rate the degree to which you consider the behavior of the following individuals to be risky.
Honesty-Humility	Honesty-humility is a personality trait. People with high honesty-humility scores do not feel entitled to a higher social status, avoid using others for personal gain, avoid breaking the rules, and avoid obtaining lavish wealth and luxuries. Conversely, people with low honesty-humility scores feel a strong sense of self-importance, seek out opportunities to get what they want from others through flattery, break the rules for personal gain, and find motivation through material achievement. Please indicate where you think the following individuals would score on a scale of honesty-humility.
Emotionality	Emotionality is a personality trait. People with high emotionality scores experience fear of physical dangers, anxiety in response to life's stresses, a need for emotional support from others, and empathy and sentimental attachments for others. Conversely, people with low emotionality scores experience little fear of physical harm, little worry even in stressful situations, little need to share their concerns with others, and emotional detachment from others. Please indicate where you think the following individuals would score on a scale of emotionality.
Extraversion	Extraversion is a personality trait. People with high extraversion scores feel positive about themselves, feel confident when leading or addressing groups of people, enjoy social gatherings and interactions, and experience positive feelings of enthusiasm and energy. Conversely, people with low scores extraversion consider themselves unpopular, feel awkward when they are the center of social attention, have little to no interest in social activities, and feel less lively and optimistic than others do. Please indicate where you think the following individuals would score on a scale of extraversion.
Agreeableness	Agreeableness is a personality trait. People with high agreeableness scores are more likely to compromise, cooperate, and be accepting of others. People with low agreeableness scores are more likely to have a pessimistic view of human nature, hold grudges, expect the worst from people, and be easily angered. Please indicate where you think the following individuals would score on a scale of agreeableness.
Conscientiousness	Conscientiousness is a personality trait. People with high conscientiousness scores are organized, work strategically toward their goals, strive for accuracy and perfection, and make decisions rationally. People with a low level of conscientiousness scores are unconcerned with the organization of their physical surroundings, avoid difficult tasks or challenging goals, are satisfied with work that contains errors, and make decisions on impulse. Please indicate where you think the following individuals would score on a scale of conscientiousness.
Openness to Experience	Openness is a personality trait. People with high openness scores are interested in the beauty and art of nature, are curious about various intellectual subjects, and use their imagination in their everyday life. People with low openness scores are unimpressed by most works of art, feel little intellectual curiosity, avoid creative pursuits, and feel little attraction toward ideas that may seem radical or unconventional. Please indicate where you think the following individuals would score on a scale of openness.

Table S17a-i. Linear mixed-effects results for beliefs about altruistic groups' Altruism (Study 2a)

**Altruism**	**Estimate**	**SE**	**95% CI**	**T**	**p**
Intercept	3.144 ***	0.078	[2.991, 3.298]	40.187	< .0001
Marrow Donor	0.87 ***	0.084	[0.705, 1.035]	10.363	< .0001
Kidney Donor (D)	0.957 ***	0.084	[0.792, 1.121]	11.393	< .0001
Humanitarian Aid Worker	0.894 ***	0.084	[0.729, 1.059]	10.649	< .0001
Kidney Donor (ND)	0.832 ***	0.084	[0.667, 0.996]	9.905	< .0001
Heroic Rescuer	1.101 ***	0.084	[0.936, 1.266]	13.111	< .0001
Liver Donor	0.909 ***	0.084	[0.744, 1.073]	10.821	< .0001

Note. Groups are coded as indicator variables relative to the average person. Fixed-effects coefficients are unstandardized. SE indicates the standard error. 95% CI indicates lower/upper limits of the confidence interval. * indicates p<.05, ** indicates p<.01, *** indicates p<.001. P-values are two-tailed. Multivariate mixed-effects regressions compared ratings of all altruistic groups against ratings of the average person simultaneously (in lieu of separate tests for which corrections for multiple comparisons would be appropriate).

Table S17a-ii. Linear mixed-effects results for beliefs about altruistic groups' Altruism (Study 2b)

**Altruism**	**Estimate**	**SE**	**95% CI**	**T**	**p**
Intercept	2.701 ***	0.078	[2.548, 2.855]	34.580	< .0001
Marrow Donor	1.567 ***	0.082	[1.406, 1.729]	19.053	< .0001
Kidney Donor (D)	1.488 ***	0.082	[1.326, 1.649]	18.086	< .0001
Humanitarian Aid Worker	1.358 ***	0.082	[1.197, 1.520]	16.513	< .0001
Kidney Donor (ND)	1.488 ***	0.082	[1.326, 1.649]	18.086	< .0001
Heroic Rescuer	1.622 ***	0.082	[1.461, 1.783]	19.719	< .0001
Liver Donor	1.522 ***	0.082	[1.361, 1.684]	18.509	< .0001

Note. Groups are coded as indicator variables relative to the average person. Fixed-effects coefficients are unstandardized. SE indicates the standard error. 95% CI indicates lower/upper limits of the confidence interval. * indicates p<.05, ** indicates p<.01, *** indicates p<.001. P-values are two-tailed. Multivariate mixed-effects regressions compared ratings of all altruistic groups against ratings of the average person simultaneously (in lieu of separate tests for which corrections for multiple comparisons would be appropriate).

Table S17b-i. Linear mixed-effects results for beliefs about altruistic groups' Risk (Study 2a)

Risk	Estimate	SE	95% CI	T	p
Intercept	2.764 ***	0.074	[2.619, 2.910]	37.374	< .0001
Marrow Donor	1.221 ***	0.086	[1.053, 1.390]	14.215	< .0001
Kidney Donor (D)	1.245 ***	0.086	[1.077, 1.414]	14.495	< .0001
Humanitarian Aid Worker	0.923 ***	0.086	[0.755, 1.092]	10.745	< .0001
Kidney Donor (ND)	1.183 ***	0.086	[1.014, 1.351]	13.768	< .0001
Heroic Rescuer	1.625 ***	0.086	[1.456, 1.794]	18.916	< .0001
Liver Donor	1.245 ***	0.086	[1.077, 1.414]	14.495	< .0001

Note. Groups are coded as indicator variables relative to the average person. Fixed-effects coefficients are unstandardized. SE indicates the standard error. 95% CI indicates lower/upper limits of the confidence interval. * indicates p<.05, ** indicates p<.01, *** indicates p<.001. P-values are two-tailed. Multivariate mixed-effects regressions compared ratings of all altruistic groups against ratings of the average person simultaneously (in lieu of separate tests for which corrections for multiple comparisons would be appropriate).

Table S17b-ii. Linear mixed-effects results for beliefs about altruistic groups' Risk (Study 2b)

Risk	Estimate	SE	95% CI	T	p
Intercept	2.368 ***	0.080	[2.212, 2.525]	29.717	< .0001
Marrow Donor	1.348 ***	0.088	[1.175, 1.522]	15.240	< .0001
Kidney Donor (D)	1.502 ***	0.088	[1.329, 1.676]	16.983	< .0001
Humanitarian Aid Worker	1.119 ***	0.088	[0.946, 1.293]	12.653	< .0001
Kidney Donor (ND)	1.413 ***	0.088	[1.239, 1.587]	15.971	< .0001
Heroic Rescuer	1.965 ***	0.088	[1.792, 2.139]	22.213	< .0001
Liver Donor	1.468 ***	0.088	[1.294, 1.641]	16.589	< .0001

Note. Groups are coded as indicator variables relative to the average person. Fixed-effects coefficients are unstandardized. SE indicates the standard error. 95% CI indicates lower/upper limits of the confidence interval. * indicates p<.05, ** indicates p<.01, *** indicates p<.001. P-values are two-tailed. Multivariate mixed-effects regressions compared ratings of all altruistic groups against ratings of the average person simultaneously (in lieu of separate tests for which corrections for multiple comparisons would be appropriate).

Table S17c-i. Linear mixed-effects results for beliefs about altruistic groups' Social Discounting (Study 2a)

Social Discounting	Estimate	SE	95% CI	T	p
Intercept	-4.967 ***	0.174	[-5.308, -4.626]	-28.579	< .0001
Heroic Rescuer	-1.908 ***	0.160	[-2.223, -1.593]	-11.890	< .0001
Humanitarian Aid Worker	-1.514 ***	0.160	[-1.829, -1.200]	-9.437	< .0001
Kidney Donor (D)	-0.866 ***	0.160	[-1.181, -0.551]	-5.395	< .0001
Kidney Donor (ND)	-1.412 ***	0.160	[-1.727, -1.097]	-8.798	< .0001
Liver Donor	-1.213 ***	0.160	[-1.528, -0.898]	-7.559	< .0001
Marrow Donor	-1.104 ***	0.160	[-1.419, -0.789]	-6.880	< .0001

Note. Groups are coded as indicator variables relative to the average person. Fixed-effects coefficients are unstandardized. SE indicates the standard error. 95% CI indicates lower/upper limits of the confidence interval. * indicates p<.05, ** indicates p<.01, *** indicates p<.001. P-values are two-tailed. Multivariate mixed-effects regressions compared ratings of all altruistic groups against ratings of the average person simultaneously (in lieu of separate tests for which corrections for multiple comparisons would be appropriate).

Table S17c-ii. Linear mixed-effects results for beliefs about altruistic groups' Social Discounting (Study 2b)

Social Discounting	Estimate	SE	95% CI	T	p
Intercept	-4.504 ***	0.166	[-4.830, -4.179]	-27.150	< .0001
Heroic Rescuer	-1.87 ***	0.152	[-2.168, -1.571]	-12.301	< .0001
Humanitarian Aid Worker	-1.931 ***	0.152	[-2.229, -1.633]	-12.706	< .0001
Kidney Donor (D)	-1.069 ***	0.152	[-1.368, -0.771]	-7.036	< .0001
Kidney Donor (ND)	-1.438 ***	0.152	[-1.736, -1.140]	-9.461	< .0001
Liver Donor	-1.342 ***	0.152	[-1.640, -1.044]	-8.832	< .0001
Marrow Donor	-1.376 ***	0.152	[-1.674, -1.078]	-9.054	< .0001

Note. Groups are coded as indicator variables relative to the average person. Fixed-effects coefficients are unstandardized. SE indicates the standard error. 95% CI indicates lower/upper limits of the confidence interval. * indicates p<.05, ** indicates p<.01, *** indicates p<.001. P-values are two-tailed. Multivariate mixed-effects regressions compared ratings of all altruistic groups against ratings of the average person simultaneously (in lieu of separate tests for which corrections for multiple comparisons would be appropriate).

Table S17d-i. Linear mixed-effects results for beliefs about altruistic groups' Honesty-Humility (Study 2a)

Honesty-Humility	Estimate	SE	95% CI	T	p
Intercept	3.462 ***	0.080	[3.306, 3.618]	43.539	< .0001
Marrow Donor	0.308 ***	0.075	[0.161, 0.455]	4.104	< .0001
Kidney Donor (D)	0.308 ***	0.075	[0.161, 0.455]	4.104	< .0001
Humanitarian Aid Worker	0.452 ***	0.075	[0.305, 0.599]	6.027	< .0001
Kidney Donor (ND)	0.346 ***	0.075	[0.199, 0.493]	4.617	< .0001
Heroic Rescuer	0.577 ***	0.075	[0.430, 0.724]	7.694	< .0001
Liver Donor	0.37 ***	0.075	[0.223, 0.517]	4.937	< .0001

Note. Groups are coded as indicator variables relative to the average person. Fixed-effects coefficients are unstandardized. SE indicates the standard error. 95% CI indicates lower/upper limits of the confidence interval. * indicates p<.05, ** indicates p<.01, *** indicates p<.001. P-values are two-tailed. Multivariate mixed-effects regressions compared ratings of all altruistic groups against ratings of the average person simultaneously (in lieu of separate tests for which corrections for multiple comparisons would be appropriate).

Table S17d-ii. Linear mixed-effects results for beliefs about altruistic groups' Honesty-Humility (Study 2b)

Honesty-Humility	Estimate	SE	95% CI	T	p
Intercept	3.264 ***	0.081	[3.104, 3.423]	40.099	< .0001
Marrow Donor	0.602 ***	0.069	[0.467, 0.737]	8.725	< .0001
Kidney Donor (D)	0.572 ***	0.069	[0.437, 0.708]	8.292	< .0001
Humanitarian Aid Worker	0.756 ***	0.069	[0.621, 0.892]	10.960	< .0001
Kidney Donor (ND)	0.592 ***	0.069	[0.457, 0.727]	8.580	< .0001
Heroic Rescuer	0.632 ***	0.069	[0.496, 0.767]	9.157	< .0001
Liver Donor	0.607 ***	0.069	[0.472, 0.742]	8.797	< .0001

Note. Groups are coded as indicator variables relative to the average person. Fixed-effects coefficients are unstandardized. SE indicates the standard error. 95% CI indicates lower/upper limits of the confidence interval. * indicates p<.05, ** indicates p<.01, *** indicates p<.001. P-values are two-tailed. Multivariate mixed-effects regressions compared ratings of all altruistic groups against ratings of the average person simultaneously (in lieu of separate tests for which corrections for multiple comparisons would be appropriate).

Table S17e-i. Linear mixed-effects results for beliefs about altruistic groups' Emotionality (Study 2a)

Emotionality	Estimate	SE	95% CI	T	p
Intercept	3.486 ***	0.083	[3.323, 3.649]	41.950	< .0001
Marrow Donor	0.115	0.078	[-0.037, 0.268]	1.484	0.138
Kidney Donor (D)	0.072	0.078	[-0.080, 0.225]	0.927	0.354
Humanitarian Aid Worker	0.188 *	0.078	[0.035, 0.340]	2.411	0.016
Kidney Donor (ND)	0.149	0.078	[-0.004, 0.302]	1.916	0.056
Heroic Rescuer	0.144	0.078	[-0.008, 0.297]	1.854	0.064
Liver Donor	0.168 *	0.078	[0.016, 0.321]	2.163	0.031

Note. Groups are coded as indicator variables relative to the average person. Fixed-effects coefficients are unstandardized. SE indicates the standard error. 95% CI indicates lower/upper limits of the confidence interval. * indicates p<.05, ** indicates p<.01, *** indicates p<.001. P-values are two-tailed. Multivariate mixed-effects regressions compared ratings of all altruistic groups against ratings of the average person simultaneously (in lieu of separate tests for which corrections for multiple comparisons would be appropriate).

Table S17e-ii. Linear mixed-effects results for beliefs about altruistic groups' Emotionality (Study 2b)

Emotionality	Estimate	SE	95% CI	T	p
Intercept	3.333 ***	0.085	[3.166, 3.501]	39.067	< .0001
Marrow Donor	0.159 *	0.071	[0.019, 0.299]	2.229	0.026
Kidney Donor (D)	0.134	0.071	[-0.006, 0.274]	1.880	0.06
Humanitarian Aid Worker	0.139	0.071	[-0.001, 0.279]	1.950	0.051
Kidney Donor (ND)	0.129	0.071	[-0.011, 0.270]	1.811	0.07
Heroic Rescuer	0.109	0.071	[-0.031, 0.250]	1.532	0.126
Liver Donor	0.134	0.071	[-0.006, 0.274]	1.880	0.06

Note. Groups are coded as indicator variables relative to the average person. Fixed-effects coefficients are unstandardized. SE indicates the standard error. 95% CI indicates lower/upper limits of the confidence interval. * indicates p<.05, ** indicates p<.01, *** indicates p<.001. P-values are two-tailed. Multivariate mixed-effects regressions compared ratings of all altruistic groups against ratings of the average person simultaneously (in lieu of separate tests for which corrections for multiple comparisons would be appropriate).

Table S17f-i. Linear mixed-effects results for beliefs about altruistic groups' Extraversion (Study 2a)

**Extraversion**	**Estimate**	**SE**	**95% CI**	**T**	**p**
Intercept	3.514 ***	0.072	[3.373, 3.656]	48.614	< .0001
Marrow Donor	0.067	0.077	[-0.084, 0.218]	0.874	0.383
Kidney Donor (D)	0.125	0.077	[-0.026, 0.276]	1.622	0.105
Humanitarian Aid Worker	0.341 ***	0.077	[0.190, 0.493]	4.430	< .0001
Kidney Donor (ND)	0.048	0.077	[-0.103, 0.199]	0.624	0.533
Heroic Rescuer	0.38 ***	0.077	[0.229, 0.531]	4.929	< .0001
Liver Donor	0.082	0.077	[-0.069, 0.233]	1.061	0.289

Note. Groups are coded as indicator variables relative to the average person. Fixed-effects coefficients are unstandardized. SE indicates the standard error. 95% CI indicates lower/upper limits of the confidence interval. * indicates p<.05, ** indicates p<.01, *** indicates p<.001. P-values are two-tailed. Multivariate mixed-effects regressions compared ratings of all altruistic groups against ratings of the average person simultaneously (in lieu of separate tests for which corrections for multiple comparisons would be appropriate).

Table S17f-ii. Linear mixed-effects results for beliefs about altruistic groups' Extraversion (Study 2b)

**Extraversion**	**Estimate**	**SE**	**95% CI**	**T**	**p**
Intercept	3.239 ***	0.076	[3.090, 3.387]	42.788	< .0001
Marrow Donor	0.338 ***	0.070	[0.202, 0.475]	4.860	< .0001
Kidney Donor (D)	0.274 ***	0.070	[0.137, 0.410]	3.931	< .0001
Humanitarian Aid Worker	0.637 ***	0.070	[0.500, 0.773]	9.148	< .0001
Kidney Donor (ND)	0.323 ***	0.070	[0.187, 0.460]	4.645	< .0001
Heroic Rescuer	0.672 ***	0.070	[0.535, 0.808]	9.648	< .0001
Liver Donor	0.348 ***	0.070	[0.212, 0.485]	5.003	< .0001

Note. Groups are coded as indicator variables relative to the average person. Fixed-effects coefficients are unstandardized. SE indicates the standard error. 95% CI indicates lower/upper limits of the confidence interval. * indicates p<.05, ** indicates p<.01, *** indicates p<.001. P-values are two-tailed. Multivariate mixed-effects regressions compared ratings of all altruistic groups against ratings of the average person simultaneously (in lieu of separate tests for which corrections for multiple comparisons would be appropriate).

Table S17g-i. Linear mixed-effects results for beliefs about altruistic groups' Agreeableness (Study 2a)

Agreeableness	Estimate	SE	95% CI	T	p
Intercept	3.519 ***	0.071	[3.380, 3.658]	49.625	< .0001
Marrow Donor	0.322 ***	0.074	[0.178, 0.467]	4.375	< .0001
Kidney Donor (D)	0.346 ***	0.074	[0.202, 0.491]	4.702	< .0001
Humanitarian Aid Worker	0.466 ***	0.074	[0.322, 0.611]	6.334	< .0001
Kidney Donor (ND)	0.351 ***	0.074	[0.207, 0.495]	4.767	< .0001
Heroic Rescuer	0.534 ***	0.074	[0.389, 0.678]	7.248	< .0001
Liver Donor	0.341 ***	0.074	[0.197, 0.486]	4.636	< .0001

Note. Groups are coded as indicator variables relative to the average person. Fixed-effects coefficients are unstandardized. SE indicates the standard error. 95% CI indicates lower/upper limits of the confidence interval. * indicates p<.05, ** indicates p<.01, *** indicates p<.001. P-values are two-tailed. Multivariate mixed-effects regressions compared ratings of all altruistic groups against ratings of the average person simultaneously (in lieu of separate tests for which corrections for multiple comparisons would be appropriate).

Table S17g-ii. Linear mixed-effects results for beliefs about altruistic groups' Agreeableness (Study 2b)

Agreeableness	Estimate	SE	95% CI	T	p
Intercept	3.388 ***	0.075	[3.241, 3.535]	45.112	< .0001
Marrow Donor	0.418 ***	0.067	[0.286, 0.550]	6.208	< .0001
Kidney Donor (D)	0.353 ***	0.067	[0.221, 0.485]	5.247	< .0001
Humanitarian Aid Worker	0.542 ***	0.067	[0.410, 0.674]	8.056	< .0001
Kidney Donor (ND)	0.388 ***	0.067	[0.256, 0.520]	5.765	< .0001
Heroic Rescuer	0.532 ***	0.067	[0.400, 0.664]	7.908	< .0001
Liver Donor	0.468 ***	0.067	[0.336, 0.600]	6.947	< .0001

Note. Groups are coded as indicator variables relative to the average person. Fixed-effects coefficients are unstandardized. SE indicates the standard error. 95% CI indicates lower/upper limits of the confidence interval. * indicates p<.05, ** indicates p<.01, *** indicates p<.001. P-values are two-tailed. Multivariate mixed-effects regressions compared ratings of all altruistic groups against ratings of the average person simultaneously (in lieu of separate tests for which corrections for multiple comparisons would be appropriate).

Table S17h-i. Linear mixed-effects results for beliefs about altruistic groups' Conscientiousness (Study 2a)

Conscientiousness	Estimate	SE	95% CI	T	p
Intercept	3.577 ***	0.073	[3.433, 3.721]	48.669	< .0001
Marrow Donor	0.356 ***	0.080	[0.200, 0.512]	4.469	< .0001
Kidney Donor (D)	0.332 ***	0.080	[0.176, 0.488]	4.167	< .0001
Humanitarian Aid Worker	0.471 ***	0.080	[0.315, 0.627]	5.918	< .0001
Kidney Donor (ND)	0.245 **	0.080	[0.089, 0.401]	3.080	0.002
Heroic Rescuer	0.481 ***	0.080	[0.325, 0.637]	6.039	< .0001
Liver Donor	0.341 ***	0.080	[0.185, 0.498]	4.287	< .0001

Note. Groups are coded as indicator variables relative to the average person. Fixed-effects coefficients are unstandardized. SE indicates the standard error. 95% CI indicates lower/upper limits of the confidence interval. * indicates p<.05, ** indicates p<.01, *** indicates p<.001. P-values are two-tailed. Multivariate mixed-effects regressions compared ratings of all altruistic groups against ratings of the average person simultaneously (in lieu of separate tests for which corrections for multiple comparisons would be appropriate).

Table S17h-ii. Linear mixed-effects results for beliefs about altruistic groups' Conscientiousness (Study 2b)

Conscientiousness	Estimate	SE	95% CI	T	p
Intercept	3.378 ***	0.074	[3.234, 3.522]	45.902	< .0001
Marrow Donor	0.532 ***	0.067	[0.401, 0.664]	7.950	< .0001
Kidney Donor (D)	0.458 ***	0.067	[0.326, 0.589]	6.835	< .0001
Humanitarian Aid Worker	0.716 ***	0.067	[0.585, 0.848]	10.699	< .0001
Kidney Donor (ND)	0.418 ***	0.067	[0.287, 0.549]	6.241	< .0001
Heroic Rescuer	0.657 ***	0.067	[0.525, 0.788]	9.807	< .0001
Liver Donor	0.453 ***	0.067	[0.321, 0.584]	6.761	< .0001

Note. Groups are coded as indicator variables relative to the average person. Fixed-effects coefficients are unstandardized. SE indicates the standard error. 95% CI indicates lower/upper limits of the confidence interval. * indicates p<.05, ** indicates p<.01, *** indicates p<.001. P-values are two-tailed. Multivariate mixed-effects regressions compared ratings of all altruistic groups against ratings of the average person simultaneously (in lieu of separate tests for which corrections for multiple comparisons would be appropriate).

Table S17i-i. Linear mixed-effects results for beliefs about altruistic groups' Openness to Experience (Study 2a)

Openness to Experience	Estimate	SE	95% CI	T	p
Intercept	3.567 ***	0.073	[3.424, 3.710]	48.934	< .0001
Marrow Donor	0.226 **	0.073	[0.083, 0.369]	3.095	0.002
Kidney Donor (D)	0.144 *	0.073	[0.001, 0.287]	1.975	0.048
Humanitarian Aid Worker	0.375 ***	0.073	[0.232, 0.518]	5.136	< .0001
Kidney Donor (ND)	0.187 *	0.073	[0.044, 0.331]	2.568	0.01
Heroic Rescuer	0.389 ***	0.073	[0.246, 0.533]	5.334	< .0001
Liver Donor	0.197 **	0.073	[0.054, 0.340]	2.700	0.007

Note. Groups are coded as indicator variables relative to the average person. Fixed-effects coefficients are unstandardized. SE indicates the standard error. 95% CI indicates lower/upper limits of the confidence interval. * indicates p<.05, ** indicates p<.01, *** indicates p<.001. P-values are two-tailed. Multivariate mixed-effects regressions compared ratings of all altruistic groups against ratings of the average person simultaneously (in lieu of separate tests for which corrections for multiple comparisons would be appropriate).

Table S17i-ii. Linear mixed-effects results for beliefs about altruistic groups' Openness to Experience (Study 2b)

Openness to Experience	Estimate	SE	95% CI	T	p
Intercept	3.363 ***	0.071	[3.224, 3.503]	47.338	< .0001
Marrow Donor	0.313 ***	0.066	[0.185, 0.442]	4.783	< .0001
Kidney Donor (D)	0.234 ***	0.066	[0.105, 0.362]	3.568	< .0001
Humanitarian Aid Worker	0.652 ***	0.066	[0.523, 0.780]	9.946	< .0001
Kidney Donor (ND)	0.259 ***	0.066	[0.130, 0.387]	3.948	< .0001
Heroic Rescuer	0.443 ***	0.066	[0.314, 0.571]	6.757	< .0001
Liver Donor	0.284 ***	0.066	[0.155, 0.412]	4.328	< .0001

Note. Groups are coded as indicator variables relative to the average person. Fixed-effects coefficients are unstandardized. SE indicates the standard error. 95% CI indicates lower/upper limits of the confidence interval. * indicates p<.05, ** indicates p<.01, *** indicates p<.001. P-values are two-tailed. Multivariate mixed-effects regressions compared ratings of all altruistic groups against ratings of the average person simultaneously (in lieu of separate tests for which corrections for multiple comparisons would be appropriate).

Table S18a-i. Linear mixed-effects results for beliefs about Miss America Contestants' Altruism

Altruism	Estimate	SE	95% CI	T	p
Intercept	2.701 ***	0.088	[2.528, 2.875]	30.621	< .0001
Miss America Contestant	-0.408 ***	0.084	[-0.573, -0.243]	-4.866	< .0001

Note. Groups are coded as indicator variables relative to the average person. Fixed-effects coefficients are unstandardized. SE indicates the standard error. 95% CI indicates lower/upper limits of the confidence interval. * indicates p<.05, ** indicates p<.01, *** indicates p<.001. P-values are two-tailed.

Table S18a-ii. Linear mixed-effects results for beliefs about BASE Jumpers' Altruism

Altruism	Estimate	SE	95% CI	T	p
Intercept	2.701 ***	0.097	[2.511, 2.892]	27.892	< .0001
BASE Jumper	-0.224 *	0.113	[-0.446, -0.002]	-1.986	0.048

Note. Groups are coded as indicator variables relative to the average person. Fixed-effects coefficients are unstandardized. SE indicates the standard error. 95% CI indicates lower/upper limits of the confidence interval. * indicates p<.05, ** indicates p<.01, *** indicates p<.001. P-values are two-tailed.

Table S18b-i. Linear mixed-effects results for beliefs about Miss America Contestants' Risk

Risk	Estimate	SE	95% CI	T	p
Intercept	2.368 ***	0.092	[2.187, 2.550]	25.706	< .0001
Miss America Contestant	-0.204 *	0.080	[-0.361, -0.047]	-2.563	0.011

Note. Groups are coded as indicator variables relative to the average person. Fixed-effects coefficients are unstandardized. SE indicates the standard error. 95% CI indicates lower/upper limits of the confidence interval. * indicates p<.05, ** indicates p<.01, *** indicates p<.001. P-values are two-tailed.

Table S18b-ii. Linear mixed-effects results for beliefs about BASE Jumpers' Risk

Risk	Estimate	SE	95% CI	T	p
Intercept	2.368 ***	0.088	[2.194, 2.542]	26.874	< .0001
BASE Jumper	1.881 ***	0.125	[1.635, 2.126]	15.091	< .0001

Note. Groups are coded as indicator variables relative to the average person. Fixed-effects coefficients are unstandardized. SE indicates the standard error. 95% CI indicates lower/upper limits of the confidence interval. * indicates p<.05, ** indicates p<.01, *** indicates p<.001. P-values are two-tailed.

Table S18c-i. Linear mixed-effects results for beliefs about Miss America Contestants' Honesty-Humility

Honesty-Humility	Estimate	SE	95% CI	T	p
Intercept	3.264 ***	0.079	[3.108, 3.420]	41.245	< .0001
Miss America Contestant	-0.209 *	0.087	[-0.380, -0.038]	-2.414	0.017

Note. Groups are coded as indicator variables relative to the average person. Fixed-effects coefficients are unstandardized. SE indicates the standard error. 95% CI indicates lower/upper limits of the confidence interval. * indicates p<.05, ** indicates p<.01, *** indicates p<.001. P-values are two-tailed.

Table S18c-ii. Linear mixed-effects results for beliefs about BASE Jumpers' Honesty-Humility

Honesty-Humility	Estimate	SE	95% CI	T	p
Intercept	3.264 ***	0.083	[3.101, 3.427]	39.472	< .0001
BASE Jumper	-0.468 ***	0.093	[-0.651, -0.285]	-5.041	< .0001

Note. Groups are coded as indicator variables relative to the average person. Fixed-effects coefficients are unstandardized. SE indicates the standard error. 95% CI indicates lower/upper limits of the confidence interval. * indicates p<.05, ** indicates p<.01, *** indicates p<.001. P-values are two-tailed.

Table S18d-i. Linear mixed-effects results for beliefs about Miss America Contestants' Emotionality

Emotionality	Estimate	SE	95% CI	T	p
Intercept	3.333 ***	0.074	[3.188, 3.479]	45.138	< .0001
Miss America Contestant	-0.119	0.084	[-0.285, 0.046]	-1.423	0.156

Note. Groups are coded as indicator variables relative to the average person. Fixed-effects coefficients are unstandardized. SE indicates the standard error. 95% CI indicates lower/upper limits of the confidence interval. * indicates p<.05, ** indicates p<.01, *** indicates p<.001. P-values are two-tailed.

Table S18d-ii. Linear mixed-effects results for beliefs about BASE Jumpers' Emotionality

Emotionality	Estimate	SE	95% CI	T	p
Intercept	3.333 ***	0.082	[3.172, 3.495]	40.761	< .0001
BASE Jumper	-0.706 ***	0.105	[-0.914, -0.499]	-6.710	< .0001

Note. Groups are coded as indicator variables relative to the average person. Fixed-effects coefficients are unstandardized. SE indicates the standard error. 95% CI indicates lower/upper limits of the confidence interval. * indicates p<.05, ** indicates p<.01, *** indicates p<.001. P-values are two-tailed.

Table S18e-i. Linear mixed-effects results for beliefs about Miss America Contestants' Extraversion

Extraversion	Estimate	SE	95% CI	T	p
Intercept	3.239 ***	0.071	[3.099, 3.379]	45.717	< .0001
Miss America Contestant	0.756 ***	0.100	[0.559, 0.953]	7.571	< .0001

Note. Groups are coded as indicator variables relative to the average person. Fixed-effects coefficients are unstandardized. SE indicates the standard error. 95% CI indicates lower/upper limits of the confidence interval. * indicates p<.05, ** indicates p<.01, *** indicates p<.001. P-values are two-tailed.

Table S18e-ii. Linear mixed-effects results for beliefs about BASE Jumpers' Extraversion

Extraversion	Estimate	SE	95% CI	T	p
Intercept	3.239 ***	0.077	[3.087, 3.391]	41.994	< .0001
BASE Jumper	0.313 **	0.108	[0.100, 0.527]	2.895	0.004

Note. Groups are coded as indicator variables relative to the average person. Fixed-effects coefficients are unstandardized. SE indicates the standard error. 95% CI indicates lower/upper limits of the confidence interval. * indicates p<.05, ** indicates p<.01, *** indicates p<.001. P-values are two-tailed.

Table S18f-i. Linear mixed-effects results for beliefs about Miss America Contestants' Agreeableness

Agreeableness	Estimate	SE	95% CI	T	p
Intercept	3.388 ***	0.075	[3.240, 3.536]	45.035	< .0001
Miss America Contestant	-0.045	0.093	[-0.229, 0.139]	-0.479	0.632

Note. Groups are coded as indicator variables relative to the average person. Fixed-effects coefficients are unstandardized. SE indicates the standard error. 95% CI indicates lower/upper limits of the confidence interval. * indicates p<.05, ** indicates p<.01, *** indicates p<.001. P-values are two-tailed.

Table S18f-ii. Linear mixed-effects results for beliefs about BASE Jumpers' Agreeableness

Agreeableness	Estimate	SE	95% CI	T	p
Intercept	3.388 ***	0.075	[3.241, 3.535]	45.357	< .0001
BASE Jumper	-0.363 ***	0.090	[-0.541, -0.185]	-4.018	< .0001

Note. Groups are coded as indicator variables relative to the average person. Fixed-effects coefficients are unstandardized. SE indicates the standard error. 95% CI indicates lower/upper limits of the confidence interval. * indicates p<.05, ** indicates p<.01, *** indicates p<.001. P-values are two-tailed.

Table S18g-i. Linear mixed-effects results for beliefs about Miss America Contestants' Conscientiousness

Conscientiousness	Estimate	SE	95% CI	T	p
Intercept	3.378 ***	0.076	[3.227, 3.529]	44.161	< .0001
Miss America Contestant	0.194 *	0.096	[0.006, 0.383]	2.030	0.044

Note. Groups are coded as indicator variables relative to the average person. Fixed-effects coefficients are unstandardized. SE indicates the standard error. 95% CI indicates lower/upper limits of the confidence interval. * indicates p<.05, ** indicates p<.01, *** indicates p<.001. P-values are two-tailed.

Table S18g-ii. Linear mixed-effects results for beliefs about BASE Jumpers' Conscientiousness

Conscientiousness	Estimate	SE	95% CI	T	p
Intercept	3.378 ***	0.079	[3.222, 3.535]	42.56	< .0001
BASE Jumper	0	0.102	[-0.201, 0.201]	0.00	1

Note. Groups are coded as indicator variables relative to the average person. Fixed-effects coefficients are unstandardized. SE indicates the standard error. 95% CI indicates lower/upper limits of the confidence interval. * indicates p<.05, ** indicates p<.01, *** indicates p<.001. P-values are two-tailed.

Table S18h-i. Linear mixed-effects results for beliefs about Miss America Contestants' Openness to Experience

Openness to Experience	Estimate	SE	95% CI	T	p
Intercept	3.363 ***	0.072	[3.221, 3.505]	46.687	< .0001
Miss America Contestant	0.224 **	0.084	[0.058, 0.389]	2.669	0.008

Note. Groups are coded as indicator variables relative to the average person. Fixed-effects coefficients are unstandardized. SE indicates the standard error. 95% CI indicates lower/upper limits of the confidence interval. * indicates p<.05, ** indicates p<.01, *** indicates p<.001. P-values are two-tailed.

Table S18h-ii. Linear mixed-effects results for beliefs about BASE Jumpers' Openness to Experience

Openness to Experience	Estimate	SE	95% CI	T	p
Intercept	3.363 ***	0.078	[3.209, 3.517]	43.057	< .0001
BASE Jumper	-0.164	0.099	[-0.360, 0.031]	-1.655	0.099

Note. Groups are coded as indicator variables relative to the average person. Fixed-effects coefficients are unstandardized. SE indicates the standard error. 95% CI indicates lower/upper limits of the confidence interval. * indicates p<.05, ** indicates p<.01, *** indicates p<.001. P-values are two-tailed.
