# Supplementary material for: Unselfish traits and social decision-making patterns characterize six populations of real-world extraordinary altruists
Source: Nat Commun. 2023 Mar 31;14:1807. doi: 10.1038/s41467-023-37283-5 (PMC10066349; doi:10.1038/s41467-023-37283-5)
Supplement: Supplementary file 3 — Reporting Summary [file 41467_2023_37283_MOESM3_ESM.pdf]

nature portfolio

Corresponding author(s): Shawn Rhoads
Last updated by author(s): Dec 19, 2022

Reporting Summary

Nature Portfolio wishes to improve the reproducibility of the work that we publish. This form provides structure for consistency and transparency in reporting. For further information on Nature Portfolio policies, see our Editorial Policies and the Editorial Policy Checklist.

Statistics

For all statistical analyses, confirm that the following items are present in the figure legend, table legend, main text, or Methods section.

n/a	Confirmed	
☐	☒	The exact sample size (*n*) for each experimental group/condition, given as a discrete number and unit of measurement
☐	☒	A statement on whether measurements were taken from distinct samples or whether the same sample was measured repeatedly
☐	☒	The statistical test(s) used AND whether they are one- or two-sided<br>*Only common tests should be described solely by name; describe more complex techniques in the Methods section.*
☐	☒	A description of all covariates tested
☐	☒	A description of any assumptions or corrections, such as tests of normality and adjustment for multiple comparisons
☐	☒	A full description of the statistical parameters including central tendency (e.g. means) or other basic estimates (e.g. regression coefficient) AND variation (e.g. standard deviation) or associated estimates of uncertainty (e.g. confidence intervals)
☐	☒	For null hypothesis testing, the test statistic (e.g. *F*, *t*, *r*) with confidence intervals, effect sizes, degrees of freedom and *P* value noted<br>*Give P values as exact values whenever suitable.*
☒	☐	For Bayesian analysis, information on the choice of priors and Markov chain Monte Carlo settings
☐	☒	For hierarchical and complex designs, identification of the appropriate level for tests and full reporting of outcomes
☐	☒	Estimates of effect sizes (e.g. Cohen's *d*, Pearson's *r*), indicating how they were calculated

Our web collection on statistics for biologists contains articles on many of the points above.

Software and code

Policy information about availability of computer code

Data collection	Qualtrics online software was used to collect the data for Studies 1ab and 2abc. The Lee & Ashton (2020) data were collected by Lee & Ashton using a survey posted to https://hexaco.org/
Data analysis	Study 1ab: To assess how individuals in each of the altruistic groups differed from typical adults across these 24 measures, we conducted a series of linear ordinary least squares regressions nlme package (v 3.1-152) in R version 3.6.3. To test whether our finding that Honesty-Humility is the dimension of the HEXACO that most reliably distinguishes real-world extraordinary altruists would replicate in an independent control dataset, we wrote custom code in Python 3.7.6 using numpy (v 1.18.1), pandas (v 1.1.5), and scipy (v 1.4.1). To analyze social discounting data, we used the nlme package (https://cran.r-project.org/package=nlme) in R version 3.6.3 to fit data to a hyperbolic mixed-effects model. For the classification analysis, we used the caret package (v 6.0-88) in R version 3.6.3.<br><br>Study 2abc: For the linear mixed-effects regressions, we used the nlme package (v 3.1-152) in R version 3.6.3. For analyses of rare non-altruistic groups, we used the same custom code in Python 3.7.6 for the bootstrapping procedure. For representational similarity analysis, we wrote custom code in Python 3.7.6 using using numpy (v 1.18.1), pandas (v 1.1.5), and scipy (v 1.4.1).<br><br>Jupyter Notebooks for code in R/Python are shared on the Open Science Framework at https://osf.io/7t4qf/

For manuscripts utilizing custom algorithms or software that are central to the research but not yet described in published literature, software must be made available to editors and reviewers. We strongly encourage code deposition in a community repository (e.g. GitHub). See the Nature Portfolio guidelines for submitting code & software for further information.

Data

Policy information about availability of data

All manuscripts must include a data availability statement. This statement should provide the following information, where applicable:

- Accession codes, unique identifiers, or web links for publicly available datasets
- A description of any restrictions on data availability
- For clinical datasets or third party data, please ensure that the statement adheres to our policy

The processed data generated in this study are provided with this paper and deposited on the Open Science Framework [https://osf.io/7t4qf/]. The raw data are protected and are not available due to data privacy laws. The bootstrap samples in study 1 were generated from data used in Lee & Ashton (2020) and Lee & Ashton (2018) with permission from the authors. To access these data, please refer to those studies.

Human research participants

Policy information about studies involving human research participants and Sex and Gender in Research.

Reporting on sex and gender	We report gender proportions for each sample. Analyses in study 1 included gender as covariates in the models.
Population characteristics	See below.
Recruitment	Study 1: Altruistic and directed kidney donors, liver donors, and bone marrow or hematopoietic stem cell donors were recruited and invited to participate through communications via local and national transplant organizations (Transplant Village, National Marrow Donor Program; invitations sent to individuals who completed a donation) and our existing database of altruistic kidney donors. Humanitarian aid workers were recruited through online postings in Facebook groups of individuals affiliated with Doctors Without Borders, Médicins Sans Frontiere, as well as email communications sent out to staff and volunteers of International Medical Corps. Heroic rescuers were recruited through email invitations from the Carnegie Hero Fund to members of their internal database. Control participants were recruited from the local community, through flyers and postings on Research Match, and were included in the sample as long as they did not meet criteria for any of the above altruist categories. We also conducted supplemental analyses of 5,000 bootstrap samples drawn from a large population of 347,192 adults (including 158,130 U.S. adults), who were matched to altruists on age and sex using data from Lee & Ashton (2020). These participants were recruited through an online survey site (https://hexaco.org) from October 19, 2014 to October 18, 2018.<br><br>Study 2ab: Participants were a sample of American adults and recruited using a Qualtrics panel designed to match census-based population demographics in terms of sex, age, race and ethnicity, and education: sex (51% Female), age (30.5% 18-34 years, 34.4% 35-54 years, 35.2% 55+ years), race/ethnicity (62.3% non-Hispanic white, 12.4% non-Hispanic black, 17.3% Hispanic, 5.4% Asian, 2.6% other), and education (24% HS degree or less, 48% Bachelors or some college, 28% graduate or professional degree).<br><br>Study 2c: We recruited two independent samples of rare populations (N=28), including extreme athletes (e.g., BASE Jumpers) and former contestants in the national Miss America pageant. Recruitment of BASE Jumpers was carried out using previous recruitment pools from researchers who have assessed personality traits of these athletes as well as online public forums (e.g., https://www.blincmagazine.com/forum/). Recruitment of Miss America contestants was carried out via snowball sampling and via online forums (e.g., Facebook groups).
Ethics oversight	Study 1 and Study 2c were approved by the Georgetown University IRB. Studies 2ab were approved by the Linfield University IRB.

Note that full information on the approval of the study protocol must also be provided in the manuscript.

Field-specific reporting

Please select the one below that is the best fit for your research. If you are not sure, read the appropriate sections before making your selection.

☐ Life sciences ☒ Behavioural & social sciences ☐ Ecological, evolutionary & environmental sciences

For a reference copy of the document with all sections, see nature.com/documents/nr-reporting-summary-flat.pdf

Behavioural & social sciences study design

All studies must disclose on these points even when the disclosure is negative.

Study description	Quantitative (experimental and non-experimental)

Research sample

Samples of altruists and typical adults were selected to test our hypotheses about features of extraordinary altruists as compared to typical adults. We recruited individuals who had engaged in acts of extraordinary altruism that are extremely rare. These altruists are demographically non-representative of US adults. In response to reviewer requests, we also collected data from equally rare non-altruist populations (BASE jumpers and Miss America contestants) who are also not demographically representative.

We also recruited a control sample to approximately match our altruistic sample demographically.

We also analyzed data from a large US sample collected by Lee & Ashton (2020) that is not demographically representative.

Finally, we recruited online panels of adults for Studies 2a and 2b who were stratified to be approximately demographically representative.

The samples in studies 1ab were drawn from populations of individuals who had engaged in altruistic acts that are normatively very rare (<.00005% annual prevalence rate per capita in United States): 347 altruists included heroic rescuers (N=27; Mage=50.22, SEage=.2.41; 1 Female; annual U.S. prevalence: 0.00000024%), who had received a Carnegie Medal for "risking their lives to an extraordinary degree saving or attempting to save the lives of others"; non-directed (altruistic) kidney donors (N=132; Mage=46.53, SEage=1.02; 86 Females; prevalence: 0.00000089%) who had donated a kidney to an anonymous stranger; directed kidney donors (N=68; Mage=46.13, SEage=1.56; 54 Females; prevalence: 0.00000906%) who had donated a kidney to a specified other person; liver donors (N=12, 8 directed and 3 non-directed; Mage=39.83, SEage=2.34; 8 Females; prevalence: 0.00000113%); bone marrow or hematopoietic stem cell donors (N=55; Mage=36.18, SEage=1.51; 26 Females; prevalence: 0.00001530%) who had donated bone marrow to an unspecified stranger; and humanitarian aid workers (N=53; Mage=41.49, SEage=1.62; 36 Females; prevalence: 0.00000128%) who had performed work for organizations such as Médicins Sans Frontieres (Doctors Without Borders; participants included 41 North American respondents as well as 12 from other locales). In addition, 207 control participants (Mage=37.72, SEage=.63; 134 Females) were recruited from the local community through flyers and postings on Research Match and confirmed they did not meet criteria for any of the above altruist categories.

In study 2a, we recruited a representative sample of American adults (N=208) using a Qualtrics panel, which approximately corresponded to target demographics in terms of gender (50% female, 48.1% male, 1.9% other), age (30.3% 18-34 years, 34.1% 35-54 years, 35.6% 55+ years), race/ethnicity (67.3% non-Hispanic white, 13% non-Hispanic black, 11.5% Hispanic, 5.3% Asian, 2.9% other), and education (21.6% HS degree or less, 54.8% Bachelors or some college, 23.6% graduate or professional degree).

In study 2b, we recruited a representative sample of American adults (N=201) using a Qualtrics panel, which approximately corresponded to target demographics in terms of gender (50.25% female, 50.25% male, 0.5% other), age (30.85% 18-34 years, 33.83% 35-54 years, 35.32% 55+ years), race/ethnicity (60.2% non-Hispanic white, 12.44% non-Hispanic black, 16.92% Hispanic or Latino, 4.98% Asian, 5.47% other), and education (23.88% HS degree or less, 47.76% Bachelors or some college, 28.36% graduate or professional degree).

In study 2c, we recruited two independent samples of rare populations (N=28), including extreme athletes (e.g., BASE Jumpers) and former contestants in the national Miss America pageant. Recruitment of BASE Jumpers was carried out using previous recruitment pools from researchers who have assessed personality traits of these athletes as well as online public forums (e.g., https://www.blincmagazine.com/forum/). Recruitment of Miss America contestants was carried out via snowball sampling and via online forums (e.g., Facebook groups).

Sampling strategy

Altruistic and directed kidney donors, liver donors, and bone marrow or hematopoietic stem cell donors were recruited and invited to participate through communications via local and national transplant organizations (Transplant Village, National Marrow Donor Program; invitations sent to individuals who completed a donation) and our existing database of altruistic kidney donors. Humanitarian aid workers were recruited through online postings in Facebook groups of individuals affiliated with Doctors Without Borders, Médicins Sans Frontiere, as well as email communications sent out to staff and volunteers of International Medical Corps. Heroic rescuers were recruited through email invitations from the Carnegie Hero Fund to members of their internal database. These populations are extremely rare and we recruited as many participants as possible from these extremely small populations of altruists using multiple recruitment approaches, including asking partner organizations to invite members to participate, as well as snowball sampling. All eligible participants recruited this way were included. Control participants were recruited from the local community (convenience sample), through flyers and postings on Research Match. Extensive screening confirmed they did not meet criteria for any of the above altruist categories. Each bootstrap sample generated from the Lee & Ashton (2020) dataset included participants that fell into the range of altruists' age, sex, and country demographics.

Study 2ab participants were recruited by Qualtrics from a panel of potential U.S. participants and were selected to match census-based U.S. population demographics: gender (51% Female), age (30.5% 18-34 years, 34.4% 35-54 years, 35.2% 55+ years), race/ethnicity (62.3% non-Hispanic white, 12.4% non-Hispanic black, 17.3% Hispanic, 5.4% Asian, 2.6% other), and education (24% HS degree or less, 48% Bachelors or some college, 28% graduate or professional degree).

In Study 2c, BASE jumper participants were recruited using previous recruitment pools from researchers who have assessed personality traits of these athletes as well as online public forums (e.g., https://www.blincmagazine.com/forum/). Recruitment of Miss America contestants was carried out via snowball sampling and via online forums (e.g., Facebook groups).

Data collection	Data collection occurred via computer on Qualtrics. The researchers were not present during completion of these surveys. Researchers were not blinded to the study hypotheses.
Timing	Data collection for study 1 took place from September 2017 until August 2019. The Lee & Ashton (2020) data were collected between October 2014 and October 2018.<br><br>Data collection for study 2 took place from December 2020 until August 2022.
Data exclusions	Data were list-wise excluded from analyses in study 1 if they did not complete one of the measures of interest (e.g., one participant was not included in analyses involving the HEXACO measure of personality for not completing all items in that measure, but was included for other analyses) or if their data was unusable due to inconsistent responding (e.g., 16 participants were excluded in the social discounting task for switching between sharing and keeping amounts of money more than two times within one or more task blocks which prevents the estimation of their indifference point).<br><br>In study 2a, we recruited this representative sample while concurrently accounting for participants that that failed to pass an attention check that explicitly asked them to choose specific responses. We excluded 111 participants based on this criterion.<br><br>All participants in studies 2bc met inclusion criteria.
Non-participation	263 participants from study 1a did not opt-in to participate in study 1b.
Randomization	No randomization in original samples. Statistical models controlled for age, gender, household income, and education level.<br><br>To generate demographically-matched control samples for each of the rare groups (e.g., altruists, etc.), we used a bootstrap sampling procedure. We used data from a large population of 347,192 participants who completed the same HEXACO items measured in the present study (for recruitment methodology, see Lee & Ashton, 2020). Although participants from Lee & Ashton completed the 100-item version of the HEXACO, we only used their scores computed from the same 60 items in the HEXACO-PI-R. We stratified the international dataset by country (United States), age (quantile split), and gender, and randomly drew 5,000 bootstrap samples without replacement that were matched on each of these demographic variables to the Altruist, Miss America contestant, and BASE Jumper samples, respectively.

Reporting for specific materials, systems and methods

We require information from authors about some types of materials, experimental systems and methods used in many studies. Here, indicate whether each material, system or method listed is relevant to your study. If you are not sure if a list item applies to your research, read the appropriate section before selecting a response.

Materials & experimental systems

n/a	Involved in the study
☒	☐ Antibodies
☒	☐ Eukaryotic cell lines
☒	☐ Palaeontology and archaeology
☒	☐ Animals and other organisms
☒	☐ Clinical data
☒	☐ Dual use research of concern

Methods

n/a	Involved in the study
☒	☐ ChIP-seq
☒	☐ Flow cytometry
☒	☐ MRI-based neuroimaging
